# Synthesis and Biological Evaluation of Spirocyclic Chromane Derivatives as a Potential Treatment of Prostate Cancer

**DOI:** 10.3390/molecules26113162

**Published:** 2021-05-25

**Authors:** Li Feng, Shujia Yu, Hai Wang, Shengwei Yang, Xue Li, Hongjuan Dai, Liwen Zhao, Cheng Jiang, Yazhou Wang

**Affiliations:** 1 Department of Medicinal Chemistry, China Pharmaceutical University, Tongjiaxiang 24, Nanjing 210009, China; fengli@sanhome.com; 2 Nanjing Sanhome Pharmaceutical Co. Ltd., No. 99, West Yunlianghe Road, Jiangning District, Nanjing 210049, China; yusj@sanhome.com (S.Y.); wanghai@sanhome.com (H.W.); yangsw@sanhome.com (S.Y.); lixueyf@sanhome.com (X.L.); 3 Quality Department, Aurovitas Pharma Taizhou Co. Ltd., Taizhou 225300, China; hhitdhj@sohu.com

**Keywords:** p300/CBP, HAT inhibitors, antitumor activity

## Abstract

As a significant co-activator involved in cell cycle and cell growth, differentiation and development, p300/CBP has shown extraordinary potential target in cancer therapy. Herein we designed new compounds from the lead compound A-485 based on molecular dynamic simulations. A series of new spirocyclic chroman derivatives was prepared, characterized and proven to be a potential treatment of prostate cancer. The most potent compound **B16** inhibited the proliferation of enzalutamide-resistant 22Rv1 cells with an IC_50_ value of 96 nM. Furthermore, compounds **B16**–**P2** displayed favorable overall pharmacokinetic profiles, and better tumor growth inhibition than A-485 in an in vivo xenograft model.

## 1. Introduction

The acetylation and deacetylation of histones is a kind of reversible post-translational modification (PTM), which plays an important role in the regulation of gene expression in eukaryotic cells and is the arena of epigenetics [1,2,3]. The modification of the highly flexible and modifiable *N*-terminus of histones changes the state of histones, leading to genetic information transmission such as chromatin formation, transcription and DNA replication [4].

Histone acetyltransferase (HAT) and histone deacetylase (HDAC) are capable of affecting acetylation and deacetylation of histones, whose recruitment and normal function is the key regulatory step for gene expression and the cell cycle [3]. Functional deficiency of these enzymes may result in numerous diseases including tumors [5,6,7]. E1A binding protein of 300 kDa (p300) and CREB binding protein (CBP) are two main members of the HAT family. p300/CBP is involved in cell cycle progression and cell growth, differentiation and development, and is a significant co-activator [8,9,10,11,12,13]. By promoting histone acetylation to form an open chromatin structure, the transcription structure can be easily combined with chromatin to improve transcription activity [1,14]. Research showed that p300/CBP is highly expressed and activated in many different diseases, especially malignant tumor [5,9,15,16]. As a result, it is of great importance to develop p300/CBP inhibitors with high selectivity and potency [14,15,16].

**Figure 1:**
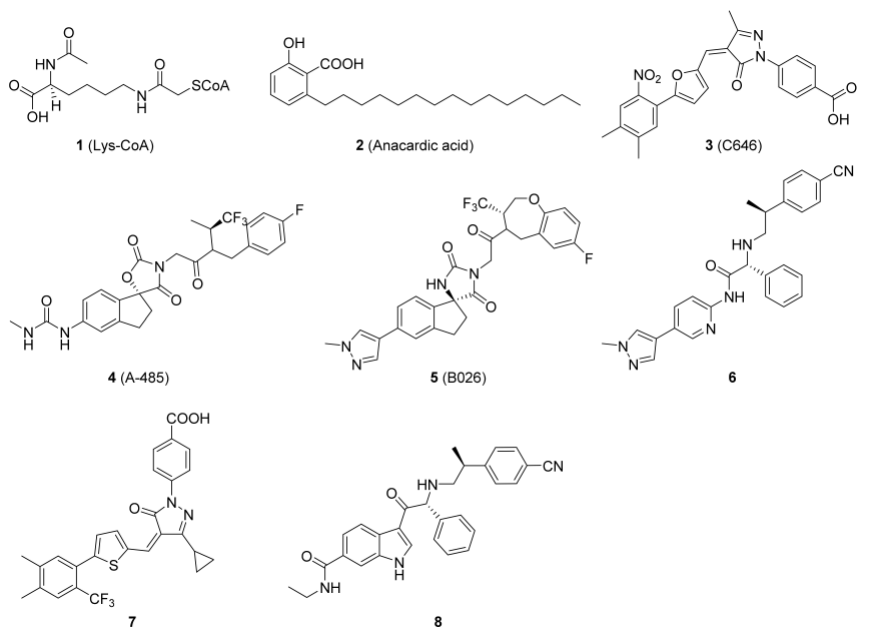
Representative small-molecule p300/CBP HAT inhibitors **1**–**8**.

Early exploration of HAT inhibitors included bisubstrate analogs and natural products, such as Lys-CoA (**1**) and anacardic acid (**2**) (Figure 1), which showed weak potency or poor selectivity [17,18]. Several inhibitors were discovered over time and C646 (**3**) became a commonly used tool molecule [19,20]. In 2017, Abbvie scientists discovered a potent and selective HAT inhibitor, A-485 (**4**), using high-throughput screening in silico and experimental optimization [21]. It competed with acetyl-CoA for the active site of p300, and selectively inhibited the proliferation of haematological and solid cancer cell lines, such as castration-resistant androgen receptor-positive prostate cancer [22,23]. In addition, A-485 also showed a certain positive effect on liver damage and metabolic diseases [24,25]. On the basis of A-485, Zhou et al. identified a preclinical candidate B026 (**5**) with excellent pharmacological properties with the assistance of artificial intelligence and further optimization [26]. In the past decade, several other HAT inhibitors **6**–**8** with excellent potency have been reported [27,28,29,30,31].

**Figure 2:**
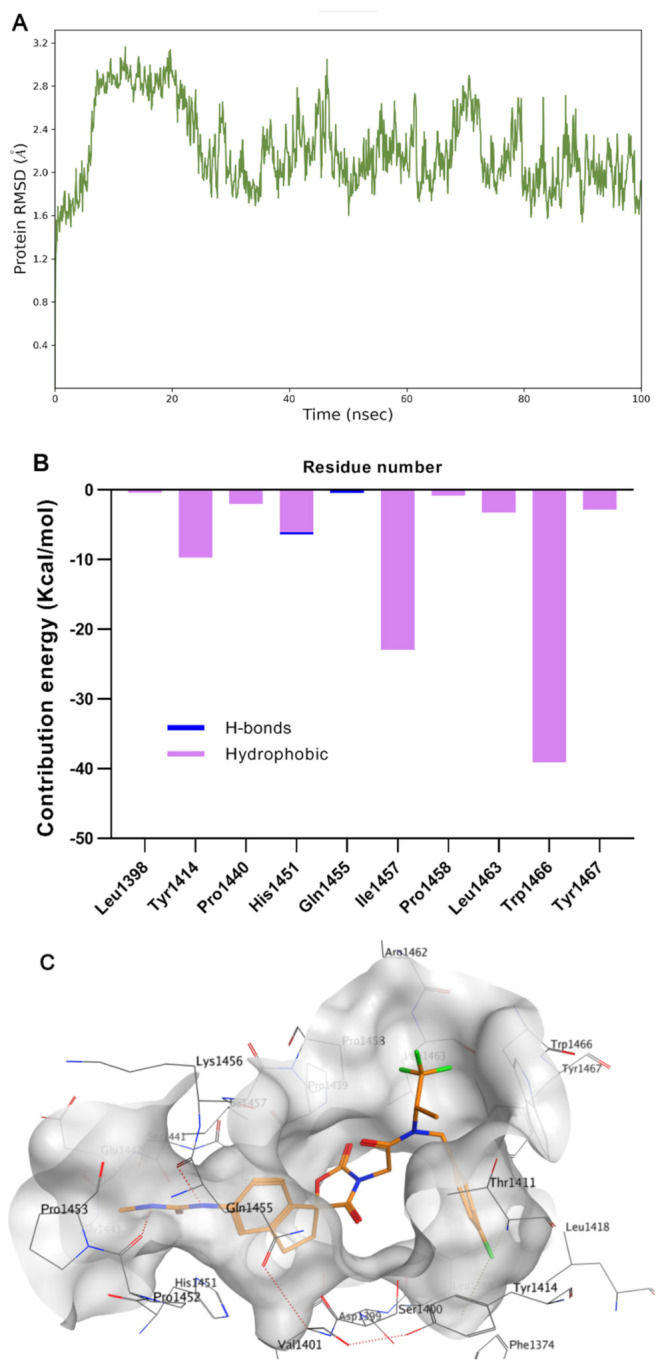
Molecular dynamics simulation and analysis of the interaction of A-485 with p300/CBP HAT domain. (**A**) RMSD of HAT backbone during the simulation time. (**B**) The residue contribution for protein-ligand interaction (blue: hydrogen bonds; purple: hydrophobic contacts). (**C**) Surface and interaction of the complex. (PDB code: 5kj2).

A-485 represents a significant breakthrough, but the cell potency still requires improvement. To better understand the binding mode of A485 and HAT domain, molecular dynamics studies were performed. To evaluate the quantitative contribution energy of the residues in the complex, the most practical MM-PBSA (GBSA) methods are employed [32,33,34]. The RMSD value of the protein backbone was calculated in a trajectory from 0 to 100 ns. As shown in Figure 2A, dynamic equilibrium was attained after approximately 40 ns of simulation. The protein-ligand complex was converged to approximately 1.8 Å. Based on the relatively stable conformation, the two N atoms of methylurea group formed strong hydrogen bonds with Gln1455 and His1451 (Figure 2B,C). In contrast, the hydrogen bond between the carbonyl of spirocyclic lactam and Ser1400 became weaker after dynamics simulation (3.15 Å versus 2.94 Å). The 1,1,1-trifluoroisopropyl moiety was located in the lipophilic pocket formed by crucial Trp1466, together with Thr1411 and Pro1458. A large hydrophobic pocket formed by Val1401, His1451 and Pro1440 was also observed near the indenyl group. These two pockets offer sufficient imaginable space for molecular design.

**Figure 3:**
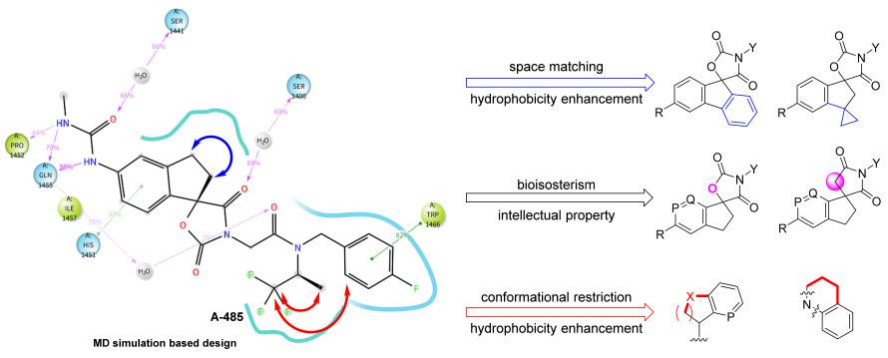
Current main design of new spirocyclic derivatives (P, Q = CH, N).

Therefore, we designed several series of new compounds (Figure 3). First, phenyl or cyclopropyl groups were installed onto the indenyl to match the space and strengthen hydrophobicity. Second, a bioisosterism strategy was utilized in the spirocyclic core structure to acquire intellectual property, namely turning the carbamate into a lactam and the benzene into a pyridine. Finally, in consideration of the important hydrophobicity of Trp1466, we hypothesized that cyclization of the 1,1,1-trifluoroisopropyl and/or with the fluorobenzyl group based on conformational restriction strategy might benefit the affinity. The side chain of R group was expected to regulate the physicochemical properties.

## 2. Results and Discussion

### 2.1. Chemistry

**Scheme 1:**
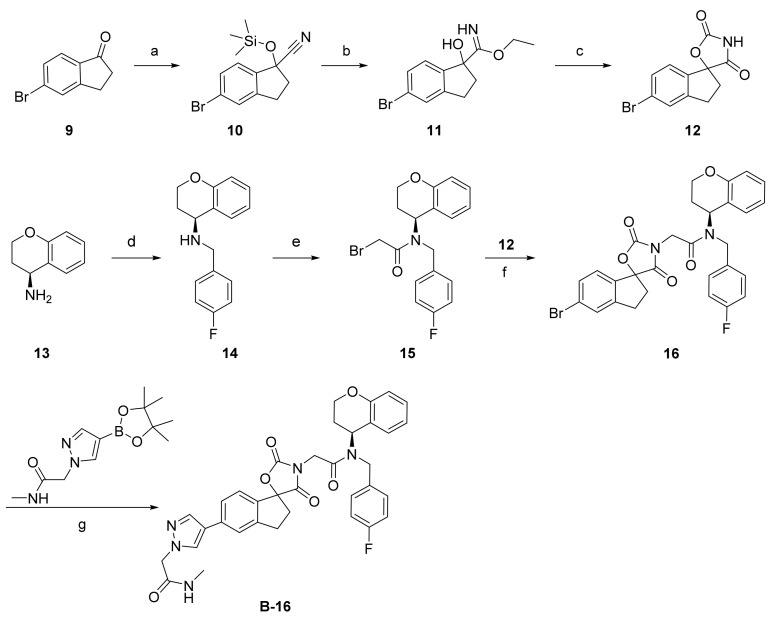
*Reagents and conditions*: (**a**) TMSCN, NMO, acetonitrile, 60 °C, 81%; (**b**) AcCl, EtOH, 76%; (**c**) 1-(bromomethyl)-4-fluorobenzene, trifluoroacetic acid, triphosgene, THF, 10 °C, 93%; (**d**) K_2_CO_3_, DMF, 30 °C, 47%; (**e**) bromoacetyl bromide, methylene chloride, 39%; (**f**) K_2_CO_3_, DMF, 42%; (**g**) Pd(dppf)Cl_2_, NaHCO_3_, 1,4-dioxane, 85 °C, 33%.

As shown in Scheme 1, a series of spirocyclic chromane derivatives were prepared by similar methods, exemplified by **B16**. The remaining synthetic routes are detailed in the Supplementary Material. Starting from commercially available indanone, treatment with trimethylsilyl cyanide and *N*-methylmorpholine *N*-oxide afforded 5-bromo-1-((trimethylsilyl)oxy)-2,3-dihydro-1*H*-indene-1-carbonitrile (**10**). Treatment with acetyl chloride produced compound **11**, which was cyclized with triphosgene and triethylamine to afford core structure **12**. Reaction of (*S*)-chroman-4-amine (**13**) with 4-fluorobenzyl bromide gave **14**, then acylation with bromoacetyl bromide afforded amide **15**. Finally, nucleophilic substitution of this α-bromoamide gave **16**, and Pd-catalyzed Suzuki coupling with a pyrazolyl borate ester afforded **B-16**.

### 2.2. In Vitro Antiproliferative Activities: Structure-Activity Relationship Study

22Rv1 cells was chosen to evaluate the bioactivity in vitro, due to its AR-dependence but proliferation in the absence of androgens, and expression of a high level of AR-V7 variants. It was also representative of enzalutamide-resistant castration-resistant prostate cancer and unmet medical need furthermore [35,36]. As shown in Table 1, introducing a phenyl on the indenyl ring proved to be unsatisfactory, given the loss of antiproliferation activity in **A1**, indicating that this cavity is not large enough to accommodate the benzene ring. Attachment of smaller rings such as cyclopropyl (compound **A2**) was tolerated, showing comparable antiproliferation activity, but modification of the side chain led to decreased potency (compound **A3**). We also sought to construct an oxa-containing six-membered ring between the methylpyrazole and the benzene ring to restrict conformation (compound **A4**), but potency was lost. It was noteworthy that the dihedral angle between the indenyl and the urea moiety of A-486 was obviously bigger after dynamics simulation (54.9 ° versus 27.6 °), consistent with its lack of activity. Finally, changing the carbamate into a lactam (**A5**, **A6**) or benzene to pyridine (**A7**, **A8**) led to sharp decreases in potency. This was likely due to two subtle interactions, namely edge to face π-π stacking betwen the indenyl and His1451, and hydrogen bond between the carbamate carbonyl and Ser1400, respectively.

To optimize these spirocyclic chromane derivatives further, we turned our attention toward the third series of designs. As shown in Table 2, three substituents R^1^, R^2^, and R^3^ were systematically investigated to further explore the structure-activity relationships. Changing fluorophenyl to 2,2-difluorobenzo[*d*][1,3] dioxole (**B1**) led to 10-fold less potency than A-485. A two bridged ring was also incompatible (compounds **B2**, **B3**), indicating that the size and rigidity of the ring had a significant effect on the potency. When the 1,1,1-trifluoroisopropyl group was displaced by an indenyl, several side chains were tolerated (compounds **B4**–**B7**), which confirmed our hypothesis of their regulatory effect on the physicochemical properties. Then an acetamide pyrazolyl was added to explore the displacement of 1,1,1-trifluoroisopropyl and 4-F-benzyl (**B8**–**B16**). An indenyl 5-position fluorine, 4-position pyridylation, and F-pyridylation of R^3^, all led to a 5-fold increased potency (cf. **B11**, **B12** versus **B10**). The activity was getting stronger along the trend of 4,5,6,7-tetrahydro-1*H*-indazole, 1,2,3,4-tetrahydronaphthalene and chromane (compounds **B14**–**B16**). **B16** exhibited 2-fold more potency than A-485. Combining R^2^ and R^3^ into one group seemed to be another way to get a new series of molecules, so **B17** and **B18** were designed, but the bioactivity was reduced dramatically, likely due to mismatch of the hydrophobic pocket.

### 2.3. Pharmacokinetic Evaluation of Compounds ***B8*** and ***B16***

In order to evaluate the druggability of these newly synthesized chromane derivatives, a pharmacokinetic test was performed on several potent compounds (Table 3). After administering 10 mg/kg orally to mice the half-life of **B8** reached 23.9 h. The lower plasma concentration of **B8** was probably attributable to the much larger volume of distribution (V_ss_ = 13.2 h·ng/mL). Compound **B16** showed an modest clearance rate (Cl = 1.8 L/h/kg), volume of distribution (Vss = 1.0 h·ng/mL) and good oral bioavailability (F = 33.2%). Due to the fact p300/CBP are ubiquitously expressed and involved in modulating immune responses [37], the plasma exposure of epigenetic regulator would be moderately controlled. Thus the pharmacokinetic profile of **B16** met the criteria of druggability.

Considering **B16** was a racemic mixuture, we carried out phsical resolution by chiral HPLC and further investigated by 22Rv1 cell antiproliferation and pharmacokinetics profiles (Table 4). However, both **B16**–**P1** and **B16**–**P2** were identical and comparable to the racemate **B16**. Based on their higher maximal inhibitory efficiency (TOP value, Emax) of cell proliferation and availability, **B16**–**P2** was chosen for further study.

### 2.4. Anti-Tumor Efficacy of Compound ***B16***–***P2*** In Vivo

**Figure 4:**
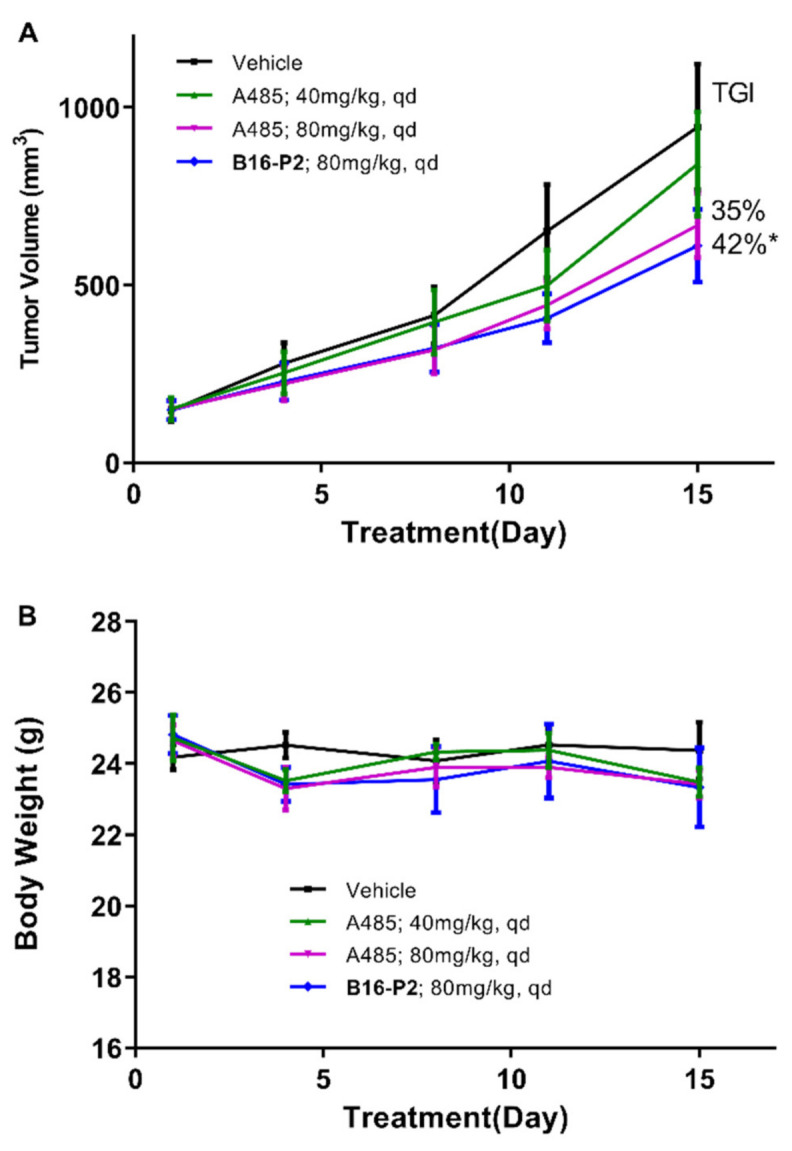
In vivo 22Rv1 xenograft model study of compound **B16-P2** and A-485. (**A**) The tumor growth curve of four groups. (**B**) The body weights of mice over time (*n* = 6, * *p* < 0.05 versus control).

The anti-tumor activity of **B16**–**P2** was then evaluated in vivo, using a 22Rv1 xenograft model (Figure 4). All compounds were administered orally once daily for 15 days, with A-485 as a reference. A statistically significant tumor growth delay was observed in **the B16**–**P2** group (*p* < 0.05). At the same dosage of 80 mg/kg, **B16**–**P2** showed stronger efficacy than A-485 (tumor growth inhibition rate = 42% versus 35%), thus confirming our aforementioned conclusion that the plasma exposure was not a decisive factor. During the study period, **B16**–**P2** was well tolerated, and no death or significant loss of body weight was observed.

### 2.5. Molecular Docking Study of Compound ***B16***

**Figure 5:**
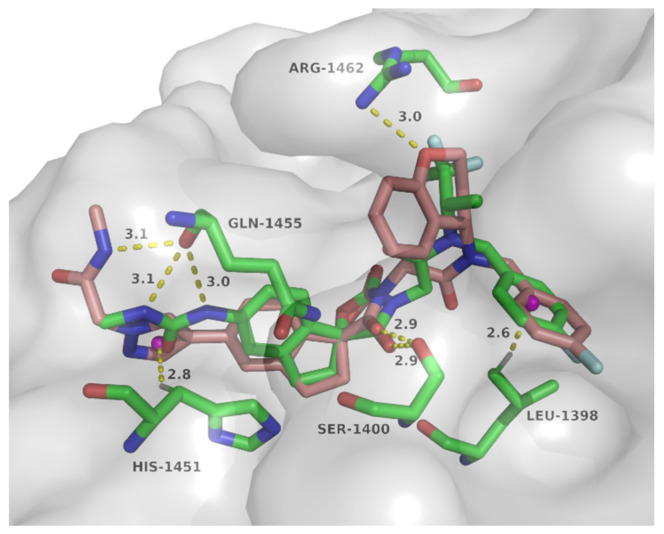
Docking mode of compound **B16** (coppery) with p300 HAT domain overlapping with A-485 (green) (PDB code: 5kj2).

To explore the possible binding mode and rationalize the observed potency of spirocyclic chromane derivative **B16**, molecular docking with p300 HAT domain was performed using a single isomer of spiro chirality (Figure 5). These two structures overlapped well with structural maintenance of two key hydrogen bonds: carbonyl of the oxazolidinedione and Ser1400, C–H…π between 4-F-phenyl and Leu1398. In the side chain region, *N*-methyl-2-(1*H*-pyrazol-1-yl)acetamide of **B16** formed NH…O hydrogen bond with Gln1455 and C–H…π bond with His1451, while two equivalent hydrogen bonds formed between methylurea of A-485 and the carbonyl of Gln1455. Importantly, an additional hydrogen bond was observed between the chromane oxygen of **B16** and Arg1462, which might contribute to the improved activity. Thus this series of spirocyclic chromane derivatives exemplified by **B16** might act as p300 HAT inhibitors.

## 3. Materials and Methods

### 3.1. Chemistry

Generally, unless otherwise specified, the starting materials were purchased commercially and used directly without further purification. All reactions were monitored by thin layer chromatography (TLC) on silica gel plates (HSGF254), and the components were visualized using ultraviolet light or phosphomolybdic acid. Concentration under vacuum means rotary evaporation under reduced pressure at 35–40 °C. Use silica gel (200–300 mesh) for flash column chromatography. The ^1^H-NMR and ^13^C-NMR spectra were collected on a 400 MHz spectrometer (Bruker, MS, USA). HPLC conditions: Column: Xselect CSH C_18_ (4.6 × 150 mm, 3.5 μm), 30 °C; CH_3_CN/H_2_O(containing 0.1% CF_3_COOH) eluent, 10% CH_3_CN (kept for 2 min) to 90% CH_3_CN, gradient 13 min, then kept for 3 min; Flow rate: 1.2 mL/min; Detection: UV 254 nm; unless otherwise stated. 

#### 3.1.1. *N*-(4-Fluorobenzyl)-2-(3-(1-(2-(methylamino)-2-oxoethyl)-1*H*-pyrazol-4-yl)-2′,4′-dioxospiro[fluorene-9,5′-oxazolidin]-3′-yl)-*N*-((*S*)-1,1,1-trifluoropropan-2-yl)acetamide (**A1**)

Prepared using the same procedure as for **A4**, except using 2-(3-bromo-2′,4′-dioxospiro[fluorene-9,5′-oxazolidin]-3′-yl)-*N*-(4-fluorobenzyl)-*N*-((*S*)-1,1,1-trifluoropropan-2-yl)acetamide (76, 0.23 g, 0.39 mmol) instead of **77** and using *N*-methyl-2-(4-(4,4,5,5-tetramethyl-1,3,2-dioxaborolan-2-yl)-1*H*-pyrazol-1-yl) acetamide (0.16 g, 0.58 mmol) instead of 1-methyl-4-(4,4,5,5-tetramethyl-1,3,2-dioxaborolan-2-yl)-1*H*-pyrazole afforded **A1** (0.05 g, 20%). ^1^H-NMR (CDCl_3_) δ 7.93 (s, 1H), 7.78 (s, 1H), 7.73 (s, 1H), 7.69 (d, *J* = 7.4 Hz, 1H), 7.60 (d, *J* = 7.6 Hz, 2H), 7.54 (d, *J* = 7.2 Hz, 1H), 7.51 − 7.46 (m, 1H), 7.45 − 7.39 (m, 1H), 7.39 − 7.33 (m, 1H), 7.29 (s, 2H), 7.10 (t, *J* = 8.1 Hz, 2H), 6.29 (s, 1H), 5.65 − 5.42 (m, 1H), 4.83 (d, *J* = 12.1 Hz, 3H), 4.69 (m, 2H), 4.54 (d, *J* = 16.4 Hz, 1H), 4.32 (d, *J* = 16.4 Hz, 1H), 2.82 (d, *J* = 4.4 Hz, 3H), 1.32 (d, *J* = 7.0 Hz, 3H). ^13^C-NMR (CDCl_3_) δ 171.74, 167.43, 166.75, 162.37 (d, *J* = 245 Hz), 155.06, 142.48, 141.37, 141.19, 138.91, 137.05, 136.96, 135.22, 131.52, 131.35, 129.06, 128.48, 127.26, 127.18, 126.04, 122.15 (q, *J* = 262 Hz), 120.87, 117.89, 116.51, 116.30, 106.73, 90.87, 55.30, 50.69, 50.39, 46.03, 42.35, 26.29, 11.52. HPLC: retention time 12.997 min, 96.51% purity (using an XBridge C_18_ column (4.6 × 150 mm, 3.5 μm) instead of an Xselect CSH C_18_ one (4.6 × 150 mm, 3.5 μm), and ammonia instead of trifluoroacetic acid). HRMS (ESI): *m/z* calcd for C_33_H_27_F_4_N_5_O_5_, 650.2021, found 650.2030 [M + H]^+^.

#### 3.1.2. *N*-(4-Fluorobenzyl)-2-(5′-(3-methylureido)-2′′,4′′-dioxo-2′*H*-dispiro[cyclopropane-1,3′-indene-1′,5′′-oxazolidin]-3′′-yl)-*N*-((*S*)-1,1,1-trifluoropropan-2-yl)acetamide (**A2**)

To a solution of 2-(5′-amino-2′′,4′′-dioxo-2′*H*-dispiro[cyclopropane-1,3′-indene-1′,5′′-oxazolidin]-3′′-yl)-*N*-(4-fluorobenzyl)-*N*-((*S*)-1,1,1-trifluoropropan-2-yl) acetamide (**79**, 0.13 g, 0.26 mmol) and triethylamine (0.10 g, 1.00 mmol) in anhydrous tetrahydrofuran (2 mL) was added triphosgene (0.08 g, 0.26 mmol). The mixture was stirred for 30 min at room temperature. Then methylamine in tetrahydrofuran (2 M, 1.3 mL) was added and the mixture was stirred for 1 h. A saturated solution of sodium bicarbonate was added. The mixture was extracted with ethyl acetate. The organic phase was combined, dried over anhydrous Na_2_SO_4_, and concentrated under vacuum. The residue was purified by silica gel column chromatography (EA:PE = 1:1) to afford **A2** (0.10 g, 69%). ^1^H-NMR (CDCl_3_) δ 7.28 (s, 2H), 7.24 − 7.18 (m, 1H), 7.11 (t, *J* = 8.2 Hz, 2H), 7.03 (d, *J* = 8.7 Hz, 1H), 6.71 (m, 1H), 5.52 − 5.38 (m, 1H), 5.23 (m, 1H), 4.78 − 4.56 (m, 1H), 4.43 (m, 1H), 4.21 (m, 1H), 2.91 − 2.80 (m, 1H), 2.73 (d, *J* = 4.2 Hz, 2H), 2.40 (d, *J* = 14.3 Hz, 1H), 1.32 (d, *J* = 6.8 Hz, 3H), 1.02 (s, 4H). ^13^C-NMR (CDCl_3_) δ 174.40, 167.21, 162.35 (d, *J* = 245 Hz), 156.31, 154.54, 151.60, 142.51, 131.35, 130.34, 127.21, 125.24 (q, *J* = 276 Hz), 124.70, 118.39, 116.51, 116.30, 109.63, 93.65, 46.09, 44.58, 41.80, 40.02, 29.72, 26.74, 25.09, 20.71, 17.57, 11.51. HPLC: retention time 12.360 min, 96.04% purity. HRMS (ESI): *m/z* calcd for C_27_H_26_F_4_N_4_O_5_, 563.1912, found 563.1924 [M + H]^+^.

#### 3.1.3. 2-(5′-(3-Cyclopropylureido)-2′′,4′′-dioxo-2′*H*-dispiro[cyclopropane-1,3′-indene-1′,5′′-oxazolidin]-3′′-yl)-*N*-(4-fluorobenzyl)-*N*-((*S*)-1,1,1-trifluoropropan-2-yl)acetamide (**A3**)

Prepared using the same procedure as for **A2** to afford **A3** (0.078 g, 51%). ^1^H-NMR (CDCl_3_) δ 7.40 − 7.26 (m, 3H), 7.14 (d, *J* = 2.5 Hz, 1H), 7.13 − 7.06 (m, 2H), 7.01 − 6.88 (m, 1H), 5.55 − 5.40 (m, 1H), 5.34 − 5.23 (m, 1H), 4.75 − 4.59 (m, 2H), 4.46 − 4.40 (m, 1H), 4.21 (t, *J* = 15.8 Hz, 1H), 2.89 (dd, *J* = 14.3, 8.7 Hz, 1H), 2.55 (s, 1H), 2.42 (d, *J* = 14.3 Hz, 1H), 1.31 (d, *J* = 6.9 Hz, 3H), 1.08 − 0.99 (m, 4H), 0.78 (d, *J* = 5.5 Hz, 2H), 0.57 (s, 2H). ^13^C-NMR (CDCl_3_) δ 174.14, 167.06, 162.34 (d, *J* = 246 Hz), 156.30, 154.51, 154.43, 151.57, 142.08, 131.41, 130.76, 127.28, 127.20, 124.77, 124.71, 117.23 (q, *J* = 205 Hz), 116.28, 109.54, 93.56, 93.45, 46.04, 44.63, 41.83, 40.34, 25.14, 22.55, 17.67, 14.71, 11.52, 7.36. HPLC: retention time 12.830 min, 97.90% purity. HRMS (ESI): *m/z* calcd for C_29_H_28_F_4_N_4_O_5_, 589.2069, found 589.2081 [M + H]^+^.

#### 3.1.4. *N*-(4-Fluorobenzyl)-2-(2-methyl-2′,4′-dioxo-2,4,8,9-tetrahydrospiro[cyclopenta [6,7]chromeno [3,4-c]pyrazole-7,5′-oxazolidin]-3′-yl)-*N*-((*S*)-1,1,1-trifluoropropan-2-yl)acetamide (**A4**)

Prepared using the same procedure as for **77**, except using 2-methyl-2,4,8,9-tetrahydrospiro cyclopenta [6,7] chromeno [3,4-c]pyrazole-7,5′-oxazolidine]-2′,4′-dione (40, 0.01 g, 0.03 mmol) instead of 28 afforded **A4** (0.003 g, 19%). ^1^H-NMR (CDCl_3_) δ 7.53 (s, 1H), 7.29 (s, 2H), 7.21 (s, 1H), 7.13 (d, *J* = 7.9 Hz, 1H), 7.09 (d, *J* = 8.7 Hz, 1H), 7.05 − 6.93 (m, 1H), 5.61 − 5.45 (m, 1H), 5.22 (s, 2H), 4.73 − 4.63 (m, 2H), 4.43 − 4.39 (m, 1H), 4.24 − 4.19 (m, 1H), 3.93 (s, 3H), 3.16 (s, 1H), 3.11 − 2.98 (m, 1H), 2.87 − 2.73 (m, 1H), 2.60 − 2.48 (m, 1H), 1.30 (d, *J* = 3.0 Hz, 3H). ^13^C-NMR (CDCl_3_) δ 166.73, 162.37 (d, *J* = 243 Hz), 156.69, 152.01, 145.33, 139.82, 138.46, 130.92, 128.86, 127.27, 124.60, 123.22, 121.59, 120.66 (q, *J* = 287 Hz), 119.06, 116.50, 116.28, 113.16, 112.95, 94.89, 64.37, 46.01, 39.09, 35.62, 29.71, 29.38, 22.70, 14.13. HPLC: retention time 13.194 min, 95.56% purity. HRMS (ESI): *m/z* calcd for C_28_H_24_F_4_N_4_O_5_, 573.1753, found 573.1763 [M + H]^+^.

#### 3.1.5. *N*-(4-Fluorobenzyl)-2-(3-(1-(2-(methylamino)-2-oxoethyl)-1*H*-pyrazol-4-yl)-2′,4′-dioxo-5,6-dihydrospiro[cyclopenta[b]pyridine-7,5′-oxazolidin]-3′-yl)-*N*-((*S*)-1,1,1-trifluoropropan-2-yl)acetamide (**A5**)

A solution of 2-(3-bromo-2′,4′-dioxo-5,6-dihydrospiro[cyclopenta[*b*]pyridine-7,5′-oxazolidin]-3′-yl)-*N*-(4-fluorobenzyl)-*N*-((S)-1,1,1-trifluoropropan-2-yl) acetamide (**80**, 0.27 g, 0.50 mmol), 1-methyl-4-(4,4,5,5-tetra-methyl-1,3,2-dioxaborolan-2-yl)-1*H*-pyrazole (0.20 g, 0.75 mmol), and Pd(dppf)Cl_2_ (0.04 g, 0.05 mmol) in a saturated solution of potassium bicarbonate (0.5 mL) and 1.4-dioxane (5 mL) was stirred in 85 °C under nitrogen until completion. The mixture was cooled to room temperature and concentrated under vacuum. The residue was purified by silica gel column chromatography (EA:PE = 1:4) to afford **A5** (0.07 g, 24%). ^1^H-NMR (400 MHz, CDCl_3_) δ 8.70 (d, *J* = 6.0 Hz, 1H), 8.09 (s, 1H), 8.03 (s, 1H), 7.44 (s, 1H), 7.29 (s, 2H), 7.18 − 7.09 (m, 2H), 6.23 (s, 1H), 5.57 − 5.41 (m, 1H), 4.85 (s, 2H), 4.74 − 4.63 (m, 2H), 4.48 − 4.40 (m, 1H), 4.33 − 4.28 (m, 1H), 3.35 − 3.22 (m, 1H), 3.22 − 3.12 (m, 1H), 2.86 − 2.85 (m, 1H), 2.81 (d, *J* = 4.5 Hz, 4H), 2.65 − 2.57 (m, 1H), 1.33 (s, 3H). ^13^C-NMR (101 MHz, CDCl_3_) δ 167.32, 167.07, 162.36 (d, *J* = 218 Hz), 155.28, 152.92, 146.59, 139.72, 131.37, 130.96, 130.47, 128.05 (q, *J* = 161 Hz), 127.62, 124.98, 124.24, 116.55, 116.34, 115.76, 92.85, 63.41, 57.41, 55.35, 46.05, 42.03, 35.37, 30.27, 26.30, 19.21, 13.77. HPLC: retention time 11.383 min, 98.74% purity. HRMS (ESI): *m/z* calcd for C_28_H_26_F_4_N_6_O_5_, 603.1974, found 603.1983 [M + H]^+^.

#### 3.1.6. *N*-(4-Fluorobenzyl)-2-(3-(1-methyl-1*H*-pyrazol-4-yl)-2′,4′-dioxo-5,6-dihydrospiro [cyclopenta[c]pyridine-7,5′-oxazolidin]-3′-yl)-*N*-((*S*)-1,1,1-trifluoropropan-2-yl)acetamide (**A6**)

Prepared using the same procedure as for **77** (see Supplementary Materials for details), except using 3-(1-methyl-1*H*-pyrazol-4-yl)-5,6-dihydrospiro[cyclopenta[*c*]pyridine-7,5′-oxazolidine]-2′,4′-dione (**53**, 0.12 g, 0.42 mmol) instead of **76** afforded **A6** (0.06 g, 24%). ^1^H-NMR (CDCl_3_) δ 8.66 (d, *J* = 7.7 Hz, 1H), 7.94 (s, 2H), 7.54 (s, 1H), 7.42 (s, 2H), 7.29 (s, 1H), 7.12 (t, *J* = 7.9 Hz, 2H), 5.62 − 5.38 (m, 1H), 4.76 − 4.59 (m, 2H), 4.46 − 4.42 (m, 1H), 4.26 − 4.20 (m, 1H), 3.95 (s, 3H), 3.31 − 3.20 (m, 1H), 3.19 − 3.10 (m, 1H), 2.86 − 2.76 (m, 1H), 2.59 (s, 1H), 1.32 (s, 3H). ^13^C-NMR (CDCl_3_) δ 173.53, 166.66, 162.38 (d, *J* = 245 Hz), 155.08, 153.82, 146.39, 137.73, 136.89, 131.30, 131.27, 129.56, 127.27, 127.19, 124.95 (q, *J* = 313 Hz), 116.52, 116.31, 115.41, 114.19, 92.98, 46.03, 41.96, 39.24, 38.95, 35.41, 30.21, 11.52. HPLC: retention time 11.922 min, 95.80% purity. HRMS (ESI): *m/z* calcd for C_26_H_23_F_4_N_5_O_4_, 546.1759, found 546.1768 [M + H]^+^.

#### 3.1.7. *N*-(4-Fluorobenzyl)-2-(5-(1-(2-(methylamino)-2-oxoethyl)-1*H*-pyrazol-4-yl)-2′,5′-dioxo-2,3-dihydrospiro[indene-1,3′-pyrrolidin]-1′-yl)-*N*-((*S*)-1,1,1-trifluoropropan-2-yl)acetamide (**A7**)

Prepared using the same procedure as for **A5**, except using 2-(5-bromo-2′,5′-dioxo-2,3-dihydrospiro[indene-1,3′-pyrrolidin]-1′-yl)-*N*-(4-fluorobenzyl)-*N*-((*S*)-1,1,1-trifluoropropan-2-yl)acetamide (**82**, 0.10 g, 0.19 mmol) instead of **77** afforded **A7** (0.02 g, 18%). ^1^H-NMR (CDCl_3_) δ 7.85 (s, 1H), 7.67 (s, 1H), 7.39 (s, 1H), 7.37 − 7.33 (m, 1H), 7.32 − 7.28 (m, 2H), 7.22 (d, *J* = 7.9 Hz, 1H), 7.10 (t, *J* = 8.3 Hz, 2H), 6.26 (s, 1H), 5.54 − 5.38 (m, 1H), 4.83 (s, 2H), 4.74 − 4.61 (m, 2H), 4.44 − 4.40 (m, 1H), 4.18 − 4.12(m, 1H), 3.29 − 3.20 (m, 1H), 3.09 (d, *J* = 6.5 Hz, 1H), 3.05 (d, *J* = 2.6 Hz, 1H), 3.01 (s, 1H), 2.81 (d, *J* = 4.5 Hz, 3H), 2.26 − 2.17 (m, 1H), 1.66 (m, 1H), 1.34 − 1.27 (m, 3H). ^13^C-NMR (CDCl_3_) δ 180.38, 175.31, 167.64, 167.30, 162.34 (d, *J* = 245 Hz), 144.97, 138.88, 132.04, 128.04, 127.31, 127.23, 125.16, 125.11, 123.68 (q, *J* = 297 Hz), 123.27, 123.08, 122.19, 122.12, 116.39, 116.17, 55.47, 55.24, 46.10, 43.44, 40.68, 38.02, 31.35, 29.72, 26.28, 11.57. HPLC: retention time 11.664 min, 98.61% purity. HRMS (ESI): *m/z* calcd for C_30_H_29_F_4_N_5_O_4_, 600.2228, found 600.2240 [M + H]^+^.

#### 3.1.8. *N*-(4-Fluorobenzyl)-2-(5-(3-methylureido)-2′,5′-dioxo-2,3-dihydrospiro[indene-1,3′-pyrrolidin]-1′-yl)-*N*-((*S*)-1,1,1-trifluoropropan-2-yl)acetamide (**A8**)

Prepared using the same procedure as for **A2**, except using 2-(5-amino-2′,5′-dioxo-2,3-dihydrospiro[indene-1,3′-pyrrolidin]-1′-yl)-*N*-(4-fluorobenzyl)-*N*-((*S*)-1,1,1-trifluoro-propan-2-yl)acetamide (**84**, 0.04 g, 0.09 mmol) instead of **79** afforded **A8** (0.03g, 58%). ^1.^ H-NMR (CDCl_3_) δ 7.35 − 7.27 (m, 2H), 7.14 − 6.99 (m, 4H), 6.82 (d, *J* = 8.0 Hz, 1H), 5.41 (dd, *J* = 12.9, 6.6 Hz, 1H), 5.23 (d, *J* = 4.2 Hz, 1H), 4.74 − 4.60 (m, 2H), 4.41 − 4.37 (m, 1H), 4.16 − 4.10 (m, 1H), 3.09 (d, *J* = 8.3 Hz, 1H), 2.93 (s, 2H), 2.74 (d, *J* = 4.0 Hz, 3H), 2.12 (dd, *J* = 12.8, 6.8 Hz, 1H), 1.77 (s, 1H), 1.27 (dd, *J* = 10.1, 7.8 Hz, 3H). ^13^C-NMR (CDCl_3_) δ 181.10, 175.50, 167.61, 162.29 (d, *J* = 245 Hz), 156.71, 145.14, 139.47, 137.98, 137.87, 131.58, 127.30, 127.22, 122.85, 122.74, 117.97 (q, *J* = 269 Hz), 116.39, 116.17, 55.20, 50.19, 46.15, 43.36, 40.66, 38.10, 31.39, 26.77, 11.49. HPLC: retention time 11.512 min, 93.15% purity. HRMS (ESI): *m/z* calcd for C_26_H_26_F_4_N_4_O_4_, 535.1963, found 535.1975 [M + H]^+^.

#### 3.1.9. *N*-((2,2-Difluorobenzo[d][1,3]dioxol-5-yl)methyl)-2-((*R*)-5-(1-methyl-1*H*-pyrazol-4-yl)-2′,4′-dioxo-2,3-dihydrospiro[indene-1,5′-oxazolidin]-3′-yl)-*N*-((*S*)-1,1,1-trifluoropropan-2-yl)acetamide (**B1**)

To a solution of 2-((*S*)-5-bromo-2′,4′-dioxo-2,3-dihydrospiro[indene-1,5′-oxazolidin]-3′-yl)-N-((2,2-difluorobenzo[*d*][1,3]dioxol-5-yl)methyl)-*N*-((*S*)-1,1,1-trifluoropropan-2-yl)acetamide (**85**, 0.26 g, 0.43 mmol), 1-methyl-4-(4,4,5,5-tetramethyl-1,3,2-dioxaborolan-2-yl)-1*H*-pyrazole (0.099 g, 0.48 mmol) and Pd(dppf)Cl_2_ (0.031 g, 0.043 mmol) in 1,4-dioxane (5 mL) was added a saturated solution of sodium bicarbonate (0.5 mL) under nitrogen. The mixture was stirred in 85 °C until completion, cooled to room temperature, concentrated, and purified directly by silica gel column chromatography to afford **B1** (0.068 g, 26%). ^1^H-NMR (CDCl_3_) δ 7.75 (s, 1H), 7.61 (s, 1H), 7.51 − 7.43 (m, 1H), 7.43 − 7.37 (m, 2H), 7.14 − 7.08 (m, 1H), 7.06 (s, 1H), 6.97 − 6.90 (m, 1H), 5.63 − 5.40 (m, 1H), 4.74 − 4.62 (m, 2H), 4.47 − 4.39 (m, 1H), 4.26 − 4.20 (m, 1H), 3.94 (s, 3H), 3.31 − 3.20 (m, 1H), 3.19 − 3.08 (m, 1H), 2.85 − 2.74 (m, 1H), 2.63 − 2.50 (m, 1H), 1.32 (s, 3H). ^13^C-NMR (CDCl_3_) δ 174.26, 174.10, 166.87, 154.37, 146.02, 144.60, 143.44, 136.96, 135.61, 134.90, 131.99, 131.67, 127.35, 125.29, 124.85, 123.72 (q, *J* = 214 Hz), 121.84, 120.66, 110.18, 107.13, 94.82, 50.61, 46.21, 41.66, 39.18, 35.40, 30.27, 11.59. HPLC: retention time 13.762 min, 96.37% purity. HRMS (ESI): *m/z* calcd for C_28_H_23_F_5_N_4_O_6_, 607.1611, found 607.1627 [M + H]^+^.

#### 3.1.10. *N*-(4-Fluorobenzyl)-2-((*R*)-5-(1-methyl-1*H*-pyrazol-4-yl)-2′,4′-dioxo-2,3-dihydrospiro[indene-1,5′-oxazolidin]-3′-yl)-*N*-(8-methyl-8-azabicyclo[3.2.1]octan-3-yl)acetamide (**B2**)

Prepared using the same procedure as for **B1**, except using 2-((*R*)-5-bromo-2′,4′-dioxo-2,3-dihydrospiro[indene-1,5′-oxazolidin]-3′-yl)-N-(4-fluorobenzyl)-N-(8-methyl-8-azabicyclo[3.2.1]octan-3-yl)acetamide (**86**, 0.089 g, 0.16 mmol) instead of **85** to afford **B2** (0.015 g, 17%). ^1^H-NMR (CDCl_3_) δ 7.79 − 7.72 (m, 1H), 7.64 − 7.58 (m, 1H), 7.54 (d, *J* = 7.9 Hz, 1H), 7.48 − 7.35 (m, 3H), 7.15 − 7.07 (m, 2H), 7.02 − 6.95 (m, 1H), 4.67 (s, 1H), 4.54 − 4.40 (m, 3H), 4.33 (s, 1H), 4.01 − 3.87 (m, 3H), 3.54 (s, 1H), 3.31 − 3.24 (m, 1H), 3.19 − 3.13 (m, 1H), 2.86 − 2.79 (m, 1H), 2.64 − 2.51 (m, 3H), 2.29 − 2.17 (m, 3H), 2.01 (d, *J* = 5.2 Hz, 1H), 1.26 (s, 4H), 0.88 (s, 2H). HPLC: retention time 13.183 min, 97.55% purity. HRMS (ESI): *m/z* calcd for C_32_H_34_FN_5_O_4_, 572.2668, found 572.2687 [M + H]^+^.

#### 3.1.11. *N*-(4-Fluorobenzyl)-2-(5-(1-methyl-1*H*-pyrazol-4-yl)-2′,4′-dioxo-2,3-dihydrospiro [indene-1,5′-oxazolidin]-3′-yl)-*N*-((*S*)-quinuclidin-3-yl)acetamide (**B3**)

To a solution of 2-(5-(1-methyl-1*H*-pyrazol-4-yl)-2′,4′-dioxo-2,3-dihydrospiro[indene-1,5′-oxazolidin]-3′-yl)-*N*-((*S*)-quinuclidin-3-yl)acetamide (0.12 g, 0.26 mmol) and 1-(bromomethyl)-4-fluorobenzene (0.14 g, 0.78 mmol) in DMF (2 mL) was added K_2_CO_3_ (0.14 g, 1 mmol). The mixture was stirred at room temperature overnight. After completion, water (10 mL) was added. The mixture was extracted with ethyl acetate. The organic phase was combined, dried over anhydrous Na_2_SO_4_, and concentrated under vacuum. The residue was purified by silica gel column chromatography (MeOH:DCM = 10:1) to afford **B3** (0.50 g, 34%). ^1^H-NMR (400 MHz, CDCl_3_) δ 8.82 − 8.62 (m, 1H), 7.71 (s, 1H), 7.60 (s, 1H), 7.44 − 7.38 (m, 3H), 7.34 (d, *J* = 5.0 Hz, 1H), 7.08 (s, 2H), 4.50 − 4.39 (m, 4H), 3.91 (s, 3H), 3.28 − 3.18 (m, 2H), 3.15 − 3.06 (m, 2H), 2.95 (s, 2H), 2.84 − 2.74 (m, 2H), 2.62 − 2.29 (m, 4H), 1.95 − 1.76 (m, 4H). ^13^C-NMR (101 MHz, CDCl_3_) δ 174.43, 166.10, 162.36 (d, *J* = 245 Hz), 154.96, 146.03, 136.87, 135.54, 135.31, 135.10, 127.41, 125.23, 124.74, 122.53, 122.17, 121.76, 116.75, 116.53, 115.90, 115.68, 115.53, 94.52, 52.89, 45.45, 41.99, 39.15, 38.62, 35.20, 30.29, 24.97, 22.50, 18.75. HPLC: retention time 8.273 min, 95.59% purity. HRMS (ESI): *m/z* calcd for C_31_H_32_FN_5_O_4_, 558.2511, found 558.2522 [M + H]^+^.

#### 3.1.12. *N*-((S)-2,3-Dihydro-1*H*-inden-1-yl)-N-(4-fluorobenzyl)-2-(5-(1-methyl-1*H*-pyrazol-4-yl)-2′,4′-dioxo-2,3-dihydrospiro[indene-1,5′-oxazolidin]-3′-yl)acetamide (**B4**)

To a solution of 2-(5-bromo-2′,4′-dioxo-2,3-dihydrospiro[indene-1,5′-oxazolidin]-3′-yl)-*N*-((*S*)-2,3-dihydro-1*H*-inden-1-yl)-*N*-(4-fluorobenzyl)acetamide (**88**, 0.10 g, 0.18 mmol), 1-methyl-4-(4,4,5,5-tetramethyl-1,3,2-dioxaborolan-2-yl)-1*H*-pyrazole (0.04 g, 0.20 mmol) and Pd(dppf)Cl_2_ (0.013 g, 0.018 mmol) in 1,4-dioxane (5 mL) was added a saturated solution of sodium bicarbonate (0.5 mL) under nitrogen. The mixture was stirred in 85 °C until completion. The mixture was cooled to room temperature, quenched with water (10 mL), and extracted with ethyl acetate. The organic phase was combined, dried over anhydrous Na_2_SO_4_, and concentrated under vacuum. The residue was purified by silica gel column chromatography (DCM: MeOH = 20:1) to afford **B4** (0.080 g, 80%). ^1^H-NMR (CDCl_3_) δ 7.75 (s, 1H), 7.61 (s, 1H), 7.58 − 7.51 (m, 1H), 7.45 − 7.36 (m, 2H), 7.29 (s, 1H), 7.25 − 7.18 (m, 4H), 7.10 − 6.99 (m, 2H), 6.97 − 6.88 (m, 1H), 5.61 − 5.30 (m, 1H), 4.73 − 4.55 (m, 1H), 4.53 − 4.43 (m, 1H), 4.28 − 4.17 (m, 1H), 4.10 − 4.01 (m, 1H), 3.94 (s, 3H), 3.35 − 3.21 (m, 1H), 3.22 − 3.08 (m, 1H), 2.82 (d, *J* = 6.3 Hz, 2H), 2.62 − 2.57 (m, 1H), 2.49 − 2.36 (m, 1H), 2.09 − 1.91 (m, 1H), 1.91 − 1.69 (m, 1H). ^13^C-NMR (CDCl_3_) δ 174.45, 166.11, 162.20 (d, *J* = 245 Hz), 154.73, 146.02, 143.92, 143.34, 140.43, 136.98, 135.51, 135.15, 128.84, 128.29, 127.34, 127.23, 126.91, 125.34, 125.04, 124.33, 122.73, 121.81, 116.19, 115.40, 115.18, 94.66, 60.60, 46.20, 41.83, 39.19, 35.45, 31.14, 30.32, 30.14. HPLC: retention time 16.846 min, 98.62% purity.HRMS (ESI): *m/z* calcd for C_33_H_29_FN_4_O_4_, 565.2249, found 565.2258 [M + H]^+^.

#### 3.1.13. *N*-((*S*)-2,3-Dihydro-1*H*-inden-1-yl)-*N*-(4-fluorobenzyl)-2-(5-(3-methylureido)-2′,4′-dioxo-2,3-dihydrospiro[indene-1,5′-oxazolidin]-3′-yl)acetamide (**B5**)

Prepared using with the same procedure as for **A3**, except using 2-(5-amino-2′,5′-dioxo-2,3-dihydrospiro[indene-1,3′-pyrrolidin]-1′-yl)-*N*-((*S*)-2,3-dihydro-1*H*-inden-1-yl)-*N*-(4-fluorobenzyl)acetamide (**90**, 0.11 g, 0.22 mmol) instead of **79** afforded **B5** (0.061 g, 50%). ^1^H-NMR (CDCl_3_) δ 7.56 − 7.45 (m, 1H), 7.33 − 7.28 (m, 2H), 7.23 − 7.16 (m, 3H), 7.12 (d, *J* = 7.3 Hz, 1H), 7.06 (d, *J* = 7.8 Hz, 1H), 6.99 (d, *J* = 3.7 Hz, 1H), 6.94 − 6.78 (m, 2H), 5.42 (t, *J* = 7.0 Hz, 1H), 5.25 (d, *J* = 5.2 Hz, 1H), 4.81 (dd, *J* = 15.5, 10.5 Hz, 1H), 4.71 − 4.56 (m, 1H), 4.54 − 4.41 (m, 1H), 4.24 (q, *J* = 11.6 Hz, 1H), 4.11 − 3.97 (m, 1H), 3.17 − 3.06 (m, 1H), 3.06 − 2.95 (m, 1H), 2.79 (dd, *J* = 13.0, 6.6 Hz, 2H), 2.72 (d, *J* = 3.3 Hz, 3H), 2.60 − 2.49 (m, 1H), 2.46 − 2.33 (m, 1H), 2.08 − 1.92 (m, 1H), 1.77 − 1.60 (m, 1H). ^13^C-NMR (CDCl_3_) δ 174.70, 166.38, 165.66, 162.22 (d, *J* = 246 Hz), 156.31, 154.85, 146.76, 142.13, 128.93, 128.37, 127.38, 126.93, 125.41, 125.09, 124.77, 124.20, 123.51, 118.68, 116.00, 115.44, 95.05, 95.00, 60.93, 46.46, 41.83, 35.52, 31.94, 31.64, 30.37, 29.71, 26.74. HPLC: retention time 15.446 min, 99.17% purity. HRMS (ESI): *m/z* calcd for C_31_H_29_FN_4_O_5_, 557.2195, found 557.2210 [M + H]^+^.

#### 3.1.14. *N*-((*S*)-2,3-Dihydro-1*H*-inden-1-yl)-*N*-(4-fluorobenzyl)-2-(5-(1-(2-hydroxy-2-methylpropyl)-1*H*-pyrazol-4-yl)-2′,4′-dioxo-2,3-dihydrospiro[indene-1,5′-oxazolidin]-3′-yl)acetamide (**B6**)

To a solution of 2-(5-bromo-2′,4′-dioxo-2,3-dihydrospiro[indene-1,5′-oxazolidin]-3′-yl)-*N*-((*S*)-2,3-dihydro-1*H*-inden-1-yl)-*N*-(4-fluorobenzyl) acetamide (**88**, 0.10 g, 0.18 mmol), 2-methyl-1-(4-(4,4,5,5-tetramethyl-1,3,2-dioxaborolan-2-yl)-1*H*-pyrazol-1-yl)propan-2-ol (0.052 g, 0.20 mmol) and Pd(dppf)Cl_2_ (0.013 g, 0.018 mmol) in 1,4-dioxane (5 mL) was added a saturated solution of sodium bicarbonate (0.5 mL) under nitrogen. The mixture was stirred in 85 °C until completion, quenched with water (10 mL), and extracted with ethyl acetate. The organic phase was combined, dried over anhydrous Na_2_SO_4_, and concentrated under vacuum. The residue was purified by silica gel column chromatography (DCM: MeOH = 20:1) to afford **B6** (0.061 g, 55%). ^1^H-NMR (CDCl_3_) δ 7.81 (s, 1H), 7.68 (s, 1H), 7.55 (t, *J* = 8.6 Hz, 1H), 7.46 − 7.39 (m, 2H), 7.30 (d, *J* = 8.1 Hz, 1H), 7.24 − 7.17 (m, 4H), 7.08 − 7.02 (m, 2H), 6.95 − 6.90 (m, 1H), 5.40 (t, *J* = 6.6 Hz, 1H), 4.87 (dd, *J* = 15.5, 9.4 Hz, 1H), 4.72 − 4.55 (m, 1H), 4.49 (d, *J* = 5.3 Hz, 1H), 4.25 − 4.20 (m, 1H), 4.09 (s, 2H), 3.70 (s, 1H), 3.31 − 3.25 (m, 1H), 3.18 − 3.13 (m, 1H), 2.88 − 2.80 (m, 3H), 2.66 − 2.58 (m, 1H), 2.44 − 2.36 (m, 1H), 1.20 (s, 6H). ^13^C-NMR (CDCl_3_) δ 174.38, 166.10, 162.20 (d, *J* = 245 Hz), 154.67, 146.04, 140.43, 137.43, 135.40, 135.17, 128.84, 128.26, 127.41, 127.33, 127.22, 126.94, 125.38, 125.20, 125.04, 124.34, 122.28, 121.89, 116.18, 115.97, 115.38, 115.16, 94.60, 70.74, 62.09, 46.23, 41.84, 35.44, 31.13, 30.31, 30.14, 29.71, 26.88. HPLC: retention time 16.483 min, 99.46% purity. HRMS (ESI): *m/z* calcd for C_36_H_35_FN_4_O_5_, 623.2664, found 623.2663 [M + H]^+^.

#### 3.1.15. *N*-((S)-2,3-Dihydro-1*H*-inden-1-yl)-*N*-(4-fluorobenzyl)-2-(5-(1-(2-(methylamino)-2-oxoethyl)-1*H*-pyrazol-4-yl)-2′,4′-dioxo-2,3-dihydrospiro[indene-1,5′-oxazolidin]-3′-yl)acetamide (**B7**)

Prepared using the same procedure as for **B6**, except using *N*-methyl-2-(4-(4,4,5,5-tetramethyl-1,3,2-dioxaborolan-2-yl)-1*H*-pyrazol-1-yl) acetamide (0.072 g, 0.27 mmol) instead of 2-methyl-1-(4-(4,4,5,5-tetramethyl-1,3,2-dioxaborolan-2-yl)-1*H*-pyrazol-1-yl)propan-2-ol afforded **B7** (0.07 g, 43%). ^1^H-NMR (CDCl_3_) δ 7.88 (s, 1H), 7.71 (s, 1H), 7.61 − 7.55 (m, 1H), 7.42 (d, *J* = 14.3 Hz, 3H), 7.22 (d, *J* = 7.5 Hz, 4H), 7.10 − 7.01 (m, 2H), 6.93 (s, 1H), 4.91 − 4.87 (m, 1H), 4.83 (s, 2H), 4.72 − 4.56 (m, 1H), 4.52 − 4.46 (m, 1H), 4.29 − 4.18 (m, 1H), 4.13 − 4.02 (m, 1H), 3.29 (dd, *J* = 14.9, 6.5 Hz, 1H), 3.17 (dd, *J* = 11.2, 7.6 Hz, 1H), 2.97 − 2.82 (m, 4H), 2.81 (d, *J* = 4.5 Hz, 3H), 2.62 (dd, *J* = 9.3, 4.6 Hz, 1H), 2.41 (d, *J* = 5.8 Hz, 1H). ^13^C-NMR (CDCl_3_) δ 174.34, 167.49, 166.10, 162.23 (d, *J* = 250 Hz), 154.64, 146.15, 138.97, 135.86, 134.51, 128.86, 128.32, 127.40, 127.22, 126.95, 125.49, 125.38, 125.22, 125.03, 124.32, 123.67, 122.03, 116.19, 115.98, 115.39, 115.19, 94.49, 62.74, 55.28, 46.23, 41.86, 35.44, 30.31, 30.14, 29.71, 26.27. HPLC: retention time 15.455 min, 99.43% purity. HRMS (ESI): *m/z* calcd for C_35_H_32_FN_5_O_5_, 622.2460, found 622.2476 [M + H]^+^.

#### 3.1.16. *N*-((*S*)-6,7-Dihydro-5H-cyclopenta[b]pyridin-7-yl)-*N*-(4-fluorobenzyl)-2-(5-(1-(2-(methylamino)-2-oxoethyl)-1*H*-pyrazol-4-yl)-2′,4′-dioxo-2,3-dihydrospiro[indene-1,5′-oxazolidin]-3′-yl)acetamide (**B8**)

Prepared using the same procedure as for **B4**, except using 2-(5-bromo-2′,4′-dioxo-2,3-dihydrospiro[indene-1,5′-oxazolidin]-3′-yl)-*N*-((*S*)-6,7-dihydro-5*H*-cyclopenta[b]pyridin-7-yl)-*N*-(4-fluorobenzyl)acetamide (**91**, 0.078 g, 0.14 mmol) instead of **88** afforded **B8** (0.02 g, 23%). ^1^H-NMR (CDCl_3_) δ 8.47 (s, 1H), 7.88 (s, 1H), 7.71 (s, 1H), 7.63 − 7.49 (m, 2H), 7.46 − 7.37 (m, 2H), 7.22 − 6.97 (m, 4H), 6.90 (d, *J* = 6.7 Hz, 1H), 5.00 − 4.78 (m, 3H), 4.70 − 4.52 (m, 2H), 4.38 − 4.23 (m, 1H), 4.13 − 4.03 (m, 1H), 3.34 − 3.10 (m, 2H), 3.18 − 3.14 (m, 1H), 2.85 − 2.81 (m, 6H), 2.65 − 2.57 (m, 1H), 2.49 − 2.46 (m, 1H), 2.10 − 2.06 (m, 1H). ^13^C-NMR (CDCl_3_) δ 174.36, 167.53, 161.97 (d, *J* = 222 Hz), 149.13, 148.46, 146.13, 139.01, 135.95, 134.42, 133.20, 133.06, 132.27, 128.72, 128.32, 127.83, 125.46, 125.24, 123.72, 123.32, 122.02, 116.11, 115.90, 115.34, 115.13, 94.45, 61.18, 55.28, 47.56, 41.95, 35.33, 30.30, 28.76, 27.76, 26.28. HPLC: retention time 12.824 min, 99.49% purity. HRMS (ESI): *m/z* calcd for C_34_H_31_FN_6_O_5_, 623.2413, found 623.2424 [M + H]^+^.

#### 3.1.17. *N*-((*S*)-5-Fluoro-2,3-dihydro-1*H*-inden-1-yl)-*N*-(4-fluorobenzyl)-2-(5-(1-(2-(methylamino)-2-oxoethyl)-1*H*-pyrazol-4-yl)-2′,4′-dioxo-2,3-dihydrospiro[indene-1,5′-oxazolidin]-3′-yl)acetamide (**B9**)

Prepared using the same procedure as for **B4**, except using 2-(5-bromo-2′,4′-dioxo-2,3-dihydrospiro[indene-1,5′-oxazolidin]-3′-yl)-*N*-(5-fluoro-2,3-dihydro-1*H*-inden-1-yl)-N-(4-fluorobenzyl) acetamide (**93**, 0.22 g, 0.38 mmol) instead of **88** afforded **B9** (0.08 g, 33.3%). ^1^H-NMR (CDCl_3_) δ 7.88 (s, 1H), 7.71 (s, 1H), 7.61 − 7.52 (m, 1H), 7.43 (t, *J* = 10.5 Hz, 2H), 7.18 (d, *J* = 4.9 Hz, 1H), 7.16 − 6.99 (m, 3H), 6.92 (dd, *J* = 12.5, 7.9 Hz, 3H), 4.91 − 4.76 (m, 3H), 4.65 − 4.60 (m, 1H), 4.51 − 4.44 (m, 1H), 4.27 − 4.20 (m, 1H), 4.12 − 4.03 (m, 1H), 3.40 − 3.07 (m, 2H), 2.92 − 2.76 (m, 6H), 2.67 − 2.56 (m, 1H), 2.47 − 2.42 (m, 1H), 1.92 − 1.83 (m, 1H).^13^C-NMR (101 MHz, CDCl_3_) δ 174.36, 167.90, 167.50, 166.08, 163.59 (d, *J* = 325 Hz), 162.24 (d, *J* = 245 Hz), 154.62, 146.16, 141.41, 138.97, 135.80, 134.54, 132.23, 131.18, 128.76, 128.34, 127.40, 125.49, 125.19, 123.64, 122.04, 116.26, 114.29, 111.87, 106.75, 94.54, 61.90, 59.88, 55.28, 46.18, 41.83, 35.40, 31.49, 30.32, 26.29. HPLC: retention time 13.277 min, 98.78% purity. HRMS (ESI): m/z calcd for C_35_H_31_F_2_N_5_O_5_, 640.2366, found 640.2375 [M + H]^+^.

#### 3.1.18. *N*-((*R*)-2,3-Dihydro-1*H*-inden-1-yl)-*N*-(4-fluorobenzyl)-2-(5-(1-(2-(methylamino)-2-oxoethyl)-1*H*-pyrazol-4-yl)-2′,4′-dioxo-2,3-dihydrospiro[indene-1,5′-oxazolidin]-3′-yl)acetamide (**B10**)

Prepared using the same procedure as for **B4**, except using 2-(5-bromo-2′,4′-dioxo-2,3-dihydrospiro[indene-1,5′-oxazolidin]-3′-yl)-*N*-((*R*)-2,3-dihydro-1*H*-inden-1-yl)-*N*-(4-fluorobenzyl)acetamide (92, 0.10 g, 0.18 mmol) instead of **88** afforded **B10** (0.02 g, 18%). ^1^H-NMR (CDCl_3_) δ 7.89 (s, 1H), 7.72 (s, 1H), 7.61 − 7.54 (m, 1H), 7.44 (t, *J* = 11.1 Hz, 3H), 7.25 − 7.16 (m, 4H), 7.10 − 7.00 (m, 2H), 6.96 − 6.89 (m, 1H), 4.84 (s, 2H), 4.69 − 4.57 (m, 1H), 4.54 − 4.44 (m, 1H), 4.37 − 4.15 (m, 2H), 4.15 − 3.93 (m, 1H), 3.32 − 3.26 (m, 1H), 3.20 − 3.14 (m, 1H), 2.84 (s, 2H), 2.81 (d, *J* = 3.5 Hz, 3H), 2.80 − 2.69 (m, 2H), 2.65 − 2.57 (m, 1H), 2.41 (dd, *J* = 12.2, 5.3 Hz, 1H). ^13^C-NMR (CDCl_3_) δ 174.37, 167.52, 166.73, 166.11, 162.20 (d, *J* = 245 Hz), 146.15, 141.42, 139.00, 135.85, 134.50, 130.96, 128.86, 128.34, 127.32, 125.50, 125.39, 124.30, 123.66, 122.03, 120.53, 116.21, 115.99, 115.21, 106.77, 99.99, 94.51, 60.61, 55.27, 50.92, 41.86, 35.36, 31.12, 30.32, 29.73, 26.30. HPLC: retention time 13.234 min, 99.56% purity (using XBridge C18 (4.6*150 mm, 3.5 μm) instead of Xselect CSH C18 (4.6*150 mm, 3.5 μm), and ammonia instead of trifluoroacetic acid). HRMS (ESI): *m/z* calcd for C_35_H_32_FN_5_O_5_, 622.2460, found 622.2477 [M + H]^+^.

#### 3.1.19. *N*-((*R*)-6,7-Dihydro-5*H*-cyclopenta[b]pyridin-5-yl)-*N*-(4-fluorobenzyl)-2-(5-(1-(2-(methylamino)-2-oxoethyl)-1*H*-pyrazol-4-yl)-2′,4′-dioxo-2,3-dihydrospiro[indene-1,5′-oxazolidin]-3′-yl)acetamide (**B11**)

Prepared using the same procedure as for **B4**, except using 2-(5-bromo-2′,4′-dioxo-2,3-dihydrospiro[indene-1,5′-oxazolidin]-3′-yl)-*N*-((*R*)-2,3-dihydro-1*H*-inden-1-yl)-*N*-((5-fluoropyridin-2-yl)methyl)acetamide (**94**, 0.089 g, 0.16 mmol) instead of 88 afforded **B11** (0.026 g, 26%). ^1^H-NMR (CDCl_3_) δ 7.89 (s, 1H), 7.71 (s, 1H), 7.60 − 7.51 (m, 2H), 7.48 − 7.42 (m, 2H), 7.23 − 7.16 (m, 2H), 7.11 − 7.05 (m, 2H), 6.99 (s, 1H), 6.96 − 6.90 (m, 1H), 5.35 (s, 1H), 4.84 (s, 2H), 4.48 (t, 2H), 4.30 − 4.26 (m, 2H), 3.27 (s, 1H), 3.18 (s, 1H), 2.98 (s, 2H), 2.81 (d, 3H), 2.61 (s, 2H), 2.49 (s, 2H). HPLC: retention time 8.859 min, 96.88% purity. HRMS (ESI): *m/z* calcd for C_34_H_31_FN_6_O_5_, 623.2413, found 623.2425 [M + H]^+^.

#### 3.1.20. *N*-((*R*)-2,3-Dihydro-1*H*-inden-1-yl)-*N*-((5-fluoropyridin-2-yl)methyl)-2-(5-(1-(2-(methylamino)-2-oxoethyl)-1*H*-pyrazol-4-yl)-2′,4′-dioxo-2,3-dihydrospiro[indene-1,5′-oxazolidin]-3′-yl)acetamide (**B12**)

Prepared using the same procedure as for **B4**, except using 2-(5-bromo-2′,4′-dioxo-2,3-dihydrospiro[indene-1,5′-oxazolidin]-3′-yl)-*N*-((*R*)-2,3-dihydro-1*H*-inden-1-yl)-*N*-((5-fluoro-pyridin-2-yl)methyl)acetamide (**95**, 0.11 g, 0.20 mmol) instead of **88** afforded **B12** (0.05 g, 39%). ^1^H-NMR (CDCl_3_) δ 8.42 (s, 1H), 8.27 (s, 1H), 7.88 (s, 1H), 7.71 (s, 1H), 7.66 − 7.49 (m, 2H), 7.44 (t, *J* = 8.0 Hz, 2H), 7.33 (d, *J* = 7.8 Hz, 1H), 7.21 (s, 2H), 7.16 − 7.02 (m, 2H), 4.95 − 4.79 (m, 3H), 4.73 − 4.63 (m, 1H), 4.53 − 4.46 (m, 1H), 4.39 − 4.33 (m, 1H), 4.27 − 4.18 (m, 1H), 3.38 − 3.10 (m, 2H), 3.02 − 2.97 (m, 1H), 2.92 − 2.76 (m, 6H), 2.62 (d, *J* = 5.1 Hz, 1H), 2.49 − 2.36 (m, 1H). ^13^C-NMR (CDCl_3_) δ 174.36, 167.50, 166.28, 158.69 (d, *J* = 255 Hz), 154.63, 153.63, 146.13, 144.03, 141.38, 140.24, 138.95, 137.97, 135.96, 134.47, 128.82, 128.32, 127.22, 125.49, 124.98, 124.83, 123.70, 122.03, 121.75, 94.56, 94.42, 60.47, 55.28, 47.95, 41.93, 35.41, 30.50, 30.31, 29.70, 26.27. HPLC: retention time 11.706 min, 94.84% purity. HRMS (ESI): *m/z* calcd for C_34_H_31_FN_6_O_5_, 623.2413, found 623.2435 [M + H]^+^.

#### 3.1.21. *N*-((*R*)-6,7-Dihydro-5*H*-cyclopenta[b]pyridin-7-yl)-*N*-((5-fluoropyridin-2-yl) methyl)-2-(5-(1-(2-(methylamino)-2-oxoethyl)-1*H*-pyrazol-4-yl)-2′,4′-dioxo-2,3-dihydrospiro[indene-1,5′-oxazolidin]-3′-yl)acetamide (**B13**)

Prepared using the same procedure as for **B4**, except using 2-(5-bromo-2′,4′-dioxo-2,3-dihydrospiro [indene-1,5′-oxazolidin]-3′-yl)-*N*-((*R*)-6,7-dihydro-5*H*-cyclopenta[*b*]pyridin-7-yl)-*N*-((5-fluoropyridin-2-yl)methyl)acetamide (**96**, 0.20 g, 0.36 mmol) instead of **88** afforded **B13** (0.093 g, 41%). ^1^H-NMR (CDCl_3_) δ 8.41 (s, 1H), 8.20 (s, 1H), 7.87 (s, 1H), 7.71 (s, 1H), 7.62 − 7.49 (m, 3H), 7.41 (d, *J* = 7.1 Hz, 2H), 7.30 (d, *J* = 5.3 Hz, 1H), 7.16 − 7.09 (m, 1H), 5.10 − 4.97 (m, 1H), 4.82 (s, 2H), 4.71 − 4.57 (m, 2H), 4.51 − 4.39 (m, 1H), 4.36 − 4.22 (m, 1H), 3.35 − 3.21 (m, 1H), 3.17 (s, 1H), 3.09 − 2.97 (m, 1H), 2.95 − 2.75 (m, 6H), 2.66 − 2.57 (m, 1H), 2.56 − 2.47 (m, 1H). ^13^C-NMR (CDCl_3_) δ 167.51, 165.90, 160.60, 159.74, 154.62, 153.63 (d, *J* = 226 Hz), 149.05, 148.50, 146.10, 138.96, 138.13, 137.89, 136.49, 135.97, 134.45, 133.16, 131.25, 128.31, 125.44, 123.69, 123.26, 122.71, 122.00, 94.50, 62.15, 55.27, 49.23, 41.86, 35.40, 30.30, 28.27, 27.82, 26.27. HPLC: retention time 8.882 min, 95.24% purity. HRMS (ESI): *m/z* calcd for C_33_H_30_FN_7_O_5_, 624.2365, found 624.2375 [M + H]^+^.

#### 3.1.22. *N*-(4-Fluorobenzyl)-*N*-((*S*)-1-methyl-4,5,6,7-tetrahydro-1*H*-indazol-4-yl)-2-(5-(1-(2-(methylamino)-2-oxoethyl)-1*H*-pyrazol-4-yl)-2′,4′-dioxo-2,3-dihydrospiro [indene-1,5′-oxazolidin]-3′-yl)acetamide (**B14**)

Prepared using the same procedure as for **B4**, except using 2-(5-bromo-2′,4′-dioxo-2,3-dihydrospiro[indene-1,5′-oxazolidin]-3′-yl)-*N*-(4-fluorobenzyl)-*N*-((*S*)-1-methyl-4,5,6,7-tetrahydro-1*H*-indazol-4-yl) acetamide (**97**, 0.15 g, 0.27 mmol) instead of **88** afforded **B14** (0.059 g, 35%). ^1^H-NMR (CDCl_3_) δ 7.89 (s, 1H), 7.71 (s, 1H), 7.57 (dd, *J* = 14.2, 6.2 Hz, 1H), 7.44 (s, 2H), 7.18 − 7.13 (m, 1H), 7.06 (t, *J* = 7.5 Hz, 2H), 6.93 (d, *J* = 5.6 Hz, 2H), 4.83 (s, 2H), 4.79 − 4.62 (m, 1H), 4.49 (m, 1H), 4.44 − 4.34 (m, 2H), 4.27 − 4.10 (m, 1H), 3.28 (dt, *J* = 14.7, 7.4 Hz, 1H), 3.23 − 3.12 (m, 1H), 3.11 − 2.95 (m, 1H), 2.89 (dd, *J* = 14.4, 7.4 Hz, 1H), 2.82 (d, *J* = 4.5 Hz, 3H), 2.73 − 2.55 (m, 2H), 2.55 − 2.44 (m, 1H), 2.41 − 2.08 (m, 3H). ^13^C-NMR (CDCl_3_) δ 174.59, 167.48, 165.11, 153.24 (d, *J* = 278 Hz), 151.85, 146.15, 138.98, 135.82, 134.52, 133.90, 133.99, 133.87, 132.32, 129.09, 128.31, 127.68, 127.59, 125.50, 123.67, 122.02, 116.12, 115.90, 94.51, 55.20, 54.04, 46.30, 41.97, 39.50, 36.93, 35.52, 35.40, 30.31, 26.27, 21.70. HPLC: retention time 10.549 min, 97.97% purity. HRMS (ESI): *m/z* calcd for C_34_H_34_FN_7_O_5_, 626.2522, found 626.2541 [M + H]^+^.

#### 3.1.23. *N*-(4-fluorobenzyl)-2-(5-(1-(2-(methylamino)-2-oxoethyl)-1*H*-pyrazol-4-yl)-2′,4′-dioxo-2,3-dihydrospiro[indene-1,5′-oxazolidin]-3′-yl)-*N*-((*S*)-1,2,3,4-tetrahydronaphthalen-1-yl)acetamide (**B15**)

To a solution of 2-(5-bromo-2′,4′-dioxo-2,3-dihydrospiro[indene-1,5′-oxazolidin]-3′-yl)-*N*-(4-fluorobenzyl)-*N*-((*S*)-1,2,3,4-tetrahydronaphthalen-1-yl)acetamide (**98**, 0.15 g, 0.26 mmol), *N*-methyl-2-(4-(4,4,5,5-tetramethyl-1,3,2-dioxaborolan-2-yl)-1*H*-pyrazol-1-yl) acetamide (0.06 g, 0.29 mmol) and Pd(dppf)Cl_2_ (0.02 g, 0.026 mmol) in 1,4-dioxane (5 mL) was added a saturated solution of sodium bicarbonate (0.5 mL) under nitrogen. The mixture was stirred in 85 °C until completion, quenched with water (10 mL), and extracted with ethyl acetate. The organic phase was combined, dried over anhydrous Na_2_SO_4_, and concentrated under vacuum. The residue was purified by silica gel column chromatography (EA:PE = 1:1) to afford **B15** (0.09 g, 56%). ^1^H-NMR (DMSO-*d*_6_) δ 8.20 (s, 1H), 8.03 (s, 1H), 7.94 (s, 1H), 7.63 (s, 1H), 7.55 − 7.48 (m, 2H), 7.38 (s, 1H), 7.20 (d, *J* = 12.3 Hz, 4H), 7.13 − 7.08 (m, 2H), 4.92 − 4.55 (m, 5H), 4.40 − 3.67 (m, 2H), 3.26 − 3.05 (m, 2H), 2.76 − 2.69 (m, 2H), 2.63 (d, *J* = 3.8 Hz, 3H), 2.59 − 2.53 (m, 1H), 2.13 − 1.58 (m, 5H). ^13^C-NMR (DMSO-d_6_) δ 174.26, 167.32, 166.81, 161.76 (d, *J* = 242 Hz), 154.74, 146.61, 138.93, 137.45, 135.71, 135.63, 135.22, 134.34, 129.96, 129.63, 129.12, 128.55, 127.79, 127.41, 126.74, 126.56, 125.03, 121.89, 121.78, 116.04, 115.19, 94.27, 56.93, 54.74, 46.86, 42.03, 35.16, 30.26, 29.48, 29.08, 26.09, 22.03. HPLC: retention time 15.903 min, 97.92% purity. HRMS (ESI): *m/z* calcd for C_36_H_34_FN_5_O_5_, 636.2617, found 636.2621 [M + H]^+^.

#### 3.1.24. *N*-((*S*)-chroman-4-yl)-*N*-(4-fluorobenzyl)-2-(5-(1-(2-(methylamino)-2-oxoethyl)-1H-pyrazol-4-yl)-2′,4′-dioxo-2,3-dihydrospiro[indene-1,5′-oxazolidin]-3′-yl) acetamide (**B16**)

Prepared using the same procedure as for **B15**, except using 2-(5-bromo-2′,4′-dioxo-2,3-dihydrospiro[indene-1,5′-oxazolidin]-3′-yl)-*N*-((*S*)-chroman-4-yl)-*N*-(4-fluorobenzyl)acetamide (**99**, 0.30 g, 0.52 mmol) instead of **98** afforded **B16** (0.11 g, 33%). ^1^H-NMR (CDCl_3_) δ 7.85 (s, 1H), 7.71 (s, 1H), 7.58 − 7.50 (m, 1H), 7.40 (d, *J* = 13.2 Hz, 2H), 7.22 (s, 1H), 7.19 − 7.10 (m, 2H), 7.10 − 7.02 (m, 2H), 6.97 − 6.87 (m, 2H), 6.79 (s, 1H), 5.15 − 4.81 (m, 1H), 4.81 (s, 2H), 4.59 − 4.45 (m, 2H), 4.28 − 4.05 (m, 4H), 3.32 − 3.20 (m, 1H), 3.14 (dd, *J* = 11.8, 8.0 Hz, 1H), 2.83 (d, *J* = 7.8 Hz, 1H), 2.78 (d, *J* = 4.1 Hz, 3H), 2.63 − 2.53 (m, 1H), 2.06 (s, 1H), 1.98 (d, *J* = 10.1 Hz, 1H). ^13^C-NMR (CDCl_3_) δ 174.32, 167.51, 166.83, 166.80, 162.98 (d, *J* = 245 Hz), 156.45, 154.59, 146.14, 138.82, 135.73, 134.62, 129.27, 128.40, 127.71, 127.35, 125.44, 125.09, 123.52, 122.04, 121.24, 121.17, 120.10, 117.34, 116.32, 116.10, 94.60, 64.92, 55.22, 50.36, 46.66, 41.76, 35.31, 30.29, 27.17, 26.27. HPLC: retention time 12.527 min, 99.97% purity. HRMS (ESI): *m/z* calcd for C_35_H_32_FN_5_O_6_, 638.2409, found 638.2415 [M + H]^+^.

#### 3.1.25. 1-(3′-(2-(7-(Difluoromethyl)-3,4-dihydroquinolin-1(2*H*)-yl)-2-oxoethyl)-2′,4′-dioxo-2,3-dihydrospiro[indene-1,5′-oxazolidin]-5-yl)-3-methylurea (**B17**)

To a solution of 5-amino-3′-(2-(7-(difluoromethyl)-3,4-dihydroquinolin-1(2*H*)-yl)-2-oxoethyl)-2,3-dihydrospiro[indene-1,5′-oxazolidine]-2′,4′-dione (**100**, 0.12 g, 0.27 mmol) and triethylamine (0.08 g, 0.816 mmol) in anhydrous tetrahydrofuran (2 mL) was added triphosgene (0.08 g, 0.27 mmol). The mixture was stirred at room temperature for 30 min. Then methylamine in tetrahydrofuran (2.0 M, 0.41 mL) was added. The mixture was stirred for 1 h, quenched with a saturated solution of sodium bicarbonate (10 mL) and extracted with ethyl acetate. The organic phase was combined, dried over anhydrous Na_2_SO_4_, and concentrated under vacuum. The residue was purified by silica gel column chromatography (EA:PE = 1:1) to afford **B17** (0.03 g, 22%). ^1^H-NMR (DMSO-d_6_) δ 7.97 (s, 1H), 7.55 (s, 1H), 7.39 − 7.21 (m, 4H), 6.13 (d, *J* = 4.2 Hz, 1H), 4.69 (s, 2H), 3.89 − 3.77 (m, 2H), 3.35 (s, 1H), 3.18 − 3.07 (m, 2H), 3.04 − 2.97 (m, 1H), 2.81 (s, 2H), 2.64 (d, *J* = 4.3 Hz, 3H), 1.94 (s, 2H). ^13^C-NMR (DMSO-d_6_) δ 174.39, 165.39, 156.11, 154.68, 146.84, 143.73, 138.08, 132.17, 130.04, 129.92, 124.73, 122.14, 117.60, 117.41, 115.26 (t, *J* = 235 Hz), 113.78, 112.92, 94.46, 49.07, 46.13, 42.44, 35.45, 30.35, 26.68, 23.44. HPLC: retention time 11.429 min, 96.29% purity (using formic acid instead of trifluoroacetic acid). HRMS (ESI): *m/z* calcd for C_25_H_24_F_2_N_4_O_5_, 499.1788, found 499.1808 [M + H]^+^.

#### 3.1.26. 5-(1-Methyl-1*H*-pyrazol-4-yl)-3′-(2-oxo-2-(3-(trifluoromethyl)-5,6-dihydro-[1,2,4]triazolo[4,3-a]pyrazin-7(8*H*)-yl)ethyl)-2,3-dihydrospiro[indene-1,5′-oxazolidine]-2′,4′-dione (**B18**)

Prepared using the same procedure as for **B15**, except using 5-bromo-3′-(2-oxo-2-(3-(trifluoromethyl)-5,6-dihydro-[1,2,4]triazolo[4,3-*a*]pyrazin-7(8*H*)-yl)ethyl)-2,3-dihydrospiro[indene-1,5′-oxazolidine]-2′,4′-dione (**101**, 0.10 g, 0.20 mmol) instead of 98 afforded **B18** (0.034 g, 33%). ^1^H-NMR (CDCl_3_) δ 7.76 (s, 1H), 7.62 (s, 1H), 7.47 (d, *J* = 7.3 Hz, 1H), 7.43 (s, 1H), 7.26 (s, 1H), 5.10 − 5.01 (m, 2H), 4.61 − 4.47 (m, 2H), 4.23 (s, 1H), 4.16 (s, 1H), 3.95 (s, 2H), 3.49 (s, 3H), 3.34 − 3.23 (m, 1H), 3.21 − 3.10 (m, 1H), 2.89 − 2.78 (m, 1H), 2.65 − 2.56 (m, 1H). ^13^C-NMR (CDCl_3_) δ 174.53, 163.72, 154.29, 152.43, 148.55, 146.11, 136.97, 135.70, 134.85, 129.458, 127.38, 124.77, 123.96 (q, *J* = 273 Hz), 94.93, 43.31, 43.09, 41.74, 40.96, 39.20, 35.42, 30.30, 12.33. HPLC: retention time 9.839 min, 96.11% purity (using formic acid instead of trifluoroacetic acid). HRMS (ESI): *m/z* calcd for C_23_H_20_F_3_N_7_O_4_, 516.1602, found 516.1618 [M + H]^+^.

### 3.2. Cell Viability Assays

The 22Rv1 prostate cancer cell line in this study was purchased from KeyGEN Biotech Co. Ltd. (Nanjing, Jiangsu, China ). The cells were cultured in RPMI 1640 medium and maintained at 37 °C in a 5% CO_2_ incubator. For the cell viability assay, the cell slurry was diluted to required volume at a density of 80,000 cells/mL. Then, 100 μL the cell slurry was seeded in each well of the 96-well plate. The cells were incubated for 72 h at 37 °C, 5% CO_2_ under humidified condition. The reference and test compounds solution (200×) were prepared with DMSO and were diluted from 200× to 1× with culture medium (RPMI 1640 medium without phenol red and containing 10% fetal calf serum, penicillin (100U/mL), streptomycin (100 μg/mL) and 0.1 nM DHT). The previous culture medium was removed and 200 μL/well of the compound solution (1×) was added to assay plate (final conc: 1×). Then the cells were incubated at 37 °C, 5% CO_2_ under humidified conditions. 5 Days later, the CCK-8 assay reagent was equilibrated at room temperature for 30 min prior to use. CCK-8 was mixed with fresh medium at a ratio of 1:10 and 100 μL of the diluted CCK8 reagent was added to each well and the plates were incubated for 4.5 h at 37 °C. Finally, absorbance value (OD value) was read on plate reader. GraphPad Prism v5.0 software was used to process data for IC_50_.

### 3.3. Pharmacokinetics Procedures

Pharmacokinetic experiments of test compounds were performed in male Balb/C mice similarly to our previous work [38]. The mice were randomly assigned to two groups and were administrated the test compound orally and intravenously, respectively. The test compounds were prepared into 0.5 mg/mL oral solution with 10%PG, 10% ethanol, 10% solutol and 70% normal saline, or 0.1 mg/mL injection solution with 2% PG, 2% ethanol, 2% solutol and 94% normal saline. After intravenous or oral administration, blood was collected from the orbital venous plexus into heparinized EP tube (0.6 mL) at 5, 15, 30 min, 1, 2, 6, 10, and 24 h, temporarily placed on crushed ice. Certain processing was performed on blood samples and testing samples were sent to LC-MS/MS for analysis.

### 3.4. In Vivo Tumor Xenograft Model

A well-established tumorigenesis assay was used to evaluate the antitumor effect of **B16-P2** in male NOD-SCID mice model. All mice were raised in standard specific-pathogen-free (SPF) environment. Mice were randomly allocated to three groups (6 mice in each group) by an independent person in the laboratory. No statistical method was used to predetermine sample size. 5 × 10^6^ 22Rv1 cells (purchased from Nanjing KeyGEN Biotech Co. Ltd.) were injected subcutaneously into the NOD-SCID male mice at 5-to-6-week-old (purchased from Shanghai lingchang animal Co. Ltd.). All compounds were prepared into solution using 5% ethanol, 30% PG, 25% PEG400, 10% solutol, and 30% pure water successively and were administrated by oral gavage. Mice were examined thrice a week for the development of tumors by Vernier caliper and tumor volumes were calculated using the formula V = 0.5 × length × width^2^. The investigators were not blinded to allocation during experiments and outcome assessment. The antitumor effects of the compounds were assessed by tumor growth inhibition (TGI) or relative tumor proliferation rate (T/C): TGI (%) = [1− (V_t1_ − V_t0_)/(V_c1_ − V_c0_)]×100%, where V_c1_ and V_t1_ are the mean volumes of control and treated groups at time of tumor extraction, while V_c0_ and V_t0_ are the same groups at the start of dosages; T/C (%) = T_RTV_/C_RTV_ × 100%, where T_RTV_ is the relative tumor volume (RTV) of treated groups, while C_RTV_ is the RTV of control groups. (RTV = V_t_/V_0_, V_t_ is the mean volumes of treated groups at time of tumor extraction, V_0_ is the mean volumes of the same groups at the start of dosages).

### 3.5. Molecular Dynamic Simulation and Docking

Molecular dynamic simulation was performed with Desmond in Schrodinger Maestro 2019 using OPLS_2005 as force field (PDB code: 5kj2). The systems were solvated in TIP3P water molecules in a truncated octahedron periodic box, then neutralized by adding Na+ cations. After energy minimization, a sum of production simulation was performed for 100 ns using the NPT ensemble under a constant temperature of 300 K and pressure of 1 atm. Other parameters were maintained at the default configuration. Finally, the binding free energies for the complexes were calculated by Prime/MM-GBSA module. Molecular docking was performed with Glide module in Schrodinger Maestro 2019, with OPLS_2005 as force field and Extra Precision (XP) as algorithm.

## 4. Conclusions

In summary, twenty-six new compounds based on the 2-(2′,4′-dioxo-2,3-dihydrospiro[indene-1,5′-oxazolidine]-3′-yl)acetamide scaffold were designed based on a bioisosterism and conformational restriction strategy. Their antiproliferative activities against enzalutamide resistant prostate cancer 22Rv1 cell were evaluated. A comprehensive SAR study was concluded, leading to the strongest inhibitor **B16**. Molecular docking predicted the possible binding mode with p300 HAT domain. The additional hydrogen bond between chromane oxygen of **B16** and HAT protein was of critical importance for the observed stronger activity. Furthermore, compound **B16** exhibited suitable PK properties. The in vivo 22Rv1 xenograft model revealed that compound **B16-P2** inhibited tumor growth stronger than A-485 at the same dosage. In general, our results suggest that these spirocyclic chromane derivatives were a class of promissing therapeutic agents of prostate cancer for further optimization.

## Figures and Tables

**Table 1:**
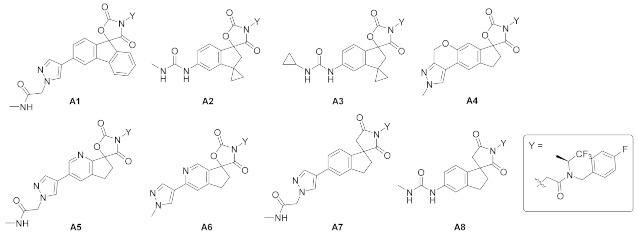
22Rv1 cell antiproliferation activity on optimization of core scaffolds ^a^.

Compd.	IC_50_ (µM)	Compd.	IC_50_ (µM)
**A-485**	0.184	**A5**	16.080
**A1**	>100	**A6**	26.970
**A2**	0.351	**A7**	1.798
**A3**	0.798	**A8**	6.412
**A4**	>100		

^a^ Values are means of two independent experiments (*n* = 2).

**Table 2:**
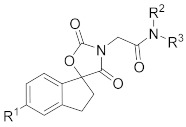
Effect of optimization of R1, R2 and R3 on 22Rv1 cell antiproliferation activity ^a^.

Compd.	R^1^	R^2^	R^3^	IC_50_ (µM)
**A-485 ^b^**	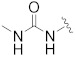	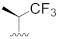	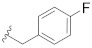	0.184
**B1 ^b^**	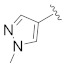	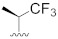	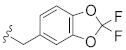	1.369
**B2 ^b^**	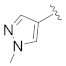	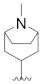	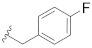	37.620
**B3**	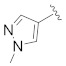	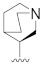	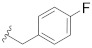	>25
**B4**	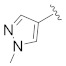	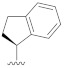	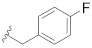	0.252
**B5**	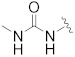	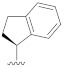	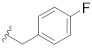	0.406
**B6**	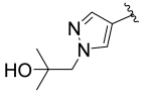	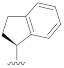	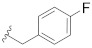	0.431
**B7**	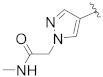	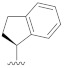	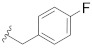	0.270
**B8**	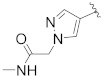	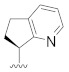	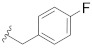	0.153
**B9**	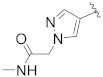	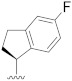	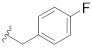	0.334
**B10**	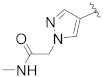	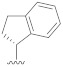	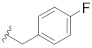	1.660
**B11**	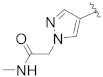	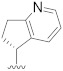	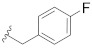	0.340
**B12**	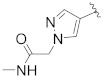	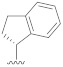	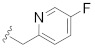	0.265
**B13**	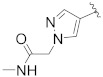	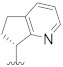	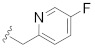	3.472
**B14**	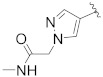	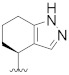	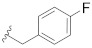	1.093
**B15**	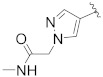	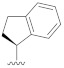	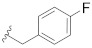	0.450
**B16**	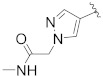	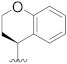	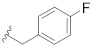	0.096
**B17**	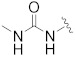	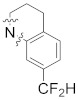		8.701
**B18**	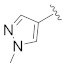	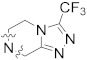		>25

^a^ Values are means of two independent experiments (*n* = 2). ^b^ Spiro chirality has an *R*-configuration.

**Table 3:** Pharmacokinetic profiles of selected compounds ^a^.

Compd.	IV. (1 mg/kg)				PO. (10 mg/kg)			
	T_1/2_ ^b^ (h)	C_L_ ^c^ (L/h/kg)	V _ss (0–24 h)_ ^d (^h∗ng/mL)	AUC_(0–24h)_ (h∗ng/mL)	T_1/2_ (h)	C_max_ ^e^ (ng/mL)	AUC _(0–24 h) (_h∗ng/mL)	F ^f^ (%)
**B8**	17.0 ± 4.9	1.1 ± 0.1	13.2 ± 4.2	766.5 ± 71.2	23.9 ± 3.6	878.3 ± 94.3	1656 ± 293.9	21.6 ± 3.8
**B16**	0.5 ± 0.0	1.8 ± 0.1	1.0 ± 0.1	535.9 ± 18.1	1.6 ± 0.3	759.3 ± 76.5	1779.3 ± 158.3	33.2 ± 3.0
**A-485**	0.7 ± 0.1	0.5 ± 0.1	0.5 ± 0.1	1995.9 ± 597.7	3.1 ± 1.1	2700 ± 518.8	10,796.7 ± 1493.0	54.1 ± 7.5

^a^ Values are means of three independent experiments (*n* = 3). ^b^ Half-life. ^c^ Clearance. ^d^ Volume of distribution at steady state. ^e^ Maximum concentration observed. ^f^ Oral bioavailability, ∗ means multiplication sign.

**Table 4:** 22Rv1 cell antiproliferation and pharmacokinetic profiles of two isomers of **B16** ^a^.

Compd.	22Rv1 Cell		Pharmacokinetics, PO. (10 mg/kg)			
	IC_50_ (µM)	TOP (%)	T_1/2_ (h)	C_max_ (ng/mL)	AUC_(0–24 h)_ (h∗ng/mL)	MRT_(0–24 h)_ (h)
**B16-P1**	0.150	80.6	0.9 ± 0.1	488.3 ± 139.3	951.9 ± 386.1	1.2 ± 0.3
**B16-P2**	0.149	87.8	1.2 ± 0.1	428.0 ± 66.7	694.6 ± 166.1	1.3 ± 0.1

^a^ Values are means of two independent experiments (cell antiproliferation, *n* = 2) and three independent experiments (PK, *n* = 3), ∗ means multiplication sign.

## Data Availability

Not applicable.

## Supplementary Materials

The following are available online. Experimental procedures for preparation of compounds, chemical spectrum and purity determination.

## Sample Availability

Samples of the compounds are available from the authors.
