# Supplementary material for: Synthesis and Biological Evaluation of Spirocyclic Chromane Derivatives as a Potential Treatment of Prostate Cancer
Source: Molecules. 2021 May 25;26(11):3162. doi: 10.3390/molecules26113162 (PMC8198214; doi:10.3390/molecules26113162)

Supplementary data.

Synthesis and Biological Evaluation of Spirocyclic Chromane Derivatives as Novel p300/CBP Histone Acetyltransferases Inhibitors

Li Feng[a,b,#], Shujia Yu[b,#], Hai Wang[b], Shengwei Yang[b], Xue Li[b], Hongjuan Dai[c], Liwen Zhao[b,*], Cheng Jiang[a,*] and Yazhou Wang[b,*]

[a] Department of Medicinal Chemistry, China Pharmaceutical University, Tongjiaxiang 24, Nanjing 210009, P. R. China

[b] Nanjing Sanhome Pharmaceutical Co. Ltd., No. 99, West Yunlianghe Road, Jiangning District, Nanjing, 210049, P. R. China.

[c] Quality Department, Aurovitas Pharma Taizhou Co. Ltd., Taizhou, Jiangsu Province, 225300, P. R. China

[*]Corresponding Authors. Tel: +86-25-81066791; E-mail: wangyzyf@sanhome.com (Y. Wang), zhaolw@sanhome.com (L. Zhao), jc@cpu.edu.cn (C. Jiang)

[#]These authors contributed equally.

Contents

1. Experimental procedures for preparation of compounds.
2. Chemical spectrum and purity determination

Experimental procedures for preparation of compounds.

Scheme S1

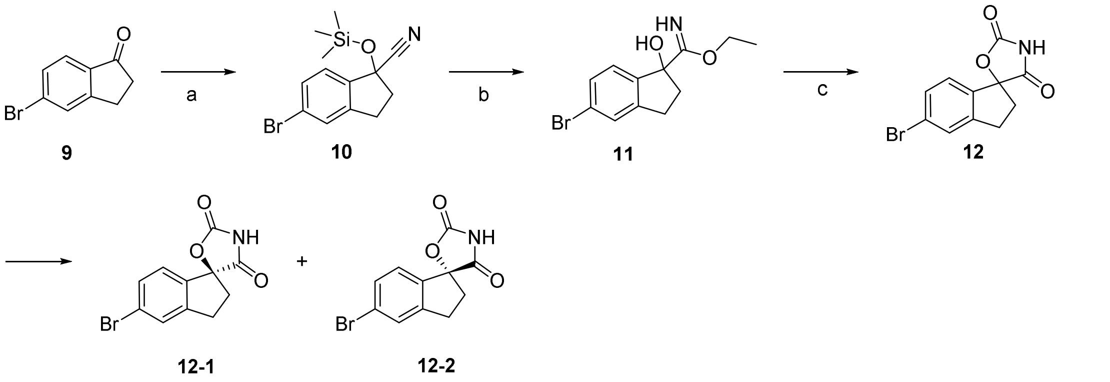

Reagents and conditions: (a) TMSCN, NMO, acetonitrile, 60 °C; (b) AcCl, EtOH, 8 °C; (c) TFA, triphosgene, 10 °C;

5-Bromo-1-((trimethylsilyl)oxy)-2,3-dihydro-1*H*-indene-1-carbonitrile (10): A solution of **9** (5.0 g, 23.81 mmol), TMSCN (4.7 g, 47.63 mmol) and NMO (0.84 g, 7.14 mmol) in acetonitrile (40 mL) was heated to reflux overnight at 60 °C under nitrogen. After completion, the solution was concentrated and the residue was purified by silica gel column chromatography (EA:PE=1:10) to afford **10** (5.96 g, 81%).

Ethyl 5-bromo-1-hydroxy-2,3-dihydro-1*H*-indene-1-carbimidate (11): To a solution of **10** (5.5 g, 15.94 mmol) in ethanol (50 mL) was added acetyl chloride (45.59 mL) dropwise at 0 °C. After completion, the solvent was concentrated, diluted with water (50 mL), and extracted with ethyl acetate. The organic phase was combined, dried over anhydrous Na_2SO_4, and concentrated to afford **11** (4.2 g, 76%).

5-Bromo-2,3-dihydrospiro[indene-1,5'-oxazolidine]-2',4'-dione (12): To a solution of **11** (4.2 g, 12.15 mmol) and triethylamine (4.9 g, 6.8 mL) in THF (20 mL) was added triphosgene (1.44 g, 4.86 mmol) at 10 °C. The mixture was stirred for 1 h. After cooling the mixture to 5 °C, 2 N hydrochloric acid (20 mL) was added and stirred for 5 minutes. The mixture was extracted with ethyl acetate. The organic layers were combined and dried over anhydrous Na_2SO_4. The organic layers were concentrated to afford **12** (3.5 g, 93%). The racemates (**12-1** & **12-2**) were separated by chiral HPLC (ChiralPak AD-H column, 20% isopropyl alcohol:80% hexane).

Scheme S2

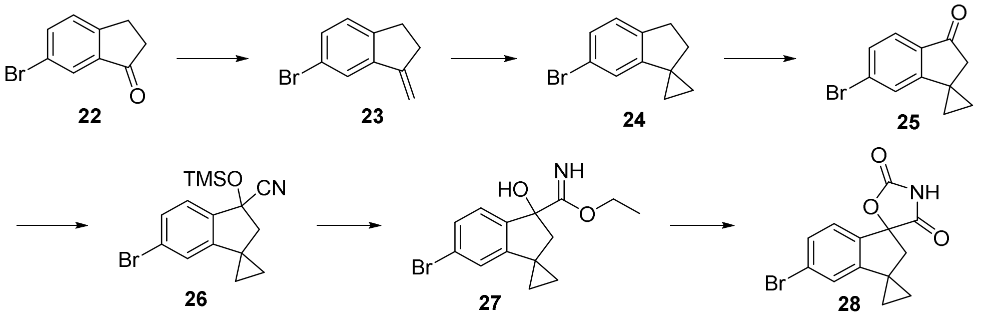

6-Bromo-1-methylene-2,3-dihydro-1*H*-indene (23): A solution of methyl triphenylphosphonium bromide (28.4 g, 79.6 mmol) in dry THF (100 mL) was stirred under -5 °C for 5 minutes. A solution of t-BuOK (10.4 g, 92.7 mmol) in dry THF was added dropwise and stirred for 1 hour at -5 °C. A solution of **22** (14 g, 66.4 mmol) in dry THF was added dropwise. After stirred for 2 h at -5 °C, the reaction was quenched with water (100 mL), warmed to room temperature, and extracted with ethyl acetate. The organic layers were concentrated. The residue was purified by silica gel column chromatography (PE) to afford **23** (10g, 60%).

6'-Bromo-2',3'-dihydrospiro[cyclopropane-1,1'-indene] (24): To a solution of diethyl zinc (144.2 ml, 1 mol/L) in methylene chloride (100 mL) was added a solution of TFA (16.4 g, 10.7 mL) in methylene chloride (40 mL) dropwise at -5 °C. After stirred for 1 hour, a solution of diiodomethane (38.6 g, 11.63 mL) in methylene chloride (20 mL) was added at -5 °C and stirred for 1 hour. A solution of **23** (10.0 g, 48.08 mmol) in methylene chloride was added. After 5 minutes, the mixture was warmed to room temperature and stirred overnight. The solution was concentrated, treated with 1 N HCl, and extracted with methylene chloride. The organic layers were combined and concentrated. The residue was purified by silica gel column chromatography (PE) to afford **24** (10.1 g, 94%).

6'-Bromospiro[cyclopropane-1,1'-inden]-3'(2'*H*)-one (25): To a solution of **24** (11.5 g, 51.8 mmol) and $MgSO_4$ solution (1.5 M aq., 58.5 mL) in acetone (160 mL) was added $KMnO_4$ (9.0 g, 56.96 mmol) portionwise at 0 °C and stirred overnight. The mixture was filtered through celite. The filtrate was extracted with ethyl acetate. The organic layers were combined and concentrated. The residue was purified by silica gel column chromatography (EA:PE=1:7) to afford **24** (4.2 g, 34%).

6'-Bromo-3'-((trimethylsilyl)oxy)-2',3'-dihydrospiro[cyclopropane-1,1'-indene]-3'-carbonitrile (26): Prepared using the same procedure as for **10**, except using 6'-bromospiro[cyclopropane-1,1'-inden]-3'(2'*H*)-one (**25**, 4.3 g, 18.1 mmol) instead of **9** afforded **26** (5.5 g, 84%).

Ethyl 6'-bromo-3'-hydroxy-2',3'-dihydrospiro[cyclopropane-1,1'-indene]-3'-

carbimidate (27): Prepared using the same procedure as for **11**, except using 6'-bromo-3'-((trimethylsilyl)oxy)-2',3'-dihydrospiro[cyclopropane-1,1'-indene]-3'-carbonitrile (**26**, 5.5 g, 15.94 mmol) instead of **10** afforded **27** (4.19 g, 76%).

5'-Bromo-2'*H*-dispiro[cyclopropane-1,3'-indene-1',5''-oxazolidine]-2'',4''-dione (28): Prepared using the same procedure as for **12**, except using ethyl 6'-bromo-3'-hydroxy-2',3'-dihydrospiro[cyclopropane-1,1'-indene]-3'-carbimidate (**27**, 4.19 g, 12.15 mmol) instead of **11** afforded **28** (3.5 g, 93%).

Scheme S3

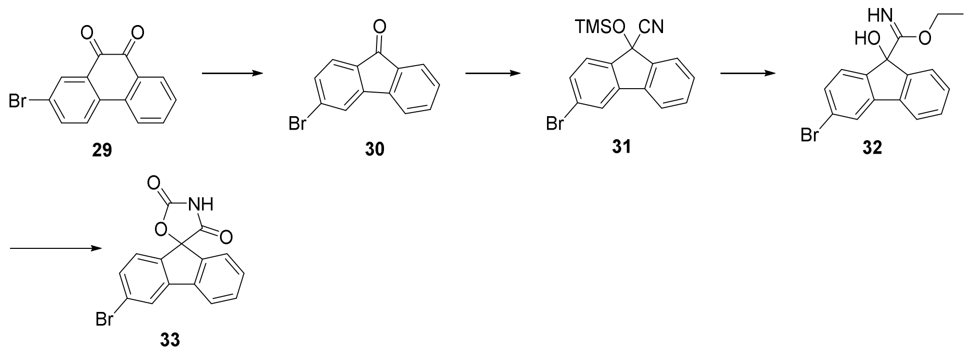

3-Bromo-9*H*-fluoren-9-one (30): To a solution of **29** (3.0 g, 10.45 mmol) in KOH solution (7.3 M, 90 mL) was added potassium permanganate (7.44g, 47.03 mmol) at room temperature. The mixture was stirred at 110 °C for 1 h. After cooling to room temperature, the mixture was diluted with ethanol (50 mL), a saturated solution of sodium bisulfite (50 mL) and 50% sulfuric acid (5 mL). The mixture was filtered and the filter cake was washed with methylene chloride. The filtrate was extracted with methylene chloride. The organic layers were combined and concentrated. The residue was purified by silica gel column chromatography (EA:PE=1:5) to afford **29** (0.86 g, 32%).

3-Bromo-9-((trimethylsilyl)oxy)-9*H*-fluorene-9-carbonitrile (31): Prepared using the same procedure as for **10**, except using 3-bromo-9*H*-fluoren-9-one (**30**, 0.86 g, 3.33 mmol) instead of **9** afforded **31** (0.62 g, 52%).

Ethyl 3-bromo-9-hydroxy-9*H*-fluorene-9-carbimidate (32): Prepared using the same procedure as for **11**, except using 3-bromo-9-((trimethylsilyl)oxy)-9*H*-fluorene-9-carbonitrile (**31**, 0.6 g, 1.74 mmol) instead of **10** afforded **32** (0.27 g, 46%).

3-Bromospiro[fluorene-9,5'-oxazolidine]-2',4'-dione (33): Prepared using the same procedure as for **12**, except using ethyl 3-bromo-9-hydroxy-9*H*-fluorene-9-carbimidate (**32**, 0.27 g, 0.803 mmol) instead of **11** afforded **33** (0.26 g, 98%).

Scheme S4

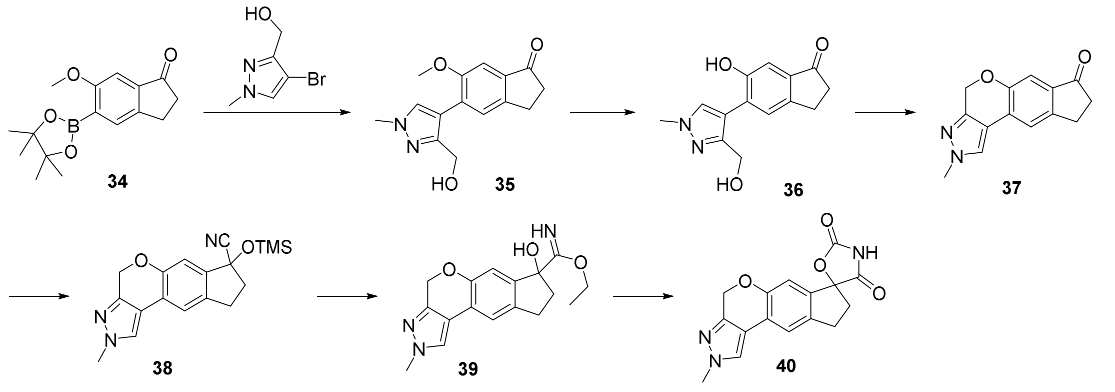

5-(3-(Hydroxymethyl)-1-methyl-1*H*-pyrazol-4-yl)-6-methoxy-2,3-dihydro-1*H*-inden-1-one (35): To a solution of **34** (1.0 g, 3.59 mmol), (4-bromo-1-methyl-1H-pyrazol-3-yl) methanol (0.682 g, 3.59 mmol) and Pd(dppf)Cl_2 (0.20 g, 0.36 mmol) in 1,4-dioxane (10 mL) were added a saturated solution of sodium bicarbonate (2 mL) under nitrogen. The mixture was stirred at 85 °C overnight, cooled to room temperature, quenched with water (2 mL), and extracted with ethyl acetate. The organic layers were combined and concentrated. The residue was purified by silica gel column chromatography (EA:PE=1:1) to afford **35** (0.60 g, 61%).

6-Hydroxy-5-(3-(hydroxymethyl)-1-methyl-1*H*-pyrazol-4-yl)-2,3-dihydro-1*H*-inden-1-one (36): To a solution of $AlCl_3$ (1.2 g, 17.64 mmol) in methylene chloride (10 mL) was added a solution fo **35** (0.60 g, 2.21 mmol) in methylene chloride (3 mL) dropwise at 0 °C. The mixture was stirred at room temperature overnight and concentrated. The residue was purified by silica gel column chromatography (MeOH:methylene chloride=1:15) to afford **36** (0.43 g, 75%).

2-Methyl-2,4,8,9-tetrahydro-7*H*-cyclopenta[6,7]chromeno[3,4-*c*]pyrazol-7-one (37): To a solution of **36** (1.50 g, 5.77 mmol), PPh_3 (2.82 g, 8.72 mmol) in THF (10 mL) were added DIAD (1.76 g, 8.72 mmol) dropwise under nitrogen. The mixture was stirred at room temperature for 1 h, quenched with water (20 mL), and extracted with ethyl acetate.The organic layers were combined and concentrated. The residue was purified by silica gel column chromatography (EA:PE=1:10) to afford **37** (0.96 g, 69%).

2-Methyl-7-((trimethylsilyl)oxy)-2,7,8,9-tetrahydro-4*H*-cyclopenta[6,7]chromeno [3,4-*c*]pyrazole-7-carbonitrile (38): To a solution of **37** (0.95 g, 3.96 mmol) and NMO (0.14 g, 1.19 mmol) in DMF (20 mL) was added TMSCN (0.79 g, 7.92 mmol) under nitrogen at -20 °C. After completion, the solution was poured into $NaHCO_3$ solution (150 mL) and extracted with ethyl acetate. The organic layers were combined and concentrated to afford crude **37**.

Ethyl 7-hydroxy-2-methyl-2,7,8,9-tetrahydro-4*H*-cyclopenta[6,7]chromeno[3,4-*c*] pyrazole-7-carbimidate (39): Prepared using the same procedure as for **11**, except using ethyl 7-hydroxy-2-methyl-2,7,8,9-tetrahydro-4*H*-cyclopenta [6,7]chromeno [3,4-*c*] pyrazole-7-carbimidate (**38**, 0.025 g) instead of **10** afforded crude **39** (0.025 g).

2-methyl-2,4,8,9-tetrahydrospiro[cyclopenta[6,7]chromeno[3,4-*c*]pyrazole-7,5'-oxazolidine]-2',4'-dione (40): To a solution of **39** (0.025 g, 0.08 mmol) and triethylamine (0.033 g, 0.32 mmol) in THF (2 mL) was added triphosgene (0.01 g, 0.032 mmol) at 0 °C. The mixture was stirred at 10 °C for 1 h. After cooling to 0 °C, 2 N hydrochloric acid (20 mL) was added. The mixture was extracted with ethyl acetate. The organic layers were combined and concentrated to afford **40** (0.01 g, 41%).

Scheme S5

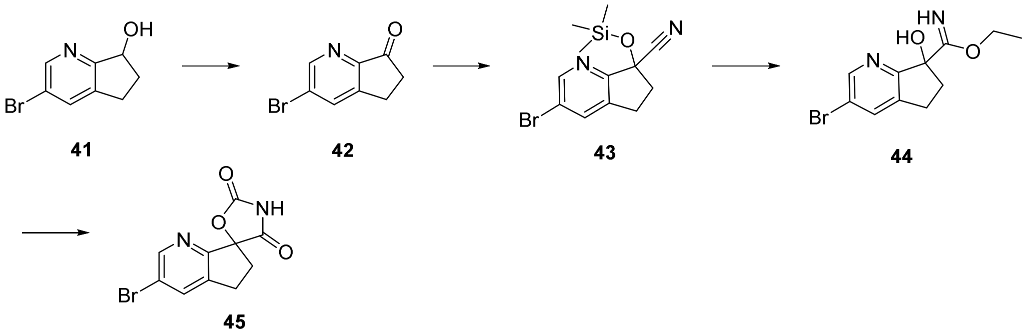

3-Bromo-5,6-dihydro-7*H*-cyclopenta[*b*]pyridin-7-one (42): To a solution of 3-bromo-6,7-dihydro-5*H*-cyclopenta[*b*]pyridin-7-ol (**41**, 1.52 g, 7.08 mmol) in DMSO (10 mL) was added IBX (3.96 g, 14.16 mmol). The mixture was at room temperature for 2 h, quenched with water (70 mL), and extracted with ethyl acetate. The organic layers were combined, washed with brine, and concentrated. The residue was purified by silica gel column chromatography to afford **42** (1.27 g, 85%).

3-Bromo-7-((trimethylsilyl)oxy)-6,7-dihydro-5*H*-cyclopenta[*b*]pyridine-7-carbonitrile (43): Prepared using the same procedure as for **10**, except using 3-bromo-5,6-dihydro-7*H*-cyclopenta[*b*]pyridin-7-one (**42**, 1.27 g, 6.02 mmol) instead of **9** afforded **43** (1.14 g, 61%).

Ethyl 3-bromo-7-hydroxy-6,7-dihydro-5*H*-cyclopenta[*b*]pyridine-7-carbimidate (44): Prepared using the same procedure as for **11**, except using 3-bromo-7-((trimethylsilyl)oxy)-6,7-dihydro-5*H*-cyclopenta[*b*]pyridine-7-carbonitrile (**43**, 1.14 g, 3.67 mmol) instead of **10** afforded **44** (0.7 g, 67%).

3-Bromo-5,6-dihydrospiro[cyclopenta[*b*]pyridine-7,5'-oxazolidine]-2',4'-dione (45): Prepared using the same procedure as for **12**, except using ethyl 3-bromo-7-

hydroxy-6,7-dihydro-5*H*-cyclopenta[*b*]pyridine-7-carbimidate (**44**, 0.7 g, 2.45 mmol) instead of **11** afforded **45** (0.32 g, 47%).

Scheme S6

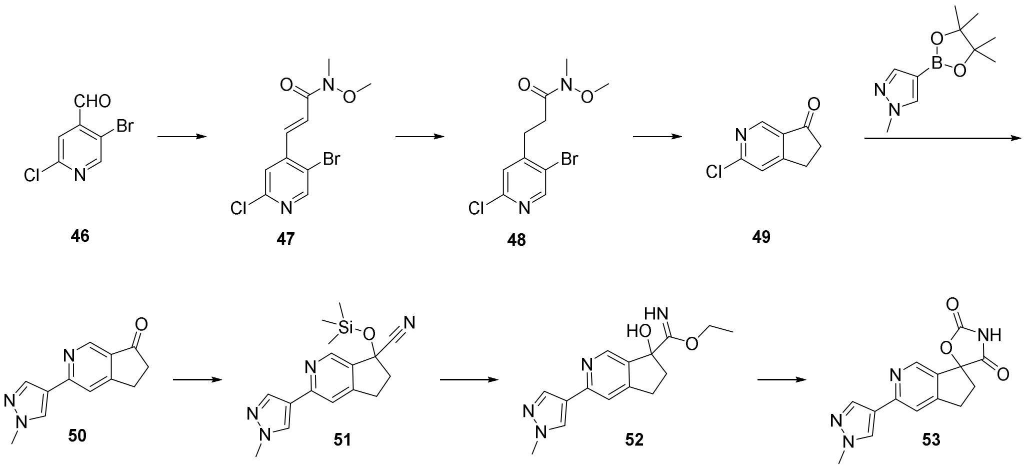

(*E*)-3-(5-bromo-2-chloropyridin-4-yl)-*N*-methoxy-*N*-methylacrylamide (47): A solution of 5-bromo-2-chloroisonicotinaldehyde (**46**, 5.0 g, 22.59 mmol) and *N*-methoxy-*N*-methyl-triphenylphosphor-anylidene (10.0 g) in toluene (30 mL) was stirred at 100 °C for 3 h. After completion, the organic layers were concentrated. The residue was purified by silica gel column chromatography to afford **47** (2.9 g, 42%).

3-(5-Bromo-2-chloropyridin-4-yl)-*N*-methoxy-*N*-methylpropanamide (48): To a solution of (*E*)-3-(5-bromo-2-chloropyridin-4-yl)-*N*-methoxy-*N*-methylacrylamide (**47**, 2.2 g, 7.2 mmol) in EtOAc (70 mL) was added Rh/Al_2CO_3 (0.88 g, 20%, w/w) under a hydrogen atmosphere. The mixture was stirred at 40 °C for 3 h. After completion, the mixture was filtered and the filtrate was concentrated to afford crude product **48** without further purification.

3-Chloro-5,6-dihydro-7*H*-cyclopenta[*c*]pyridin-7-one (49): To a solution of 3-(5-bromo-2-chloropyridin-4-yl)-*N*-methoxy-*N*-methylpropanamide (2 g) in THF (70 mL) was added n-BuLi (3.2 mL, 8.0 mmol) dropwise under nitrogen at -78 °C. The mixture was stirred for 2 h, quenched with saturated NH_4Cl, and extracted with EtOAc. The organic layers were combined and dried over anhydrous Na_2SO_4. The organic layers were concentrated to afford **49** (0.69 g, 57%).

3-(1-Methyl-1*H*-pyrazol-4-yl)-5,6-dihydro-7*H*-cyclopenta[*c*]pyridin-7-one (50): A mixture of 3-chloro-5,6-dihydro-7*H*-cyclopenta[*c*]pyridin-7-one (**49**, 0.69 g, 4.13 mmol), 1-methyl-4-(4,4,5,5-tetramethyl-1,3,2-dioxaborolan-2-yl)-1*H*-pyrazole(1.7 g, 8.26 mmol), Pd(dppf)Cl_2 (0.29 g, 0.41 mmol) and Na_2CO_3 (1.3 g, 12.39 mmol) in 1,4-dioxane/H_2O (10 mL/1 mL) was stirred under nitrogen at 100 °C for 3 h. After

completion, the reaction was quenched with saturated NH_4Cl and extracted with EtOAc. The organic layers were combined and concentrated to afford **50** (0.46 g, 52%).

3-(1-Methyl-1*H*-pyrazol-4-yl)-7-((trimethylsilyl)oxy)-6,7-dihydro-5*H*-cyclopenta-[*c*]pyridine-7-carbonitrile (51): Prepared using the same procedure as for **10**, except using 3-(1-methyl-1*H*-pyrazol-4-yl)-5,6-dihydro-7*H*-cyclopenta [*c*]pyridin-7-one (**50**, 0.46 g, 2.15mmol) instead of **9** afforded **51** (0.42 g, 62%).

Ethyl 7-hydroxy-3-(1-methyl-1*H*-pyrazol-4-yl)-6,7-dihydro-5*H*-cyclopenta[*c*] pyridine-7-carbimidate (52): Prepared using the same procedure as for **11**, except using 3-(1-methyl-1*H*-pyrazol-4-yl)-7-((trimethylsilyl)oxy)-6,7-dihydro-5*H*-cyclo-penta-[*c*]pyridine-7-carbonitrile (**51**, 0.42 g, 1.34 mmol) instead of **10** afforded **52** (0.21 g, 55%).

3-(1-Methyl-1*H*-pyrazol-4-yl)-5,6-dihydrospiro[cyclopenta[*c*]pyridine-7,5'-oxazolidine]-2',4'-dione (53): Prepared using the same procedure as for **12**, except using ethyl 7-hydroxy-3-(1-methyl-1*H*-pyrazol-4-yl)-6,7-dihydro-5*H*-cyclopenta [*c*]pyridine-7-carbimidate (**52**, 0.21 g, 0.74 mmol) instead of **11** afforded **53** (0.12 g, 57%).

Scheme S7

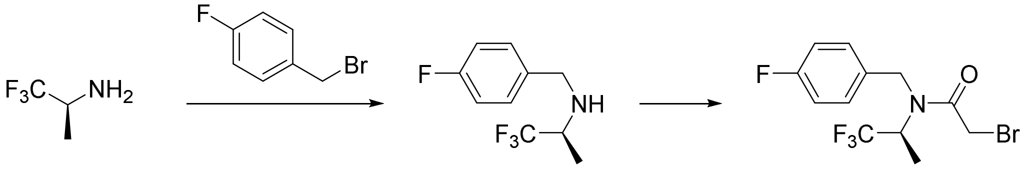

54 **55** **56**

(*S*)-1,1,1-trifluoro-*N*-(4-fluorobenzyl)propan-2-amine (55): A suspension of 1-(bromomethyl)-4-fluorobenzene (8.2 g, 403.3 mmol), (*S*)-1,1,1-trifluoropropan-2-amine (**54**, 5.4 g, 36.11 mmol), and K_2CO_3 (14.9 g, 108.3 mmol) in DMF (50 mL) was stirred at 30 °C overnight. After completion, 1 N HCl (40 mL) was added at 5 °C. The mixture was extracted with ethyl acetate. The organic layers were combined, washed with brine, and concentrated. The residue was purified by silica gel column chromatography (EA:PE=1:5) to afford **55** (6.7 g, 70%).

(*S*)-2-bromo-*N*-(4-fluorobenzyl)-*N*-(1,1,1-trifluoropropan-2-yl)acetamide (56): To a solution of (*S*)-1,1,1-trifluoro-*N*-(4-fluorobenzyl)propan-2-amine (**55**, 5.0 g, 22.6 mmol) in methylene chloride (30 mL) was added dropwise bromoacetyl bromide (9.0 g, 45.2 mmol) at room temperature. The mixture was stirred for 30 minutes, poured into $NaHCO_3$ solution and extracted with ethyl acetate. The organic layers were combined and concentrated. The residue was purified by silica gel column chromatography

(EA:PE=1:10) to afford **56** (7.4 g, 96.1%).

Scheme S8

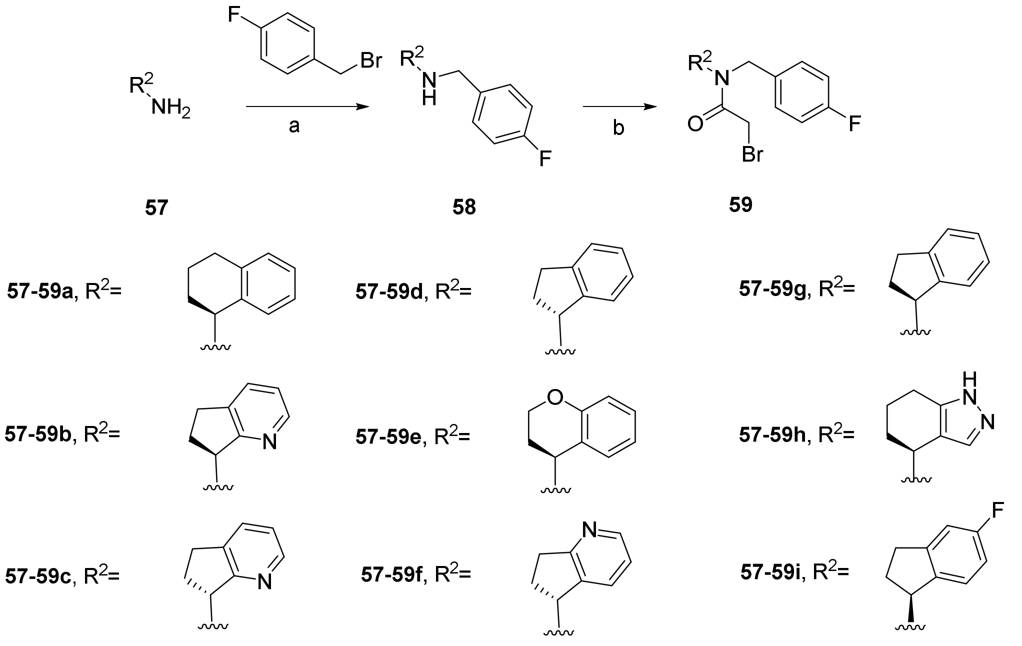

Reagents and conditions: (a) Cs_2CO_3, DMF, r.t., (b) bromoacetyl bromide, TEA, methylene chloride.

(*S*)-*N*-(4-fluorobenzyl)-1,2,3,4-tetrahydronaphthalen-1-amine (58a): A suspension of **57a** (1.0 g, 6.8 mmol), 1-(bromomethyl)-4-fluorobenzene (5.4 g, 36.11 mmol), and Cs_2CO_3 (2.21 g, 6.8 mmol) in DMF (14 mL) was stirred at room temperature for 1 h. After completion, the mixture was poured into NH_4Cl solution, extracted with ethyl acetate. The organic layers were combined, washed with brine, and dried over anhydrous sodium sulfate. After removal of the solvent, the residue was purified by silica gel column chromatography (EA:PE=1:20) to afford **58a** (1.35 g, 78%).

(*S*)-2-bromo-*N*-(4-fluorobenzyl)-*N*-(1,2,3,4-tetrahydronaphthalen-1-yl) acetamide (59a): To a solution of (*S*)-*N*-(4-fluorobenzyl)-1,2,3,4-tetrahydronaphthalen-1-amine (**58a**, 1.35 g, 5.27 mmol) and triethylamine (0.53 g, 5.27 mmol) in methylene chloride (12 mL) was added bromoacetyl bromide (1.06 g, 5.27 mmol) dropwise. After completion, the reaction was quenched with water and extracted with ethyl acetate. The organic layers were combined and dried over anhydrous sodium sulfate. After removal of the solvent, the residue was purified by silica gel column chromatography (EA:PE=1:20) to afford **59a** (1.37 g, 69%).

(*S*)-*N*-(4-fluorobenzyl)chroman-4-amine (58b): Prepared using the same procedure as for **58a**, except using (*S*)-chroman-4-amine (57b, 0.45 g, 3.02 mmol) instead of **57a** afforded **58b** (0.45 g, 58%).

(*S*)-2-bromo-*N*-(chroman-4-yl)-*N*-(4-fluorobenzyl)acetamide (59b): Prepared using the same procedure as for **59a**, except using (*S*)-*N*-(4-fluorobenzyl)chroman-4-amine(**58b**, 0.45 g, 1.75 mmol) instead of **58a** afforded **59b** (0.34 g, 51%).

(*S*)-*N*-(4-fluorobenzyl)-6,7-dihydro-5*H*-cyclopenta[*b*]pyridin-7-amine (58c): Prepared using the same procedure as for **58a**, except using (*S*)-6,7-dihydro-5*H*-cyclopenta[*b*]pyridin-7-amine (**57c**, 0.50 g, 3.73 mmol) instead of **57a** afforded **58c** (0.44 g, 49%).

(*S*)-2-bromo-*N*-(6,7-dihydro-5*H*-cyclopenta[*b*]pyridin-7-yl)-*N*-(4-fluorobenzyl) acetamide (59c): Prepared using the same procedure as for **59a**, except using (*S*)-*N*-(4-fluorobenzyl)-6,7-dihydro-5*H*-cyclopenta[*b*]pyridin-7-amine(**58c**, 0.44 g, 1.82 mmol) instead of **58a** afforded **59c** (0.23 g, 35%).

(*R*)-*N*-(4-fluorobenzyl)-6,7-dihydro-5*H*-cyclopenta[*b*]pyridin-5-amine (58d): Prepared using the same procedure as for **58a**, except using (*R*)-6,7-dihydro-5*H*-cyclopenta[*b*]pyridin-5-amine (**57d**, 0.50 g, 3.73 mmol) instead of **57a** afforded **58d** (0.48 g, 53%).

(*R*)-2-bromo-*N*-(6,7-dihydro-5*H*-cyclopenta[*b*]pyridin-5-yl)-*N*-(4-fluorobenzyl) acet-Amide (59d): Prepared using the same procedure as for **59a**, except using (*R*)-*N*-(4-fluorobenzyl)-6,7-dihydro-5*H*-cyclopenta[*b*]pyridin-5-amine (**58d**, 0.48 g, 1.98 mmol) instead of **58a** afforded **59d** (0.37 g, 51%).

(*R*)-*N*-(4-fluorobenzyl)-6,7-dihydro-5*H*-cyclopenta[*b*]pyridin-7-amine (58e): Prepared using the same procedure as for **58a**, except using (*R*)-6,7-dihydro-5*H*-cyclopenta[*b*]pyridin-7-amine (**57e**, 0.50 g, 3.73 mmol) instead of **57a** afforded **58e** (0.42 g, 47%).

(*R*)-2-bromo-*N*-(6,7-dihydro-5*H*-cyclopenta[*b*]pyridin-7-yl)-*N*-(4-fluorobenzyl) acet-amide (59e): Prepared using with the same procedure as for **59a**, except using (*R*)-*N*-(4-fluorobenzyl)-6,7-dihydro-5*H*-cyclopenta[*b*]pyridin-7-amine (**58e**, 0.42 g, 1.73 mmol) instead of **58a** afforded **59e** (0.24 g, 39%).

(*S*)-*N*-(4-fluorobenzyl)-2,3-dihydro-1*H*-inden-1-amine (58f): Prepared using the same procedure as for **58a**, except using (*S*)-2,3-dihydro-1*H*-inden-1-amine (**57f**, 0.50 g, 3.76 mmol) instead of **57a** afforded **58f** (0.50 g, 55%).

(*S*)-2-bromo-*N*-(2,3-dihydro-1*H*-inden-1-yl)-*N*-(4-fluorobenzyl)acetamide (59f): Prepared using the same procedure as for **59a**, except using (*S*)-*N*-(4-fluorobenzyl)-2,3-dihydro-1*H*-inden-1-amine (**58f**, 0.50 g, 2.07 mmol) instead of **58a** afforded **59f** (0.45 g, 60%).

(*R*)-*N*-(4-fluorobenzyl)-2,3-dihydro-1*H*-inden-1-amine (58g): Prepared using the

same procedure as for **58a**, except using (*R*)-2,3-dihydro-1*H*-inden-1-amine (**57g**, 0.50 g, 3.76 mmol) instead of **57a** afforded **58g** (0.58 g, 64%).

(*R*)-2-bromo-*N*-(2,3-dihydro-1*H*-inden-1-yl)-*N*-(4-fluorobenzyl)acetamide (59g): Prepared using the same procedure as for **59a**, except using (*R*)-*N*-(4-fluorobenzyl)-2,3-dihydro-1*H*-inden-1-amine (**58g**, 0.58 g, 2.41 mmol) instead of **58a** afforded **59g** (0.59 g, 68%).

(*R*)-*N*-(4-fluorobenzyl)-2,3-dihydro-1*H*-inden-1-amine (58h): Prepared using the same procedure as for **58a**, except using (*S*)-1-methyl-4,5,6,7-tetrahydro-1*H*-indazol-4-amine (0.50 g, 3.31 mmol) instead of **57a** afforded **58h** (0.39 g, 46%).

(*R*)-2-bromo-*N*-(2,3-dihydro-1*H*-inden-1-yl)-*N*-(4-fluorobenzyl)acetamide (59h): Prepared using the same procedure as for **59a**, except using (*R*)-*N*-(4-fluorobenzyl)-2,3-dihydro-1*H*-inden-1-amine (**58h**, 0.39 g, 1.51 mmol) instead of **58a** afforded **59h** (0.59 g, 68%).

(*S*)-5-fluoro-*N*-(4-fluorobenzyl)-2,3-dihydro-1*H*-inden-1-amine (58i): Prepared using the same procedure as for **58a**, except using (*S*)-5-fluoro-2,3-dihydro-1*H*-inden-1-amine (0.52 g, 3.49 mmol) instead of **57a** afforded **58h** (0.16 g, 17%).

(*S*)-2-bromo-*N*-(5-fluoro-2,3-dihydro-1*H*-inden-1-yl)-*N*-(4-fluorobenzyl) acetamide (59i): Prepared using the same procedure as for **59a**, except using (*S*)-5-fluoro-*N*-(4-fluorobenzyl)-2,3-dihydro-1*H*-inden-1-amine (**58i**, 0.16 g, 0.62 mmol) instead of **58a** afforded **59i** (0.15 g, 64%).

Scheme S9

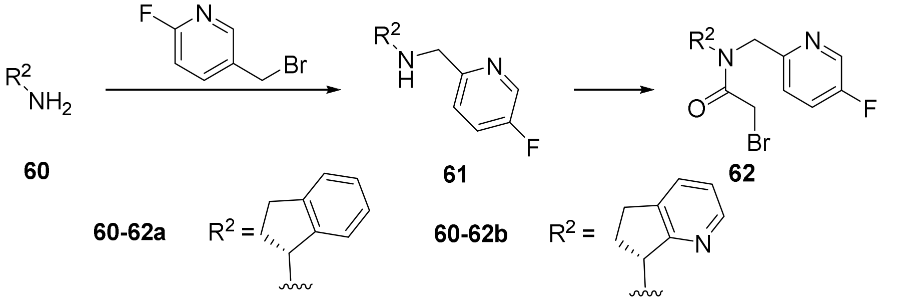

(*R*)-*N*-((5-fluoropyridin-2-yl)methyl)-2,3-dihydro-1*H*-inden-1-amine (61a): Prepared using the same procedure as for **58a**, except using (*R*)-2,3-dihydro-1*H*-inden-1-amine (**60a**, 0.50 g, 3.76 mmol) instead of **57a** afforded **61a** (0.28 g, 31%).

(*R*)-2-bromo-*N*-(2,3-dihydro-1*H*-inden-1-yl)-*N*-((5-fluoropyridin-2-yl)methyl) acet-Amide (62a): Prepared using the same procedure as for **59a**, except using (*R*)-*N*-((5-fluoropyridin-2-yl)methyl)-2,3-dihydro-1*H*-inden-1-amine (**61a**, 0.28 g, 1.16 mmol) instead of **58a** afforded **62a** (0.11 g, 27%).

(*R*)-*N*-((5-fluoropyridin-2-yl)methyl)-6,7-dihydro-5*H*-cyclopenta[*b*]pyridin-7-amine (61b): Prepared using the same procedure as for **58a**, except using (*R*)-6,7-dihydro-5*H*-cyclopenta[*b*]pyridin-7-amine (**60b**, 0.50 g, 3.73 mmol) instead of **57a** afforded **61b** (0.38 g, 42%).

(*R*)-2-bromo-*N*-(6,7-dihydro-5*H*-cyclopenta[*b*]pyridin-7-yl)-*N*-((5-fluoropyridin-2-yl)methyl)acetamide (62b): Prepared using with the same procedure as for **59a**, except using (*R*)-*N*-((5-fluoropyridin-2-yl)methyl)-6,7-dihydro-5*H*-cyclopenta [*b*]pyridin-7-amine (**61b**, 0.38 g, 1.56 mmol) instead of **58a** afforded **62b** (0.35 g, 62%).

Scheme S10

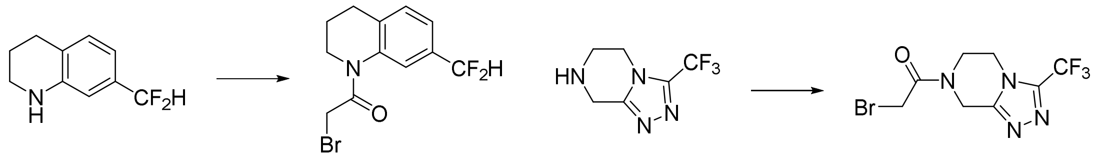

2-Bromo-1-(7-(difluoromethyl)-3,4-dihydroquinolin-1(2*H*)-yl)ethan-1-one (64): To a solution of 7-(difluoromethyl)-1,2,3,4-tetrahydroquinoline (**63**, 0.40 g, 2.19 mmol) and triethylamine (0.66 g, 6.57 mmol) in THF (10 mL) was added bromoacetyl chloride (0.66 g, 3.29 mmol) .The solution was stirred for 1 h at room temperature, quenched with water (20 mL), and extracted with ethyl acetate. The organic layers were combined and dried over anhydrous sodium sulfate. After removal of the solvent, the residue was purified by silica gel column chromatography (EA:PE=1:4) to afford **64** (0.48 g, 73%).

2-Bromo-1-(3-(trifluoromethyl)-5,6-dihydro-[1,2,4]triazolo[4,3-*a*]pyrazin-7(8*H*)-yl)ethan-1-one (66): Prepared using the same procedure as for **56**, except using 3-(trifluoromethyl)-5,6,7,8-tetrahydro-[1,2,4]triazolo[4,3-*a*]pyrazine (**65**, 0.5 g, 2.60 mmol) instead of **55** afforded **66** (0.65 g, 80%).

Scheme S11

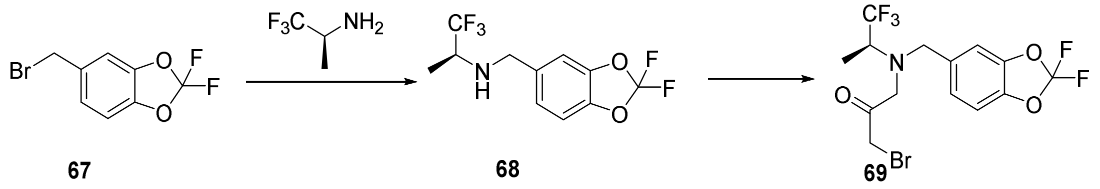

(*S*)-*N*-((2,2-difluorobenzo[*d*][1,3]dioxol-5-yl)methyl)-1,1,1-trifluoropropan-2-amine (68): Prepared using the same procedure as for **55**, except using 5-(bromomethyl)-2,2-difluorobenzo[*d*][1,3]dioxole (**67**, 1 g, 5.20 mmol) instead of **54** afforded **68** (0.48 g, 42%).

(*S*)-1-bromo-3-(((2,2-difluorobenzo[*d*][1,3]dioxol-5-yl)methyl)(1,1,1-trifluoropropan-2-yl)amino)propan-2-one (69): Prepared using the same procedure as for **56**, except using (*S*)-*N*-((2,2-difluorobenzo[*d*][1,3]dioxol-5-yl)methyl)-1,1,1-trifluoropropan-2-amine (**68**, 0.48 g, 1.70 mmol) instead of **55** afforded **69** (0.41 g, 58%).

Scheme S12

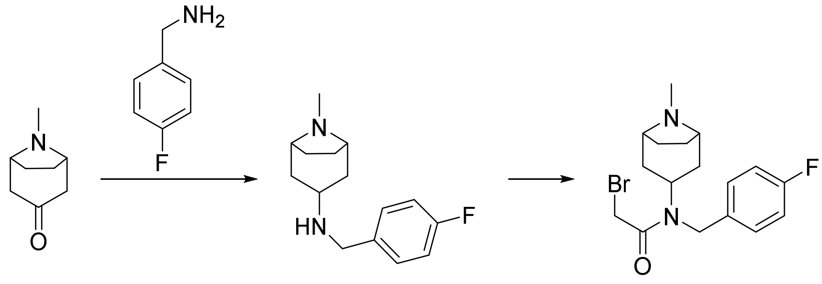

70 **71** **72**

(1*R*,5*S*)-*N*-(4-fluorobenzyl)-8-methyl-8-azabicyclo[3.2.1]octan-3-amine (71): To a solution of 4-fluorobenzylamine (1.0 g, 8 mmol), 8-methyl-8-azabicyclo[3.2.1]octan-3-one (1.34 g, 9.6 mmol) and CF_3COOH (30 ml) in methanol (30 mL) was added sodium cyanoborohydride (3.39 g, 16 mmol). The mixture was stirred at room temperature for 3 h and concentrated. The residue was purified by silica gel column chromatography (MeOH: methylene chloride=1:5, containing 1% triethylamine) to afford **71** (0.78 g, 39%).

2-Bromo-*N*-(4-fluorobenzyl)-*N*-((1*R*,5*S*)-8-methyl-8-azabicyclo[3.2.1]octan-3-yl)acetamide (72): To a solution of (1*R*,5*S*)-*N*-(4-fluorobenzyl)-8-methyl-8-azabicyclo[3.2.1]octan-3-amine (0.5 g, 2.01 mmol) and triethylamine (1.02 g, 10.07 mmol) in DMF (3 mL) and methylene chloride (10 mL) was added and bromoacetyl bromide (1.22 g, 6.04 mmol) at 0 °C. After 1 h, the reaction was quenched with water (20 mL) and extracted with methylene chloride. The organic layers were combined, washed with brine, and dried over anhydrous sodium sulfate. After removal of the solvent, the residue was purified by silica gel column chromatography (MeOH:methylene chloride=1:10) to afford **72** (0.50 g, 68%).

Scheme S13

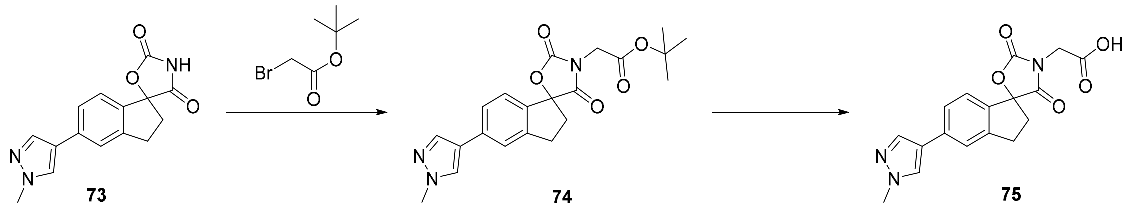

Tert-butyl 2-(5-(1-methyl-1*H*-pyrazol-4-yl)-2',4'-dioxo-2,3-dihydrospiro[indene-1,5'-oxazolidin]-3'-yl)acetate (74): To a solution of 5-(1-methyl-1*H*-pyrazol-4-yl)-2,3-dihydrospiro[indene-1,5'-oxazolidine]-2',4'-dione (1 g, 3.55 mmol) in DMF (10 mL) was added K_2CO_3 (0.98 g, 7.09 mmol). Tert-butyl 2-bromoacetate (0.69 g, 3.55 mmol) was added dropwise. The mixture was stirred at room temperature. After completion, the reaction was quenched with water (50 mL) and extracted with ethyl acetate. The organic layers were combined, washed with brine, and dried over anhydrous sodium sulfate. After removal of the solvent, the residue was purified by silica gel column chromatography (MeOH: methylene chloride=1:10) to afford **74** (0.60 g, 43%).

2-(5-(1-Methyl-1*H*-pyrazol-4-yl)-2',4'-dioxo-2,3-dihydrospiro[indene-1,5'-oxazolidin]-3'-yl)acetic acid (75): A solution of tert-butyl 2-(5-(1-methyl-1*H*-pyrazol-4-yl)-2',4'-dioxo-2,3-dihydrospiro[indene-1,5'-oxazolidin]-3'-yl)acetate (0.6 g) in CF_3COOH (20 mL) was stirred at room temperature for 1 h. The organic layers were concentrated to afford crude product 75 (0.5g, 97%).

Scheme S14

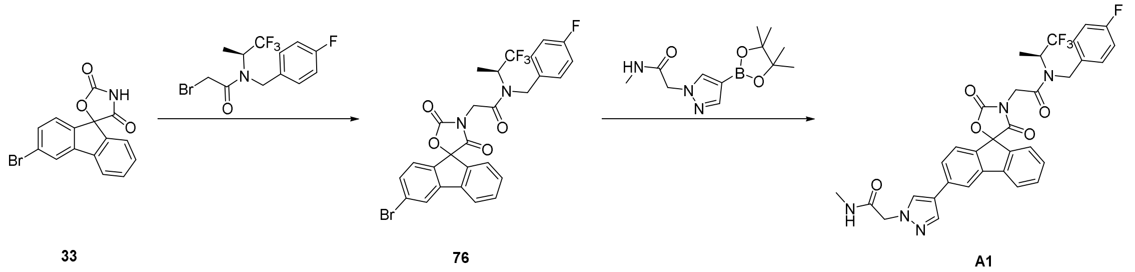

2-(3-bromo-2',4'-dioxospiro[fluorene-9,5'-oxazolidin]-3'-yl)-*N*-(4-fluorobenzyl)-*N*-((*S*)-1,1,1-trifluoropropan-2-yl)acetamide (76): Prepared using the same procedure as for **77**, except using 3-bromospiro[fluorene-9,5'-oxazolidine]-2',4'-dione (**33**, 0.26 g, 0.79 mmol) instead of **28** afforded **76** (0.23 g, 49%).

***N*-(4-fluorobenzyl)-2-(3-(1-(2-(methylamino)-2-oxoethyl)-1*H*-pyrazol-4-yl)-2',4'-dioxospiro[fluorene-9,5'-oxazolidin]-3'-yl)-*N*-((*S*)-1,1,1-trifluoropropan-2-yl)acetamide (A1):** see Manuscript for details.

Scheme S15

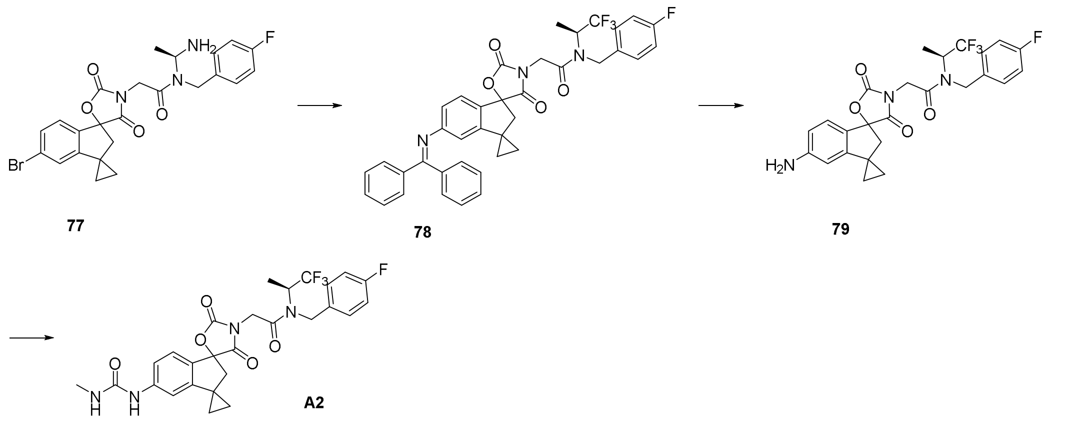

77 78 79 A2

2-(5'-((Diphenylmethylene)amino)-2",4"-dioxo-2'*H*-dispiro[cyclopropane-1,3'-indene-1',5"-oxazolidin]-3"-yl)-*N*-(4-fluorobenzyl)-*N*-((*S*)-1,1,1-trifluoropropan-2-yl)acetamide (78): To a solution of *N*-((*S*)-1-aminoethyl)-2-(5'-bromo-2",4"-dioxo-2'*H*-dispiro[cyclopropane-1,3'-indene-1',5"-oxazolidin]-3"-yl)-*N*-(4-fluorobenzyl) acetamide (**77**, 0.25 g, 0.44 mmol), benzophenone imine (0.104 g, 0.18 mmol), palladium acetate (0.01 g, 0.044 mmol), and 1.1'-binaphthyl-2.2'-diphemyl phosphine (0.11 g, 0.176 mmol) in toluene (4 mL) was added Cs_2CO_3 (0.29 g, 0.88 mmol). The mixture was stirred in 100 °C for 4 h. After completion, the mixture was cooled to room temperature, quenched with water (10 mL) and extracted with ethyl acetate. The organic layers were combined and dried over anhydrous sodium sulfate. After removal of the solvent, the residue was purified by silica gel column chromatography (EA:PE=1:6) to afford **78** (0.26 g, 89%).

2-(5'-Amino-2",4"-dioxo-2'*H*-dispiro[cyclopropane-1,3'-indene-1',5"-oxazolidin]-3"-yl)-*N*-(4-fluorobenzyl)-*N*-((*S*)-1,1,1-trifluoropropan-2-yl)acetamide (79): To a solution of 2-(5'-((diphenylmethylene)amino)-2",4"-dioxo-2'*H*-dispiro[cyclopropane-1,3'-indene-1',5"-oxazolidin]-3"-yl)-*N*-(4-fluorobenzyl)-*N*-((*S*)-1,1,1-trifluoropropan-2-yl)acetamide (**78**, 0.26 g, 0.39 mmol) in THF (4 mL) was added 2 N HCl (0.8 mL). The mixture was stirred at room temperature for 15 minutes. After completion, the reaction was quenched with a saturated solution of sodium bicarbonate (10 mL) and extracted with ethyl acetate. The organic layers were combined and dried over anhydrous sodium sulfate. After removal of the solvent, the residue was purified by silica gel column chromatography (EA:PE=1:3) to afford **79** (0.13 g, 66%).

***N*-(4-fluorobenzyl)-2-(5'-(3-methylureido)-2",4"-dioxo-2'*H*-dispiro[cyclopropane -1,3'-indene-1',5"-oxazolidin]-3"-yl)-*N*-((*S*)-1,1,1-trifluoropropan-2-yl)acetamide (A2):** see Manuscript for details.

Scheme S16

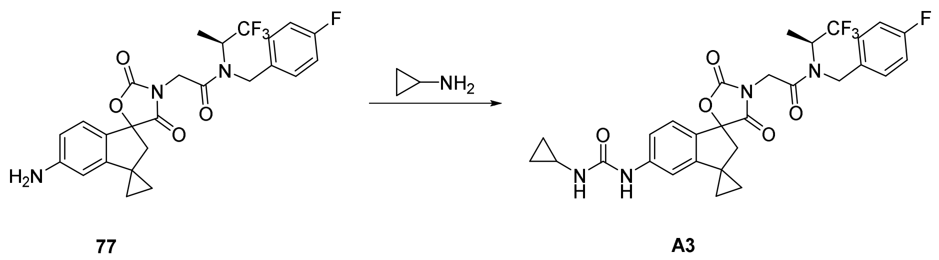

2-(5'-(3-Cyclopropylureido)-2'',4''-dioxo-2'*H*-dispiro[cyclopropane-1,3'-indene-1',5''-oxazolidin]-3''-yl)-*N*-(4-fluorobenzyl)-*N*-((*S*)-1,1,1-trifluoropropan-2-yl)acetamide (A3):** see Manuscript for details.

Scheme S17

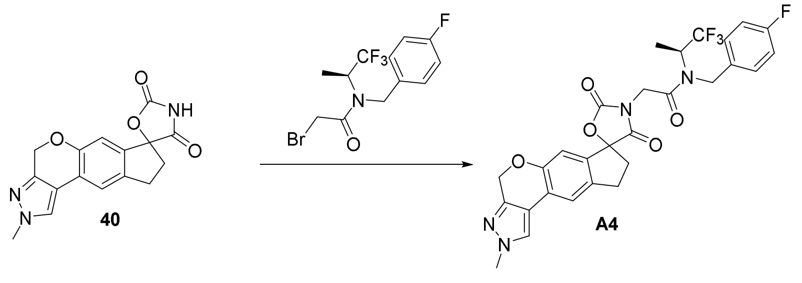

***N*-(4-fluorobenzyl)-2-(2-methyl-2',4'-dioxo-2,4,8,9-tetrahydrospiro [cyclopenta[6,7]chromeno[3,4-*c*]pyrazole-7,5'-oxazolidin]-3'-yl)-*N*-((*S*)-1,1,1-trifluoropropan-2-yl)acetamide (A4):** see Manuscript for details.

Scheme S18

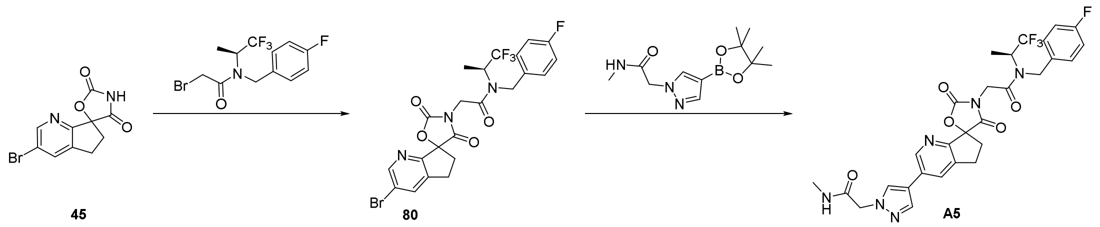

2-(3-Bromo-2',4'-dioxo-5,6-dihydrospiro[cyclopenta[*b*]pyridine-7,5'-oxazolidin]-3'-yl)-*N*-(4-fluorobenzyl)-*N*-((*S*)-1,1,1-trifluoropropan-2-yl)acetamide (80): Prepared using the same procedure as for **77**, except using 3-bromo-5,6-dihydrospiro[cyclopenta[*b*]pyridine-7,5'-oxazolidine]-2',4'-dione (**45**, 0.40 g, 1.42 mmol) instead of **76** afforded **80** (0.27 g, 35%).

***N*-(4-fluorobenzyl)-2-(3-(1-(2-(methylamino)-2-oxoethyl)-1*H*-pyrazol-4-yl)-2',4'-dioxo-5,6-dihydrospiro[cyclopenta[*b*]pyridine-7,5'-oxazolidin]-3'-yl)-*N*-((*S*)-1,1,1-trifluoropropan-2-yl)acetamide (A5):** see Manuscript for details.

Scheme S19

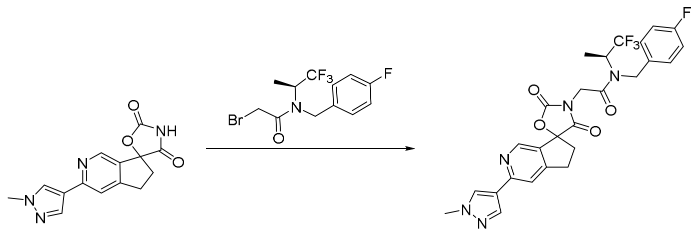

53 A6

***N*-(4-fluorobenzyl)-2-(3-(1-methyl-1*H*-pyrazol-4-yl)-2',4'-dioxo-5,6-dihydrospiro [cyclopenta[*c*]pyridine-7,5'-oxazolidin]-3'-yl)-*N*-((*S*)-1,1,1-trifluoropropan-2-yl) acetamide (A6):** see Manuscript for details.

Scheme S20

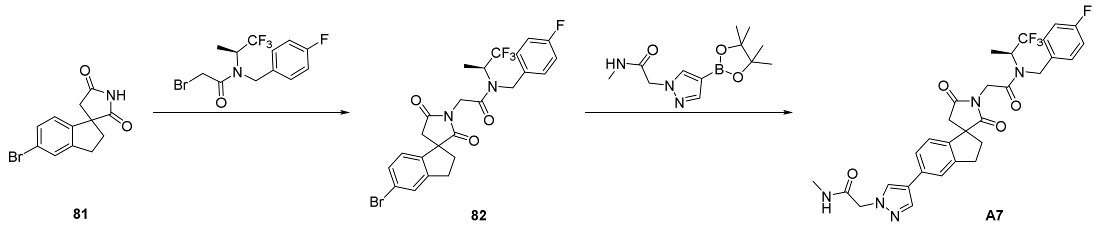

81 82 A7

2-(5-Bromo-2',5'-dioxo-2,3-dihydrospiro[indene-1,3'-pyrrolidin]-1'-yl)-*N*-(4-fluorobenzyl)-*N*-((*S*)-1,1,1-trifluoropropan-2-yl)acetamide (82): Prepared using the same procedure as for **77**, except using 5-bromo-2,3-dihydrospiro[indene-1,3'-pyrrolidine]-2',5'-dione (**81**, 0.50 g, 1.80 mmol) instead of **28** afforded **82** (0.29 g, 30%).
***N*-(4-fluorobenzyl)-2-(5-(1-(2-(methylamino)-2-oxoethyl)-1*H*-pyrazol-4-yl)-2',5'-dioxo-2,3-dihydrospiro[indene-1,3'-pyrrolidin]-1'-yl)-*N*-((*S*)-1,1,1-trifluoropropan-2-yl) acetamide (A7):** see Manuscript for details.

Scheme S21

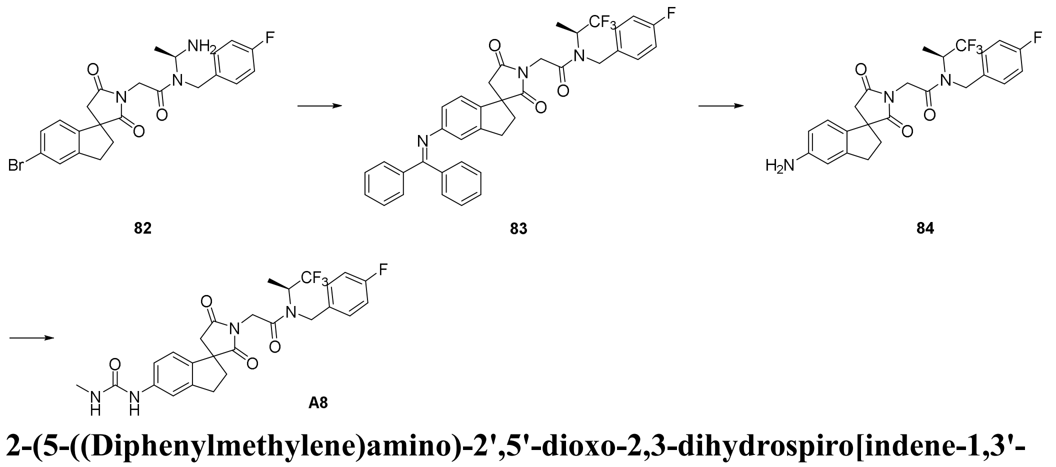

82 83 84

A8

2-(5-((Diphenylmethylene)amino)-2',5'-dioxo-2,3-dihydrospiro[indene-1,3'-pyrrolidin]-1'-yl)-*N*-(4-fluorobenzyl)-*N*-((*S*)-1,1,1-trifluoropropan-2-yl)acetamide

(83): Prepared using the same procedure as for **78**, except using *N*-((*S*)-1-aminoethyl)-2-(5-bromo-2',5'-dioxo-2,3-dihydrospiro[indene-1,3'-pyrrolidin]-1'-yl)-*N*-(4-fluorobenzyl)acetamide (**82**, 0.1 g, 0.21 mmol) instead of **77** afforded **83** (0.11 g, 81%).

2-(5-Amino-2',5'-dioxo-2,3-dihydrospiro[indene-1,3'-pyrrolidin]-1'-yl)-*N*-(4-fluoro-benzyl)-*N*-((*S*)-1,1,1-trifluoropropan-2-yl)acetamide (84): Prepared using the same procedure as for **79**, except using 2-(5-((diphenylmethylene)amino)-2',5'-dioxo-2,3-dihydrospiro[indene-1,3'-pyrrolidin]-1'-yl)-*N*-(4-fluorobenzyl)-*N*-((*S*)-1,1,1-trifluoropropan-2-yl)acetamide (**83**, 0.11 g, 0.17 mmol) instead of **78** afforded **84** (0.04 g, 54%).

***N*-(4-fluorobenzyl)-2-(5-(3-methylureido)-2',5'-dioxo-2,3-dihydrospiro[indene-1,3'-pyrrolidin]-1'-yl)-*N*-((*S*)-1,1,1-trifluoropropan-2-yl)acetamide (A8):** see Manuscript for details.

Scheme S22

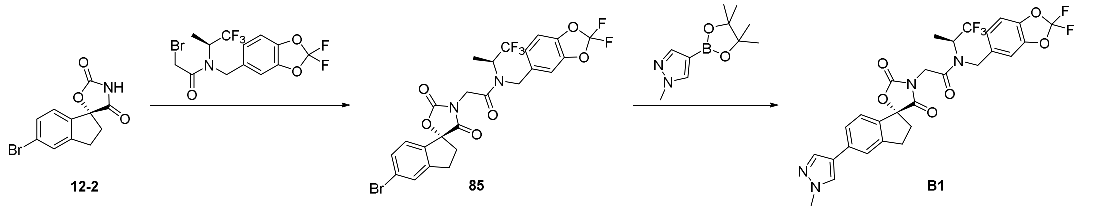

2-((*R*)-5-bromo-2',4'-dioxo-2,3-dihydrospiro[indene-1,5'-oxazolidin]-3'-yl)-*N*-((2,2-difluorobenzo[*d*][1,3]dioxol-5-yl)methyl)-*N*-((*S*)-1,1,1-trifluoropropan-2-yl)acetamide (85): A mixture of (*R*)-5-bromo-2,3-dihydrospiro[indene-1,5'-oxazolidine]-2',4'-dione (**12-2**, 0.25 g, 0.89 mmol), (*S*)-2-bromo-*N*-((2,2-difluorobenzo[*d*][1,3]dioxol-5-yl)methyl)-*N*-(1,1,1-trifluoropropan-2-yl)acetamide (0.36 g, 0.89 mmol) and K_2CO_3 (0.12 g, 0.89 mmol) in DMF (3 mL) was stirred at room temperature for 1 h. After completion, the reaction was quenched with water (15 mL), extracted with ethyl acetate. The organic layers were combined and dried over anhydrous sodium sulfate. After removal of the solvent, the residue was purified by silica gel column chromatography to afford **85** (0.26 g, 49%).

***N*-((2,2-difluorobenzo[*d*][1,3]dioxol-5-yl)methyl)-2-((*R*)-5-(1-methyl-1*H*-pyrazol-4-yl)-2',4'-dioxo-2,3-dihydrospiro[indene-1,5'-oxazolidin]-3'-yl)-*N*-((*S*)-1,1,1-trifluoropropan-2-yl)acetamide (B1):** see Manuscript for details.

Scheme S23

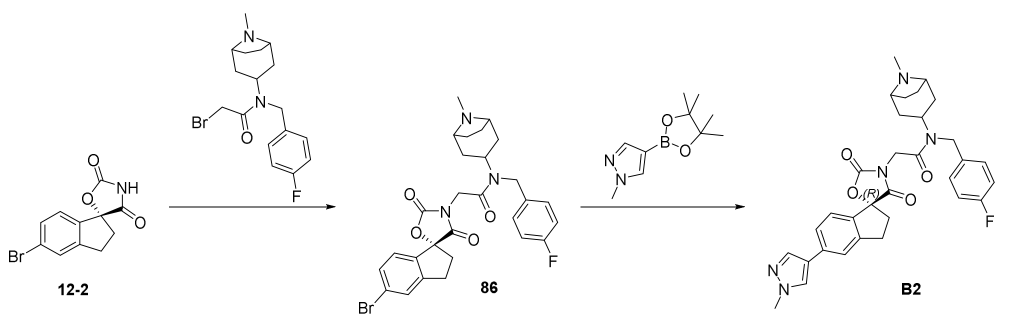

2-((*R*)-5-bromo-2',4'-dioxo-2,3-dihydrospiro[indene-1,5'-oxazolidin]-3'-yl)-*N*-(4-fluorobenzyl)-*N*-(8-methyl-8-azabicyclo[3.2.1]octan-3-yl)acetamide (86): Prepared using the same procedure as for **85**, except using 2-bromo-*N*-(4-fluorobenzyl)-*N*-((1*R*,5*S*)-8-methyl-8-azabicyclo[3.2.1]octan-3-yl)acetamide (0.10 g, 0.27 mmol) instead of (*S*)-2-bromo-*N*-((2,2-difluorobenzo[*d*][1,3]dioxol-5-yl)methyl)-*N*-(1,1,1-trifluoropropan-2-yl)acetamide afforded **86** (0.089 g, 58%).

***N*-(4-fluorobenzyl)-2-((*R*)-5-(1-methyl-1*H*-pyrazol-4-yl)-2',4'-dioxo-2,3-dihydrospiro[indene-1,5'-oxazolidin]-3'-yl)-*N*-(8-methyl-8-azabicyclo[3.2.1]octan-3-yl)acetamide (B2):** see Manuscript for details.

Scheme S24

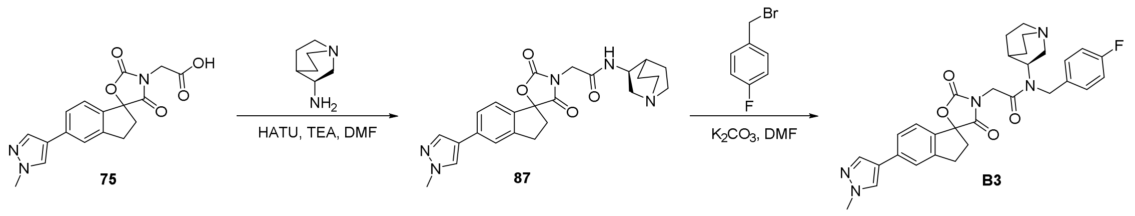

2-(5-(1-Methyl-1*H*-pyrazol-4-yl)-2',4'-dioxo-2,3-dihydrospiro[indene-1,5'-oxazolidin]-3'-yl)-*N*-((*S*)-quinuclidin-3-yl)acetamide (87): To a solution of 2-(5-(1-methyl-1*H*-pyrazol-4-yl)-2',4'-dioxo-2,3-dihydrospiro[indene-1,5'-oxazolidin]-3'-yl)acetic acid (0.34 g, 0.99 mmol), (*S*)-quinuclidin-3-amine (0.2 g, 1.58 mmol) and HATU (0.38 g, 0.99 mmol) in DMF (5 mL) was added triethylamine (0.3 g, 3 mmol). The mixture was stirred at room temperature overnight, quenched with water (50 mL), and extracted with ethyl acetate. The organic layers were combined, washed with brine, and dried over anhydrous sodium sulfate. After removal of the solvent, the residue was purified by silica gel column chromatography (MeOH:methylene chloride=10:1) to afford **87** (0.35 g, 78%).

***N*-(4-fluorobenzyl)-2-(5-(1-methyl-1*H*-pyrazol-4-yl)-2',4'-dioxo-2,3-dihydrospiro [indene-1,5'-oxazolidin]-3'-yl)-*N*-((*S*)-quinuclidin-3-yl)acetamide (B3):** see Manuscript for details.

Scheme S25

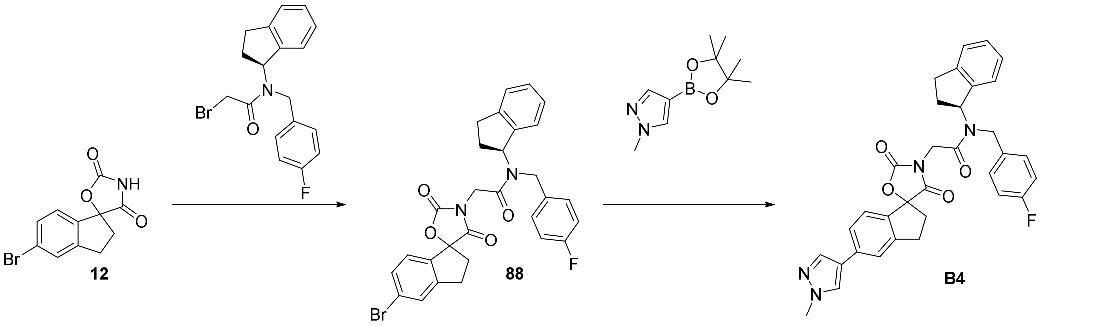

2-(5-Bromo-2',4'-dioxo-2,3-dihydrospiro[indene-1,5'-oxazolidin]-3'-yl)-*N*-((*S*)-2,3-dihydro-1*H*-inden-1-yl)-*N*-(4-fluorobenzyl)acetamide (88): To a solution of 5-bromo-2,3-dihydrospiro[indene-1,5'-oxazolidine]-2',4'-dione (**12**, 0.12 g, 0.41 mmol), (*S*)-2-bromo-*N*-(2,3-dihydro-1*H*-inden-1-yl)-*N*-(4-fluorobenzyl)acetamide (0.11 g, 0.41 mmol) in DMF (2 mL) was added K_2CO_3 (0.50 g, 0.41 mmol). The mixture was stirred at room temperature for 1 h, quenched with water (50 mL), and extracted with ethyl acetate. The organic layers were combined and dried over anhydrous sodium sulfate. After removal of the solvent, the residue was purified by silica gel column chromatography (EA:PE=1:5) to afford **88** (0.15 g, 65%).

***N*-((*S*)-2,3-dihydro-1*H*-inden-1-yl)-*N*-(4-fluorobenzyl)-2-(5-(1-methyl-1*H*-pyrazol-4-yl)-2',4'-dioxo-2,3-dihydrospiro[indene-1,5'-oxazolidin]-3'-yl)acetamide (B4):** see Manuscript for details.

Scheme S26

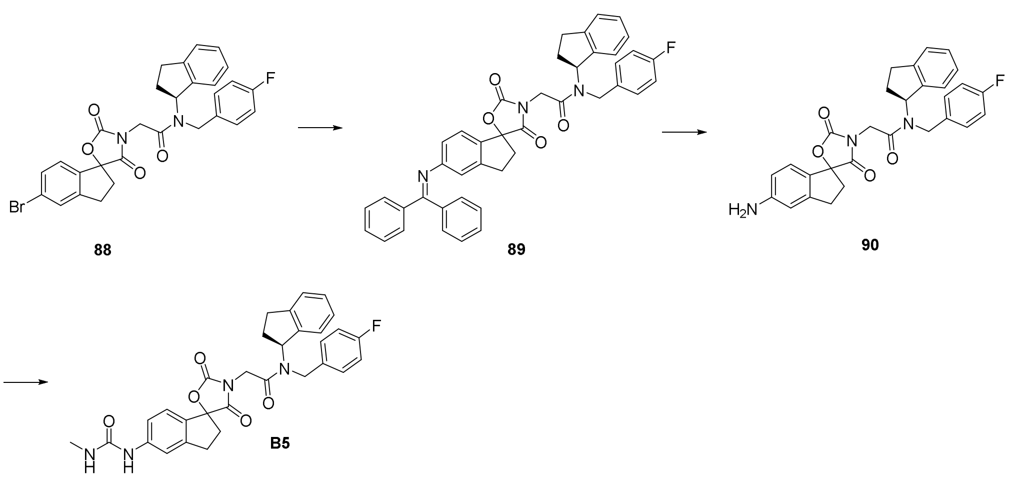

***N*-((*S*)-2,3-dihydro-1*H*-inden-1-yl)-2-(5-((diphenylmethylene)amino)-2',5'-dioxo-2,3-dihydrospiro[indene-1,3'-pyrrolidin]-1'-yl)-*N*-(4-fluorobenzyl)acetamide (89):** Prepared using the same procedure as for **78**, except using 2-(5-bromo-2',5'-dioxo-2,3-

dihydrospiro[indene-1,3'-pyrrolidin]-1'-yl)-*N*-((*S*)-2,3-dihydro-1*H*-inden-1-yl)-*N*-(4-fluorobenzyl)acetamide (**88**, 0.10 g, 0.18 mmol) instead of **77** afforded **89** (0.10 g, 86%).

2-(5-Amino-2',5'-dioxo-2,3-dihydrospiro[indene-1,3'-pyrrolidin]-1'-yl)-*N*-((*S*)-2,3-dihydro-1*H*-inden-1-yl)-*N*-(4-fluorobenzyl)acetamide (90): Prepared using the same procedure as for **79**, except using *N*-((*S*)-2,3-dihydro-1*H*-inden-1-yl)-2-(5-((diphenylmethylene)amino)-2',5'-dioxo-2,3-dihydrospiro[indene-1,3'-pyrrolidin]-1'-yl)-*N*-(4-fluorobenzyl)acetamide (89, 0.10 g) instead of **78** to afford **90** (0.072g, 95.6%).

***N*-((*S*)-2,3-dihydro-1*H*-inden-1-yl)-*N*-(4-fluorobenzyl)-2-(5-(3-methylureido)-2',4'-dioxo-2,3-dihydrospiro[indene-1,5'-oxazolidin]-3'-yl)acetamide (B5):** see Manuscript for details.

Scheme S27

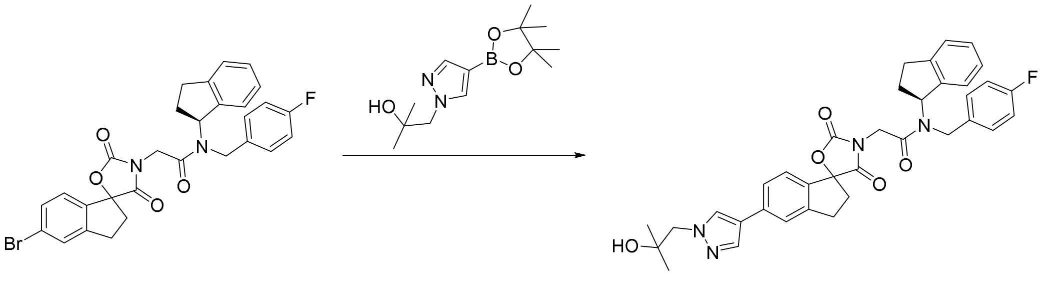

88 **A6**

***N*-((*S*)-2,3-dihydro-1*H*-inden-1-yl)-*N*-(4-fluorobenzyl)-2-(5-(1-(2-hydroxy-2-methylpropyl)-1*H*-pyrazol-4-yl)-2',4'-dioxo-2,3-dihydrospiro[indene-1,5'-oxazolidin]-3'-yl)acetamide (B6):** see Manuscript for details.

Scheme S28

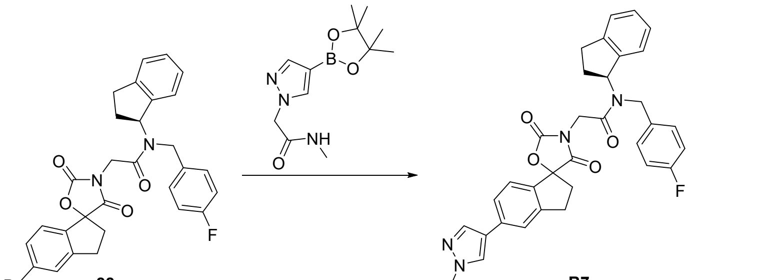

88 **B7**

***N*-((*S*)-2,3-dihydro-1*H*-inden-1-yl)-*N*-(4-fluorobenzyl)-2-(5-(1-(2-(methylamino)-2-oxoethyl)-1*H*-pyrazol-4-yl)-2',4'-dioxo-2,3-dihydrospiro[indene-1,5'-oxazolidin] -3'-yl)acetamide (B7):** see Manuscript for details.

Scheme S29

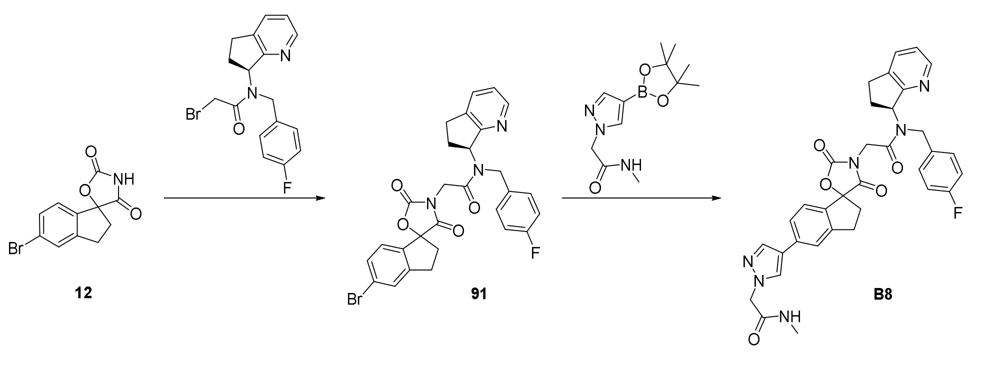

2-(5-bromo-2',4'-dioxo-2,3-dihydrospiro[indene-1,5'-oxazolidin]-3'-yl)-*N*-((*S*)-6,7-dihydro-5*H*-cyclopenta[*b*]pyridin-7-yl)-*N*-(4-fluorobenzyl)acetamide (91): Prepared using the same procedure as for **88**, except using (*S*)-2-bromo-*N*-(6,7-dihydro-5*H*-cyclopenta[*b*]pyridin-7-yl)-*N*-(4-fluorobenzyl)acetamide (0.07 g, 0.20 mmol) instead of (*S*)-2-bromo-*N*-(2,3-dihydro-1*H*-inden-1-yl)-*N*-(4-fluorobenzyl)acetamide afforded **91** (0.078 g, 72%).

***N*-((*S*)-6,7-dihydro-5*H*-cyclopenta[*b*]pyridin-7-yl)-*N*-(4-fluorobenzyl)-2-(5-(1-(2-(methylamino)-2-oxoethyl)-1*H*-pyrazol-4-yl)-2',4'-dioxo-2,3-dihydrospiro[indene-1,5'-oxazolidin]-3'-yl)acetamide (B8):** see Manuscript for details.

Scheme S30

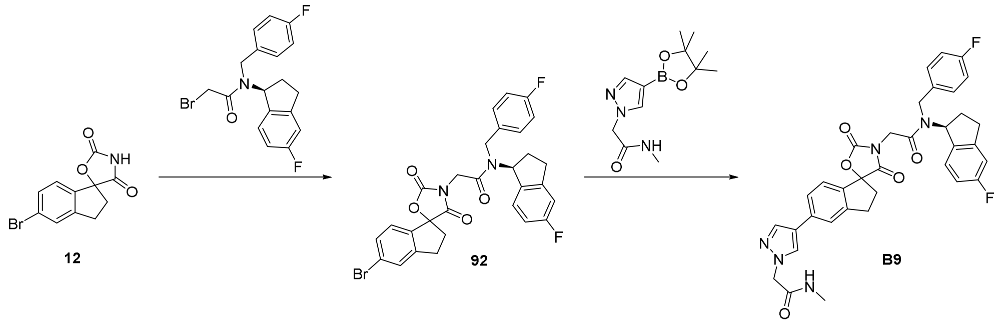

2-(5-Bromo-2',4'-dioxo-2,3-dihydrospiro[indene-1,5'-oxazolidin]-3'-yl)-*N*-((*S*)-5-fluoro-2,3-dihydro-1*H*-inden-1-yl)-*N*-(4-fluorobenzyl)acetamide (92): Prepared using the same procedure as for **88**, except using (*S*)-2-bromo-*N*-(5-fluoro-2,3-dihydro-1*H*-inden-1-yl)-*N*-(4-fluorobenzyl)acetamide (0.69 g, 1.81 mmol) instead of (*S*)-2-bromo-*N*-(2,3-dihydro-1*H*-inden-1-yl)-*N*-(4-fluorobenzyl)acetamide afforded **92** (0.22 g, 55%).

***N*-((*S*)-5-fluoro-2,3-dihydro-1*H*-inden-1-yl)-*N*-(4-fluorobenzyl)-2-(5-(1-(2-(methylamino)-2-oxoethyl)-1*H*-pyrazol-4-yl)-2',4'-dioxo-2,3-dihydrospiro[indene-1,5'-oxazolidin]-3'-yl)acetamide (B9):** see Manuscript for details.

Scheme S31

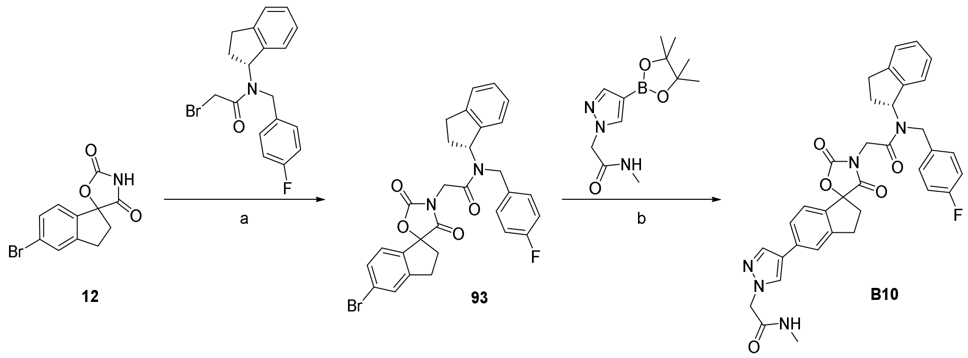

2-(5-bromo-2',4'-dioxo-2,3-dihydrospiro[indene-1,5'-oxazolidin]-3'-yl)-*N*-((*R*)-2,3-dihydro-1*H*-inden-1-yl)-*N*-(4-fluorobenzyl)acetamide (93): Prepared using the same procedure as for **88**, except using (*R*)-2-bromo-*N*-(2,3-dihydro-1*H*-inden-1-yl)-*N*-(4-fluorobenzyl)acetamide (0.12 g, 0.32 mmol) instead of (*S*)-2-bromo-*N*-(2,3-dihydro-1*H*-inden-1-yl)-*N*-(4-fluorobenzyl)acetamide afforded **93** (0.10 g, 55%).

***N*-((*R*)-2,3-dihydro-1*H*-inden-1-yl)-*N*-(4-fluorobenzyl)-2-(5-(1-(2-(methylamino)-2-oxoethyl)-1*H*-pyrazol-4-yl)-2',4'-dioxo-2,3-dihydrospiro[indene-1,5'-oxazolidin]-3'-yl)acetamide (B10):** see Manuscript for details.

Scheme S32

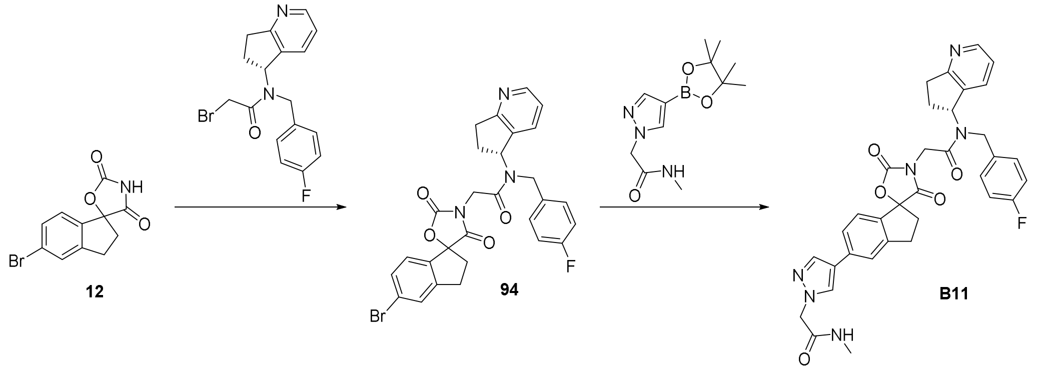

2-(5-Bromo-2',4'-dioxo-2,3-dihydrospiro[indene-1,5'-oxazolidin]-3'-yl)-*N*-((*R*)-6,7-dihydro-5*H*-cyclopenta[*b*]pyridin-5-yl)-*N*-(4-fluorobenzyl)acetamide (94): Prepared using the same procedure as for **88**, except using (*R*)-2-bromo-*N*-(6,7-dihydro-5*H*-cyclopenta[*b*]pyridin-5-yl)-*N*-(4-fluorobenzyl)acetamide (0.20 g, 0.55 mmol) instead of (*S*)-2-bromo-*N*-(2,3-dihydro-1*H*-inden-1-yl)-*N*-(4-fluorobenzyl) acetamide afforded **94** (0.17 g, 56%).

***N*-((*R*)-6,7-dihydro-5*H*-cyclopenta[*b*]pyridin-5-yl)-*N*-(4-fluorobenzyl)-2-(5-(1-(2-(methylamino)-2-oxoethyl)-1*H*-pyrazol-4-yl)-2',4'-dioxo-2,3-dihydrospiro[indene-1,5'-oxazolidin]-3'-yl)acetamide (B11):** see Manuscript for details.

Scheme S33

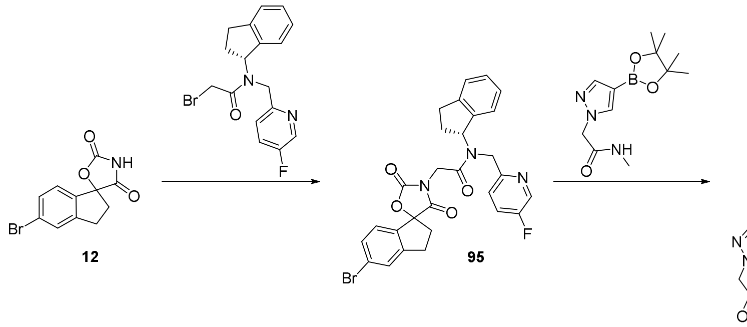

2-(5-Bromo-2',4'-dioxo-2,3-dihydrospiro[indene-1,5'-oxazolidin]-3'-yl)-*N*-((*R*)-2,3-dihydro-1*H*-inden-1-yl)-*N*-((5-fluoropyridin-2-yl)methyl)acetamide (95): Prepared using the same procedure as for **88**, except using (*R*)-2-bromo-*N*-(2,3-dihydro-1*H*-inden-1-yl)-*N*-((5-fluoropyridin-2-yl)methyl)acetamide (0.10 g, 0.28 mmol) instead of (*S*)-2-bromo-*N*-(2,3-dihydro-1*H*-inden-1-yl)-*N*-(4-fluorobenzyl)acetamide afforded **95** (0.11 g, 73%).

***N*-((*R*)-2,3-dihydro-1*H*-inden-1-yl)-*N*-((5-fluoropyridin-2-yl)methyl)-2-(5-(1-(2-(methylamino)-2-oxoethyl)-1*H*-pyrazol-4-yl)-2',4'-dioxo-2,3-dihydrospiro[indene-1,5'-oxazolidin]-3'-yl)acetamide (B12):** see Manuscript for details.

Scheme S34

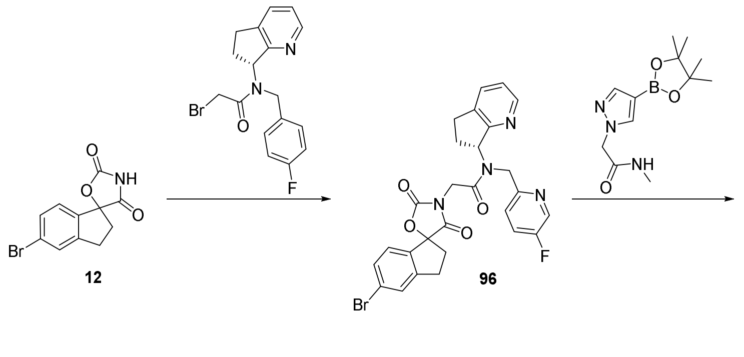

2-(5-Bromo-2',4'-dioxo-2,3-dihydrospiro[indene-1,5'-oxazolidin]-3'-yl)-*N*-((*R*)-6,7-dihydro-5*H*-cyclopenta[*b*]pyridin-7-yl)-*N*-((5-fluoropyridin-2-yl)methyl) acetamide (96): Prepared using the same procedure as for **88**, except using (*R*)-2-bromo-*N*-(6,7-dihydro-5*H*-cyclopenta[*b*]pyridin-7-yl)-*N*-(4-fluorobenzyl)acetamide (0.20 g, 0.55 mmol) instead of (*S*)-2-bromo-*N*-(2,3-dihydro-1*H*-inden-1-yl)-*N*-(4-fluorobenzyl)acetamide afforded **96** (0.20 g, 65%).

***N*-((*R*)-6,7-dihydro-5*H*-cyclopenta[*b*]pyridin-7-yl)-*N*-((5-fluoropyridin-2-yl) methyl)-2-(5-(1-(2-(methylamino)-2-oxoethyl)-1*H*-pyrazol-4-yl)-2',4'-dioxo-2,3-dihydrospiro[indene-1,5'-oxazolidin]-3'-yl)acetamide (B13):** see Manuscript for details.

Scheme S35

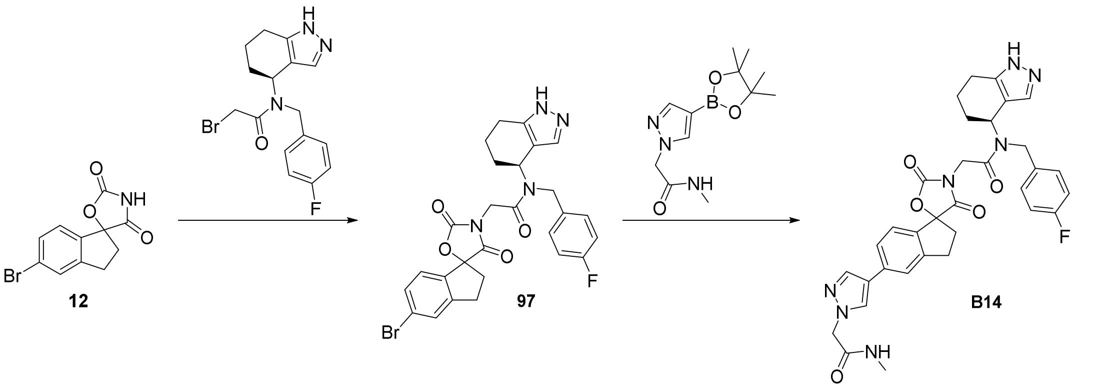

2-(5-Bromo-2',4'-dioxo-2,3-dihydrospiro[indene-1,5'-oxazolidin]-3'-yl)-*N*-(4-fluorobenzyl)-*N*-((*S*)-1-methyl-4,5,6,7-tetrahydro-1*H*-indazol-4-yl)acetamide (97): Prepared using the same procedure as for **88**, except using (*S*)-2-bromo-*N*-(4-fluorobenzyl)-*N*-(1-methyl-4,5,6,7-tetrahydro-1*H*-indazol-4-yl)acetamide (0.17 g, 0.49 mmol) instead of (*S*)-2-bromo-*N*-(2,3-dihydro-1*H*-inden-1-yl)-*N*-(4-fluorobenzyl) acetamide afforded **97** (0.15 g, 59%).

***N*-(4-fluorobenzyl)-*N*-((*S*)-1-methyl-4,5,6,7-tetrahydro-1*H*-indazol-4-yl)-2-(5-(1-(2-(methylamino)-2-oxoethyl)-1*H*-pyrazol-4-yl)-2',4'-dioxo-2,3-dihydrospiro [indene-1,5'-oxazolidin]-3'-yl)acetamide (B14):** see Manuscript for details.

Scheme S36

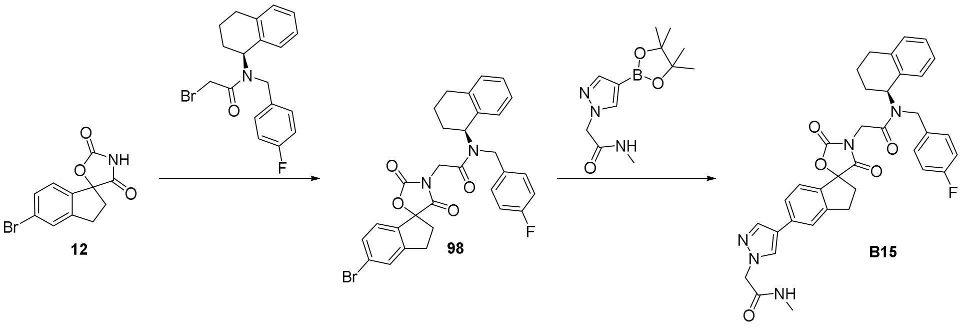

2-(5-Bromo-2',4'-dioxo-2,3-dihydrospiro[indene-1,5'-oxazolidin]-3'-yl)-*N*-(4-fluorobenzyl)-*N*-((*S*)-1,2,3,4-tetrahydronaphthalen-1-yl)acetamide (98): To a solution of 5-bromo-2,3-dihydrospiro[indene-1,5'-oxazolidine]-2',4'-dione (**12**, 1.03 g, 3.65 mmol), (*S*)-2-bromo-*N*-(4-fluorobenzyl)-*N*-(1,2,3,4-tetrahydronaphthalen-1-yl)acetamide (1.37 g, 3.65 mmol) in DMF (5 mL) was added K_2CO_3 (0.50 g, 3.65 mmol). The mixture was stirred at room temperature for 1 h, quenched with water (50 mL), and extracted with ethyl acetate. The organic layers were combined and dried over anhydrous sodium sulfate. After removal of the solvent, the residue was purified by silica gel column chromatography (EA:PE=1:5) to afford **98** (1.45 g, 69%).

N-(4-fluorobenzyl)-2-(5-(1-(2-(methylamino)-2-oxoethyl)-1H-pyrazol-4-yl)-2',4'-dioxo-2,3-dihydrospiro[indene-1,5'-oxazolidin]-3'-yl)-N-((S)-1,2,3,4-tetrahydronaphthalen-1-yl)acetamide (B15): see Manuscript for details.

Scheme S37

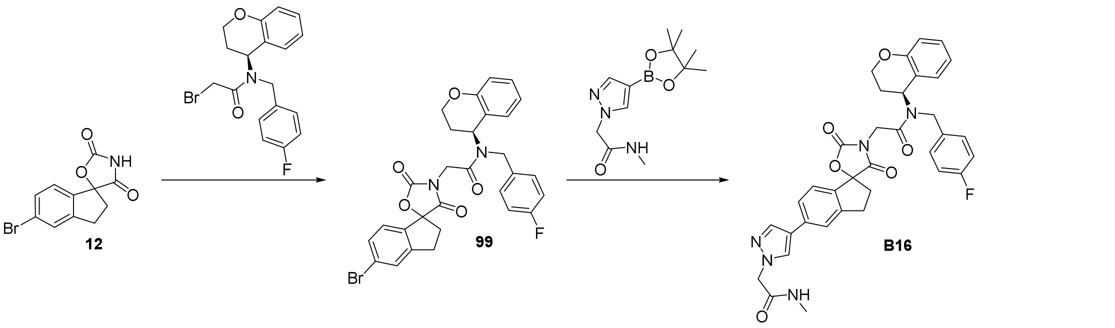

2-(5-bromo-2',4'-dioxo-2,3-dihydrospiro[indene-1,5'-oxazolidin]-3'-yl)-N-((S)-chroman-4-yl)-N-(4-fluorobenzyl)acetamide (99): Prepared using the same procedure as for **98**, except using (*S*)-2-bromo-*N*-(chroman-4-yl)-*N*-(4-fluorobenzyl)acetamide (0.35 g, 1.25 mmol) instead of (*S*)-2-bromo-*N*-(4-fluorobenzyl)-*N*-(1,2,3,4-tetrahydronaphthalen-1-yl)acetamide afforded **99** (0.30 g, 42%).

N-((S)-chroman-4-yl)-N-(4-fluorobenzyl)-2-(5-(1-(2-(methylamino)-2-oxoethyl)-1H-pyrazol-4-yl)-2',4'-dioxo-2,3-dihydrospiro[indene-1,5'-oxazolidin]-3'-yl) acetamide (B16): see Manuscript for details.

Scheme S38

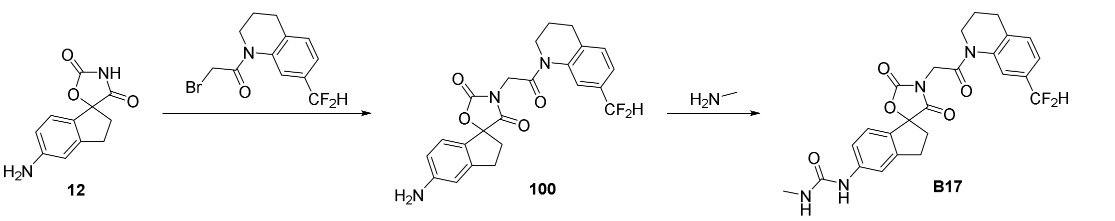

5-Amino-3'-(2-(7-(difluoromethyl)-3,4-dihydroquinolin-1(2H)-yl)-2-oxoethyl)-2,3-dihydrospiro[indene-1,5'-oxazolidine]-2',4'-dione (100): To a solution of 5-amino-2,3-dihydrospiro [indene-1,5'-oxazolidine]-2',4'-dione (**12**, 0.15 g, 0.495 mmol), 2-bromo-1-(7-(difluoromethyl)-3,4-dihydroquinolin-1(2*H*)-yl)ethan-1-one (0.072 g, 0.33 mmol) in DMF (5 mL) was added K_2CO_3 (0.14 g, 0.99 mmol). The mixture was stirred at room temperature for 2 h, quenched with water (50 mL), and extracted with ethyl acetate. The organic layers were combined and dried over anhydrous sodium

sulfate. After removal of the solvent, the residue was purified by silica gel column chromatography (EA:PE=1:1) to afford **100** (0.12 g, 83%).

1-(3'-(2-(7-(Difluoromethyl)-3,4-dihydroquinolin-1(2*H*)-yl)-2-oxoethyl)-2',4'-dioxo-2,3-dihydrospiro[indene-1,5'-oxazolidin]-5-yl)-3-methylurea (B17): see Manuscript for details.

Scheme S39

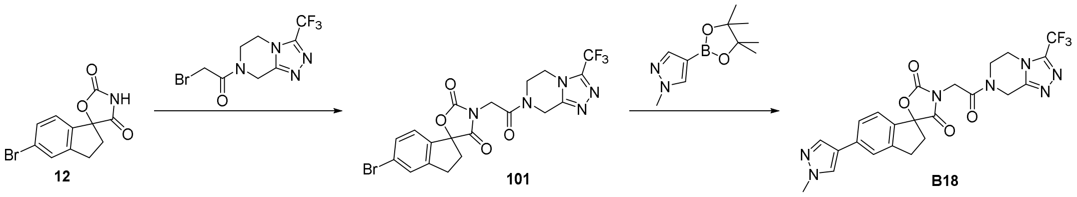

5-Bromo-3'-(2-oxo-2-(3-(trifluoromethyl)-5,6-dihydro-[1,2,4]triazolo[4,3-a]pyrazin-7(8*H*)-yl)ethyl)-2,3-dihydrospiro[indene-1,5'-oxazolidine]-2',4'-dione (101): Prepared using the same procedure as for **100**, except using 2-bromo-1-(3-(trifluoromethyl)-5,6-dihydro-[1,2,4]triazolo[4,3-*a*]pyrazin-7(8*H*)-yl)ethan-1-one (0.11 g, 0.35 mmol) instead of 2-bromo-1-(7-(difluoromethyl)-3,4-dihydroquinolin-1(2*H*)-yl)ethan-1-one afforded **101** (0.10 g, 58%).

5-(1-Methyl-1*H*-pyrazol-4-yl)-3'-(2-oxo-2-(3-(trifluoromethyl)-5,6-dihydro-[1,2,4]triazolo[4,3-*a*]pyrazin-7(8*H*)-yl)ethyl)-2,3-dihydrospiro[indene-1,5'-oxazolidine]-2',4'-dione (B18): see Manuscript for details.

Chemical spectrum and purity determination

^{1}H NMR spectrum of compound **A1**

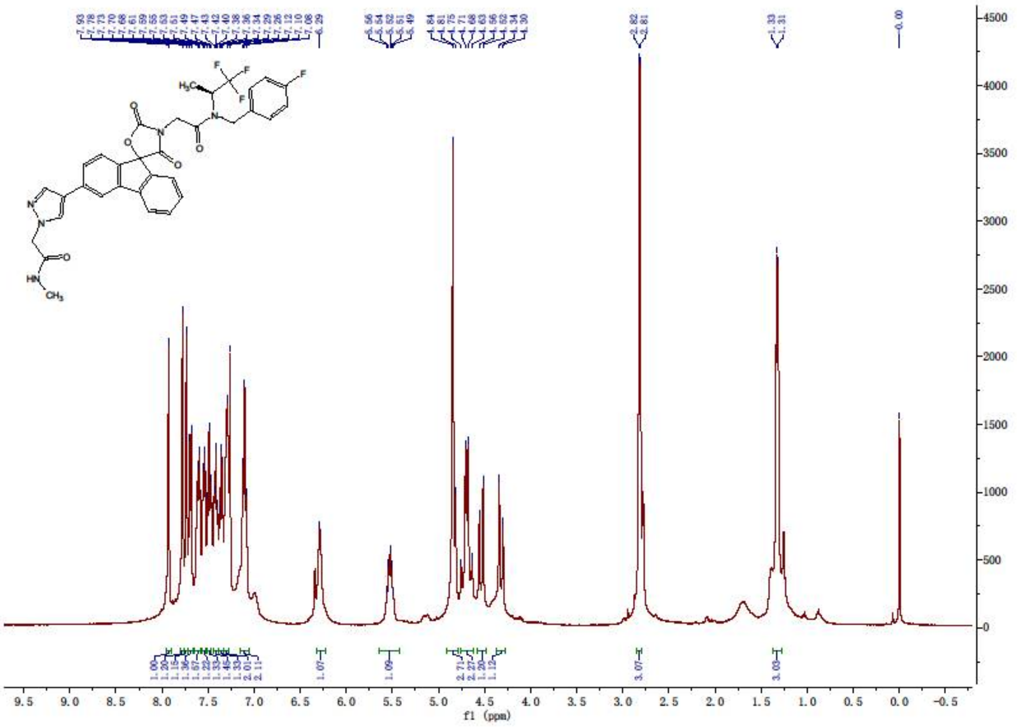

^{13}C NMR spectrum of compound **A1**

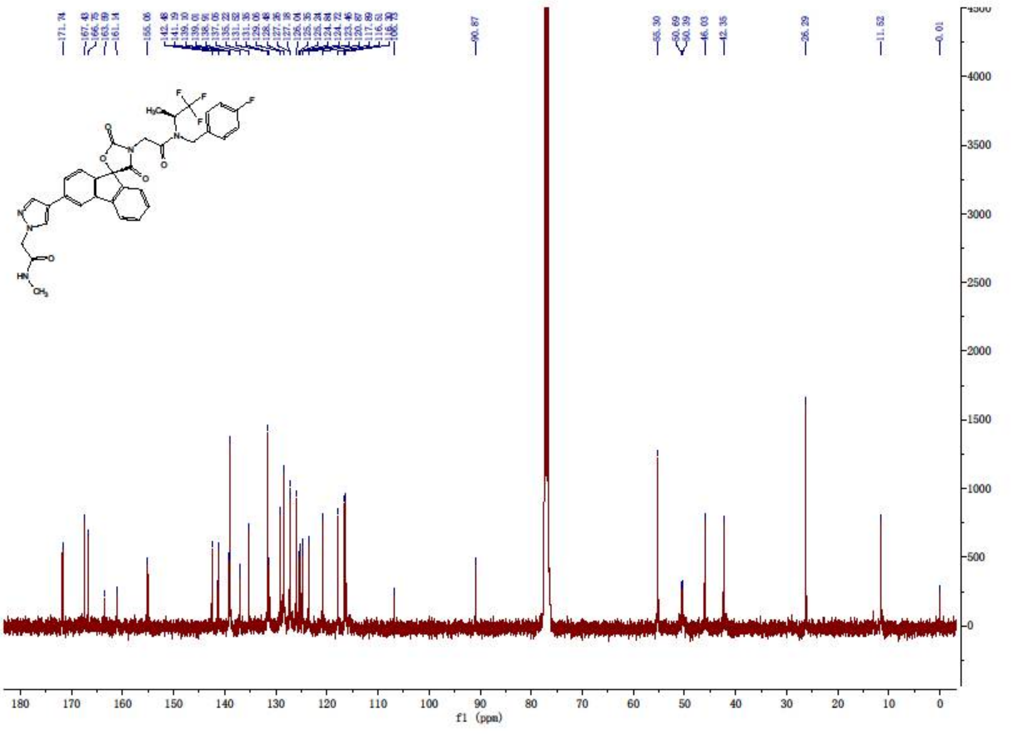

^{1}H NMR spectrum of compound **A2**

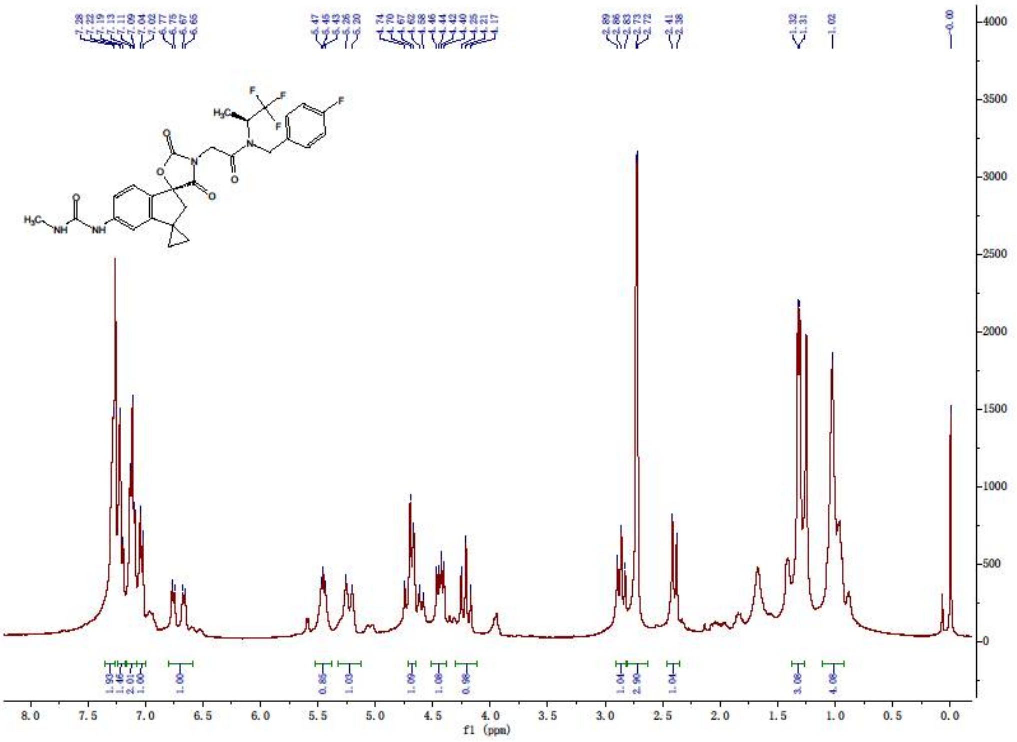

^{13}C NMR spectrum of compound **A2**

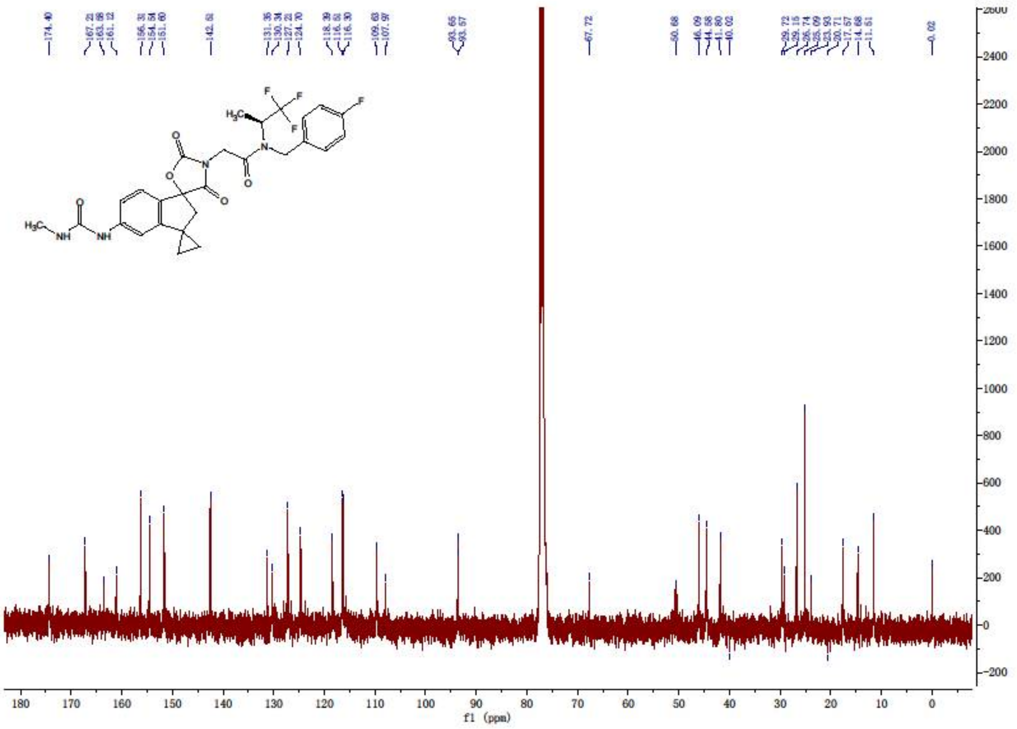

^{1}H NMR spectrum of compound **A3**

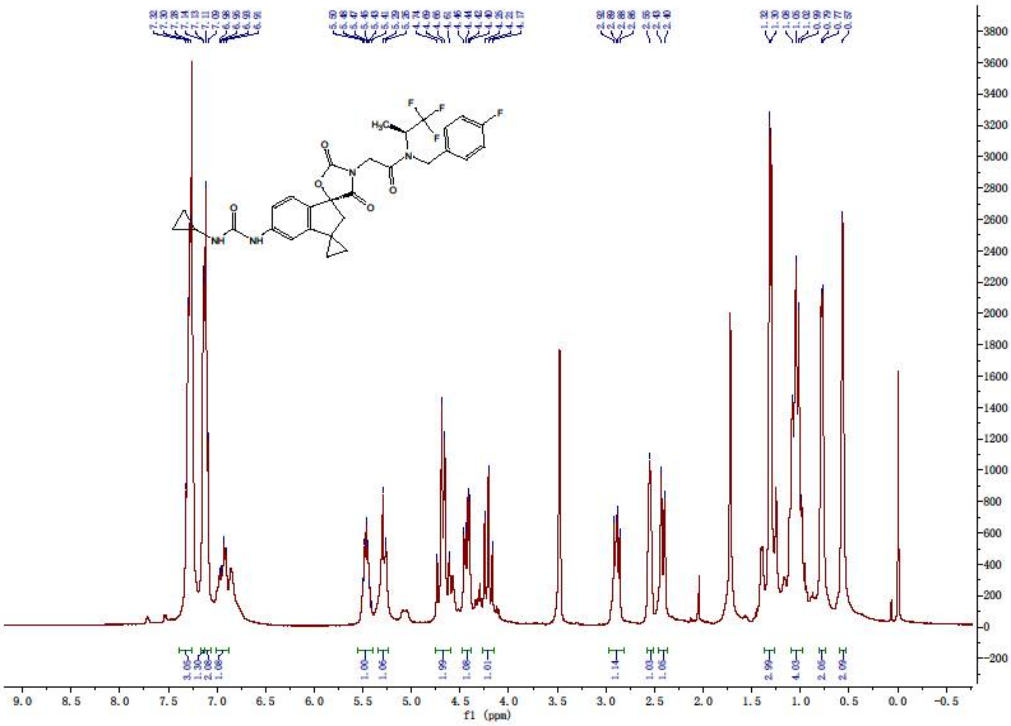

^{13}C NMR spectrum of compound **A3**

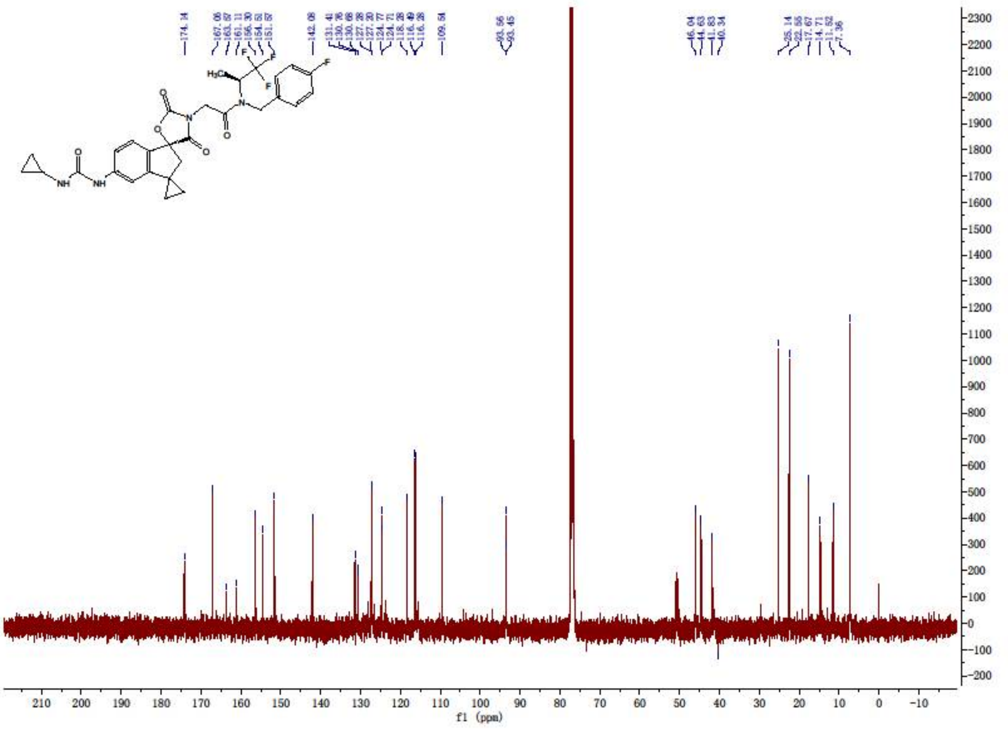

^{1}H NMR spectrum of compound **A4**

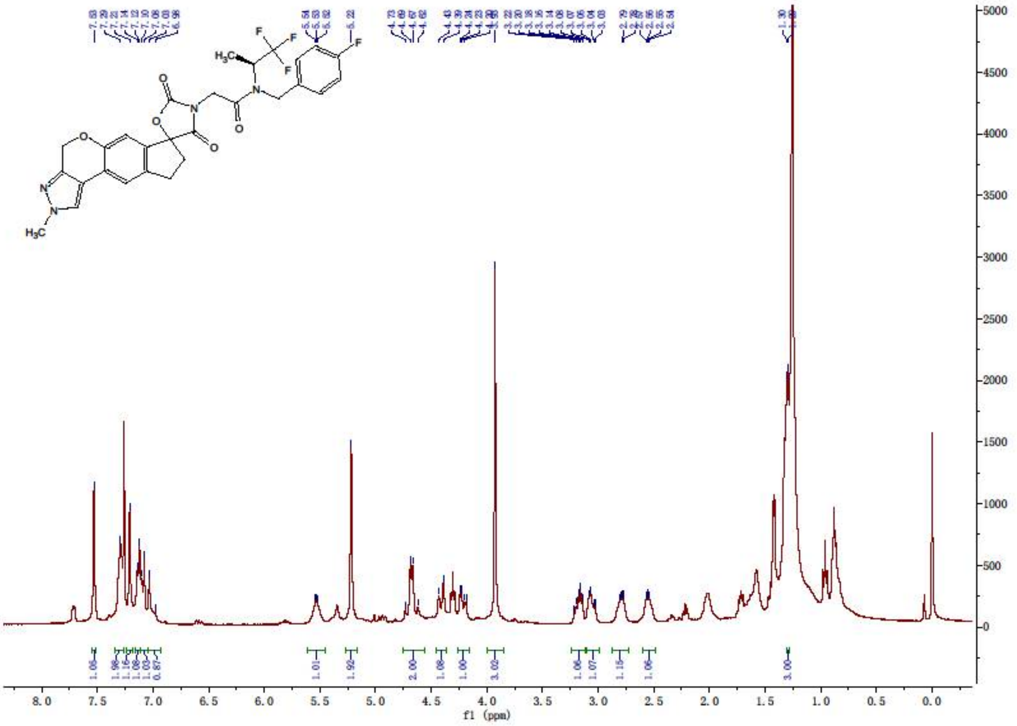

^{13}C NMR spectrum of compound **A4**

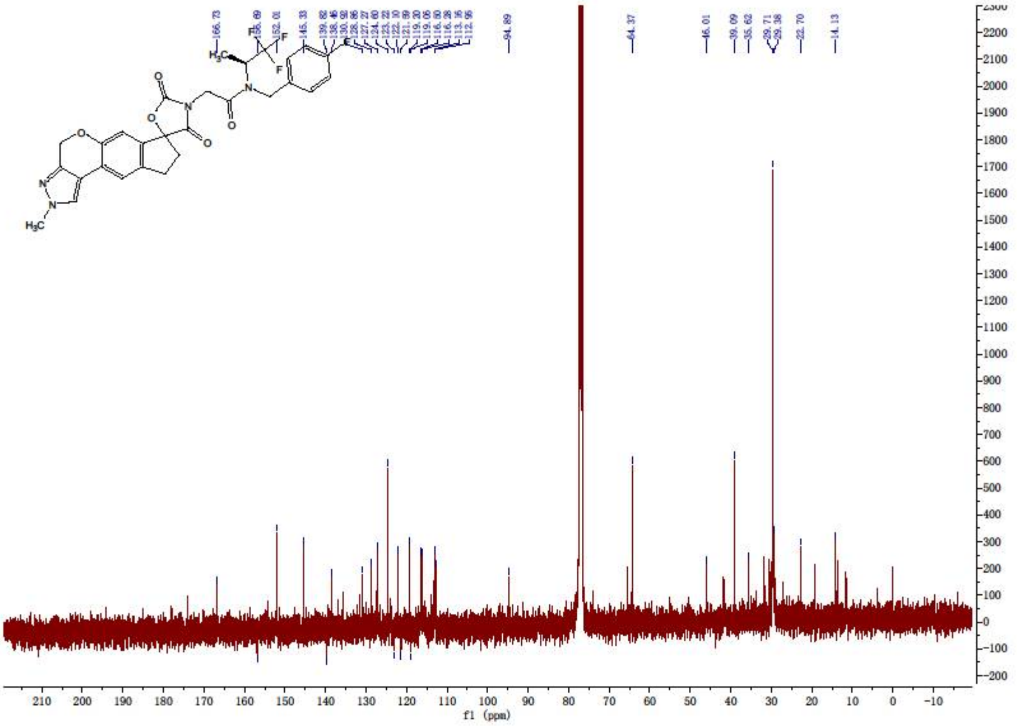

^{1}H NMR spectrum of compound **A5**

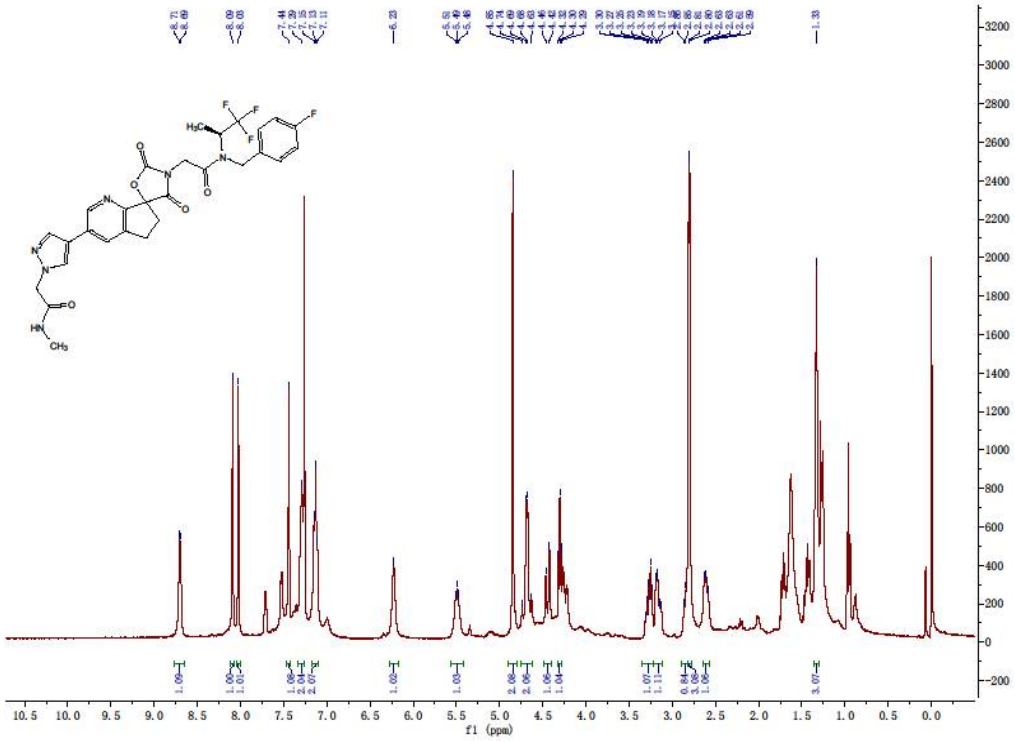

^{13}C NMR spectrum of compound **A5**

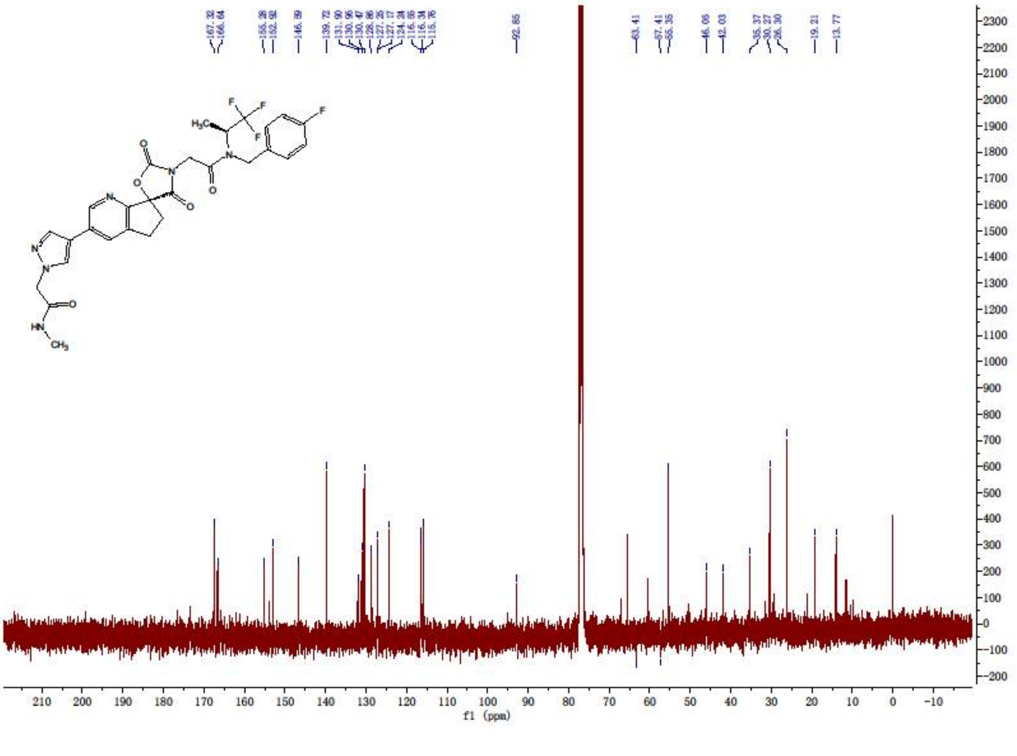

^{1}H NMR spectrum of compound **A6**

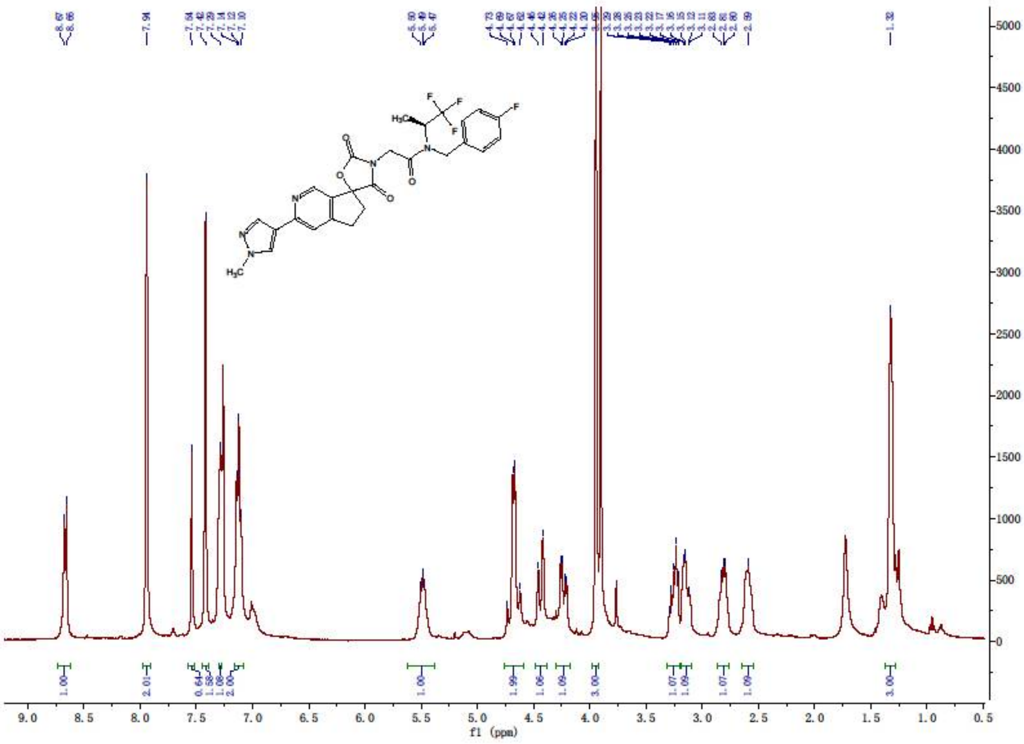

^{13}C NMR spectrum of compound **A6**

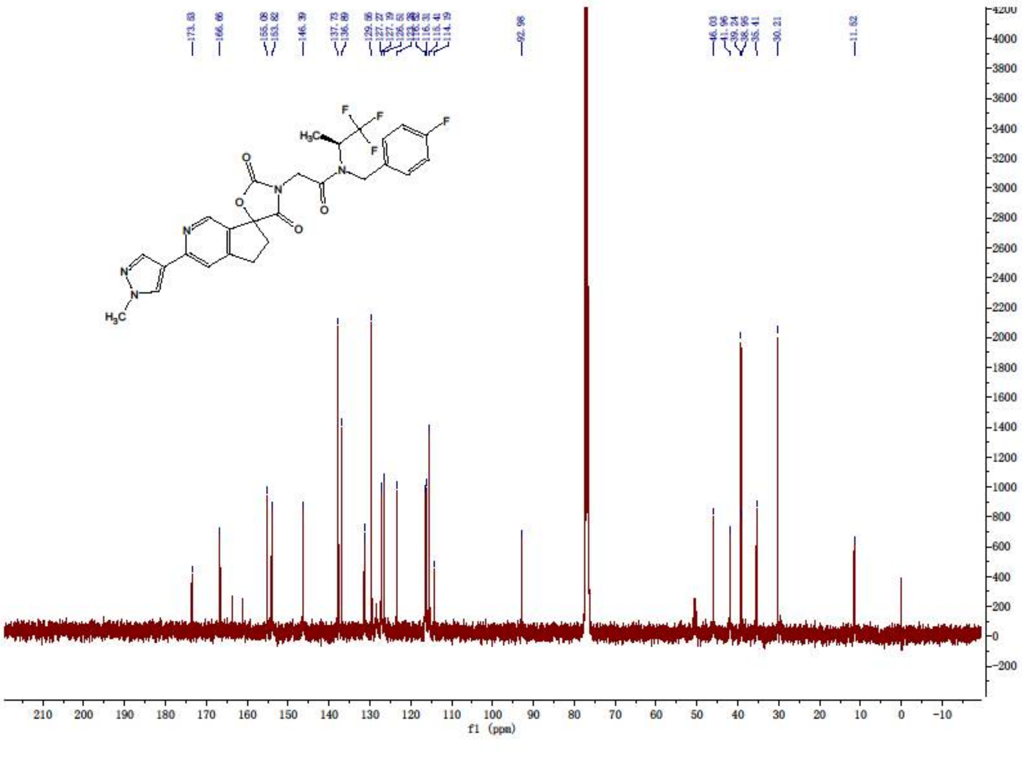

^{1}H NMR spectrum of compound **A7**

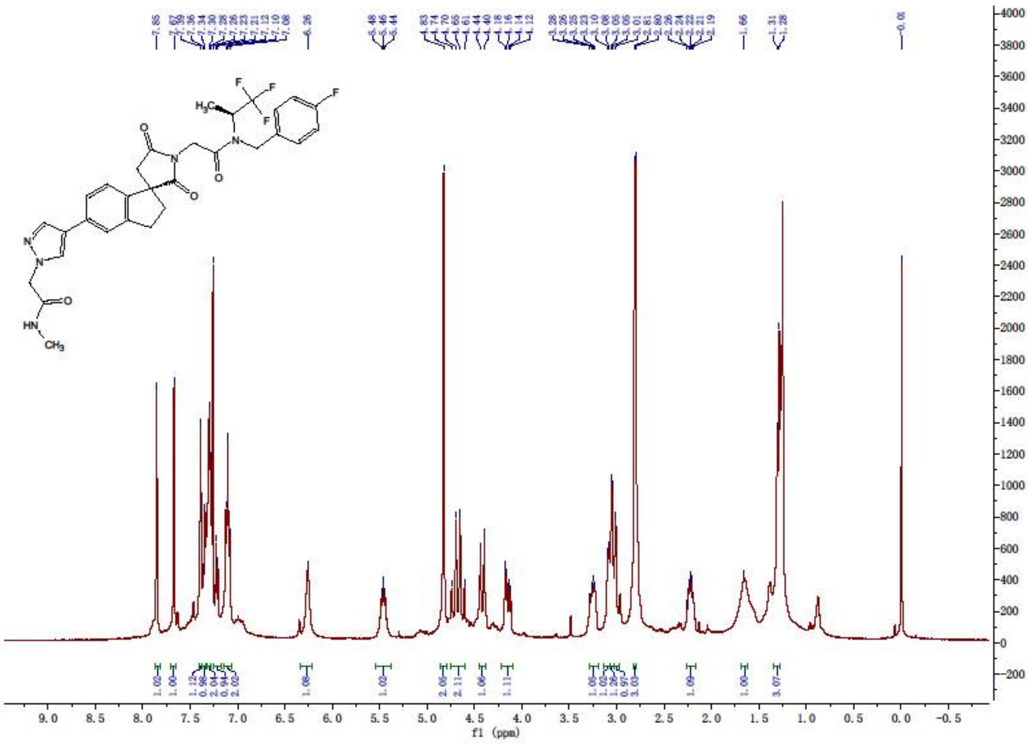

^{13}C NMR spectrum of compound **A7**

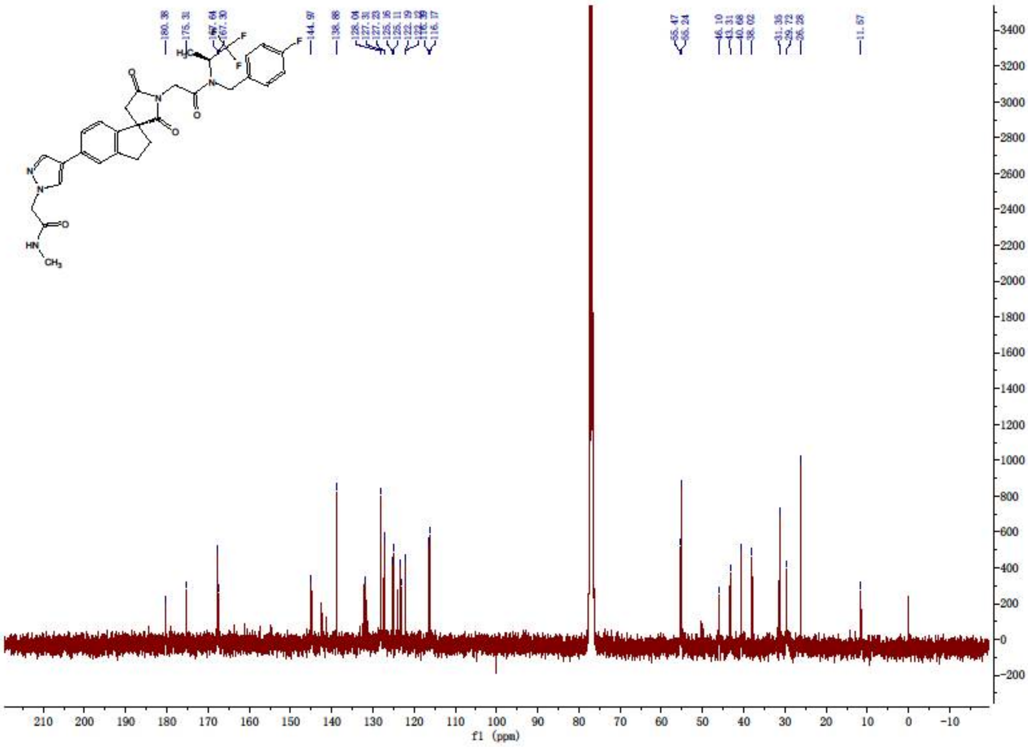

^{1}H NMR spectrum of compound **A8**

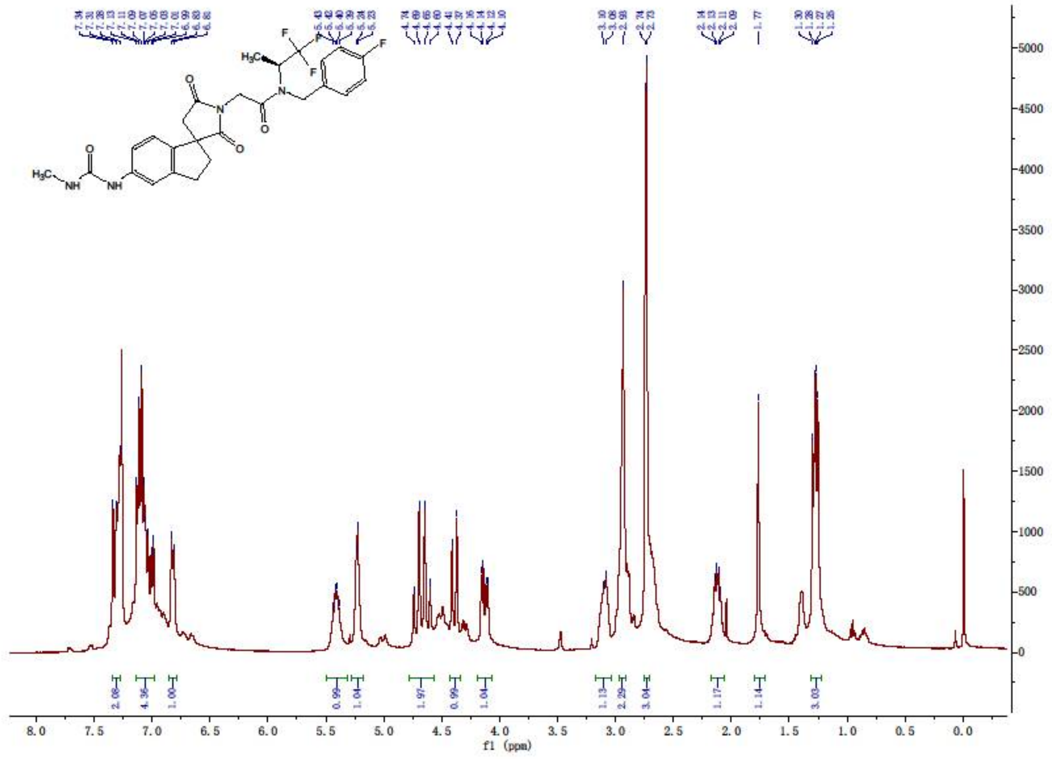

^{13}C NMR spectrum of compound **A8**

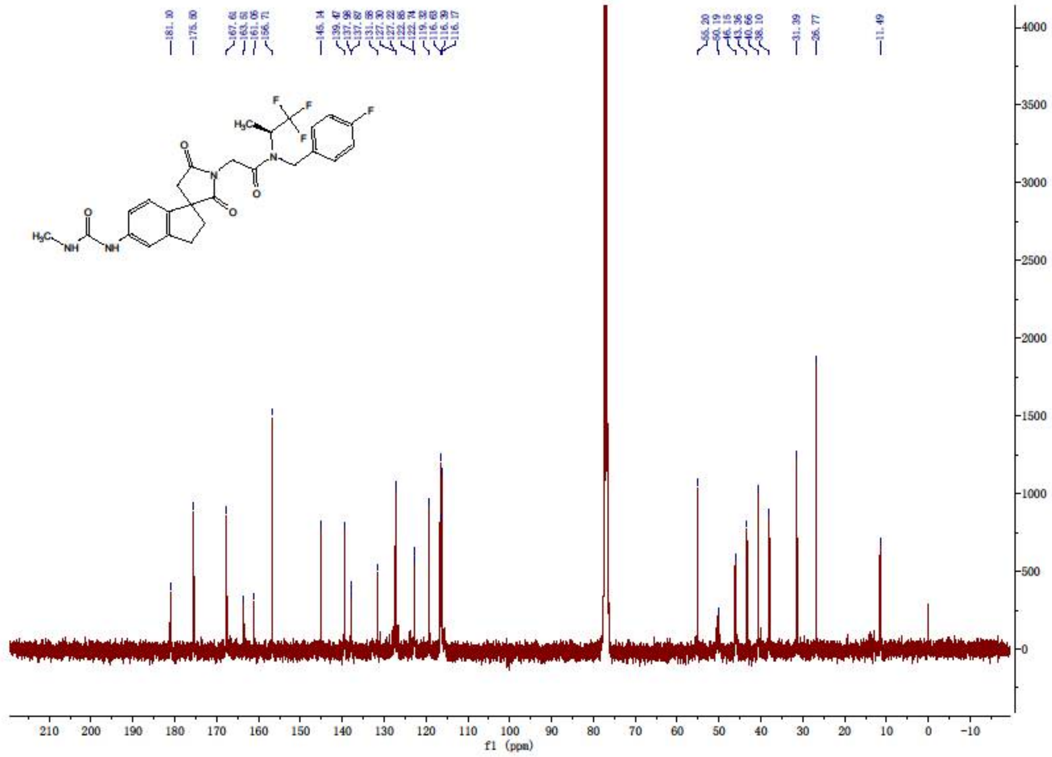

^{1}H NMR spectrum of compound **B1**

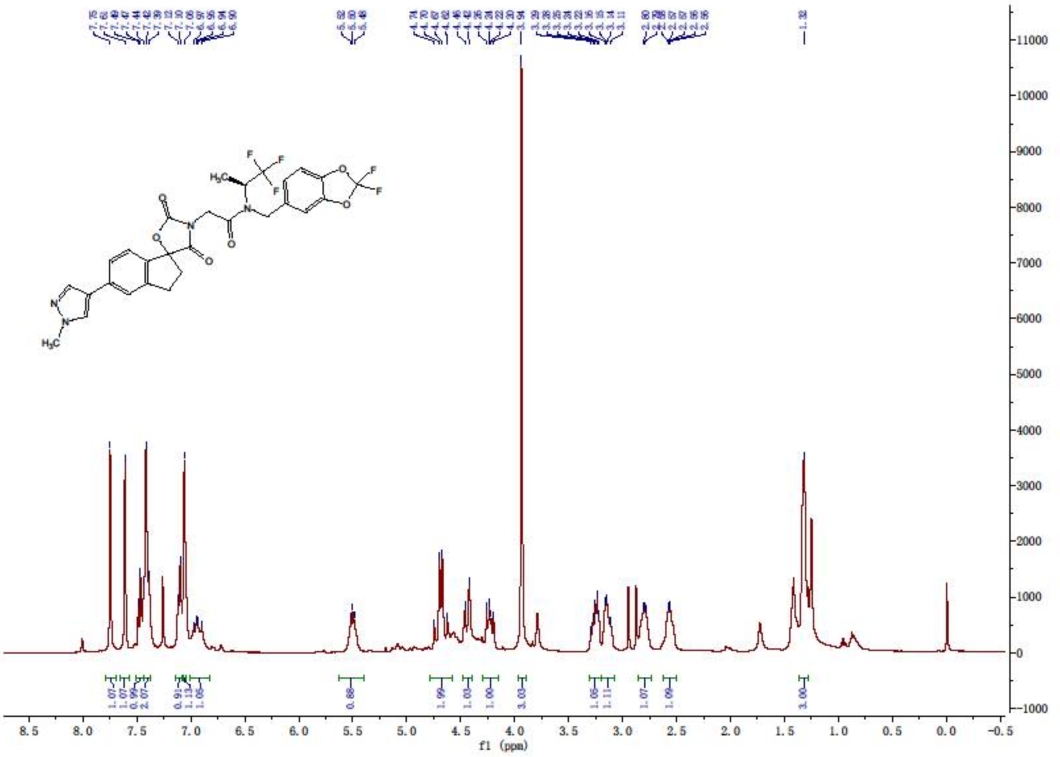

^{13}C NMR spectrum of compound **B1**

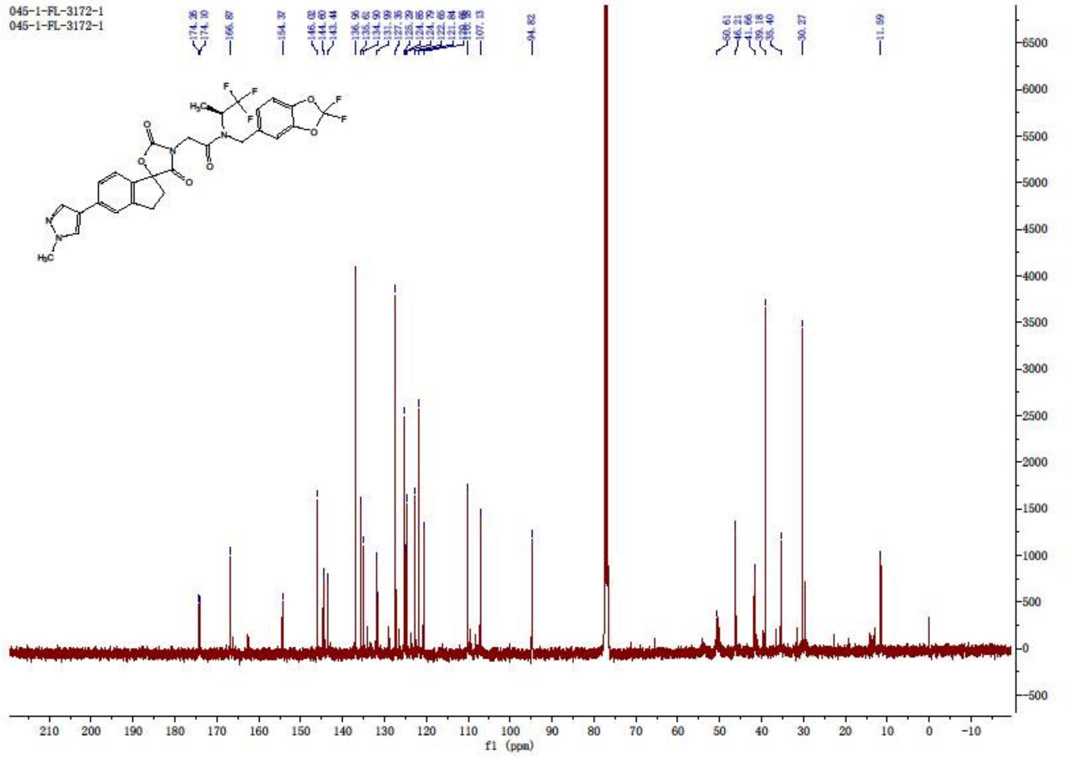

^{1}H NMR spectrum of compound **B2**

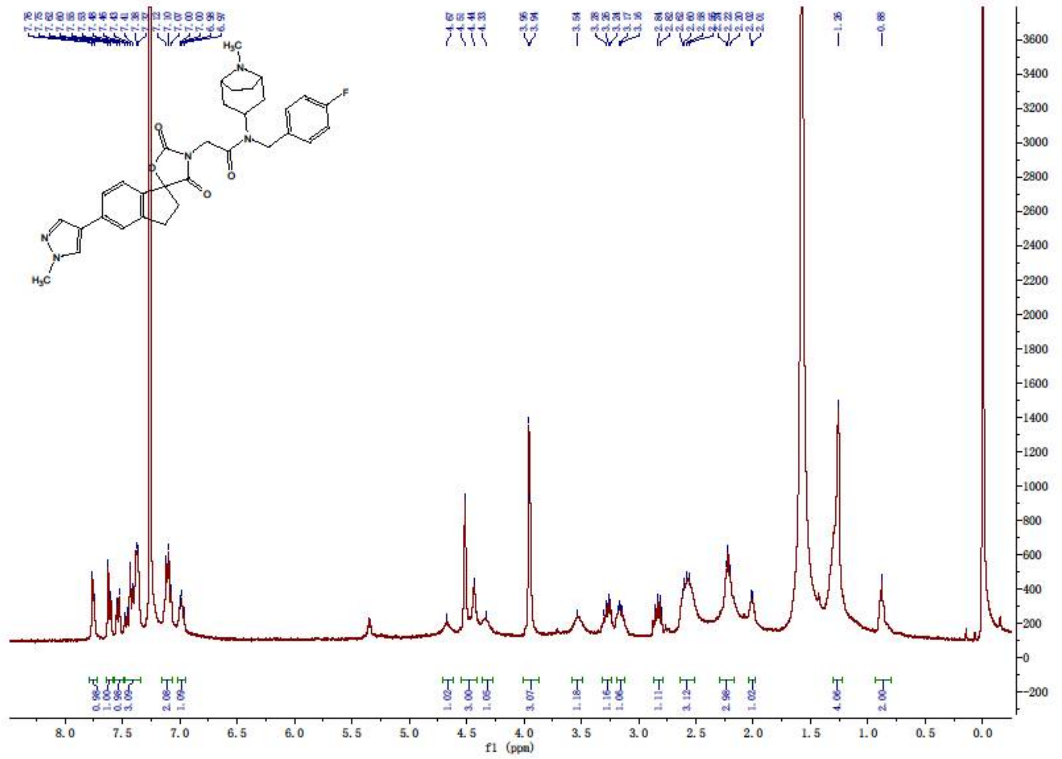

^{1}H NMR spectrum of compound **B3**

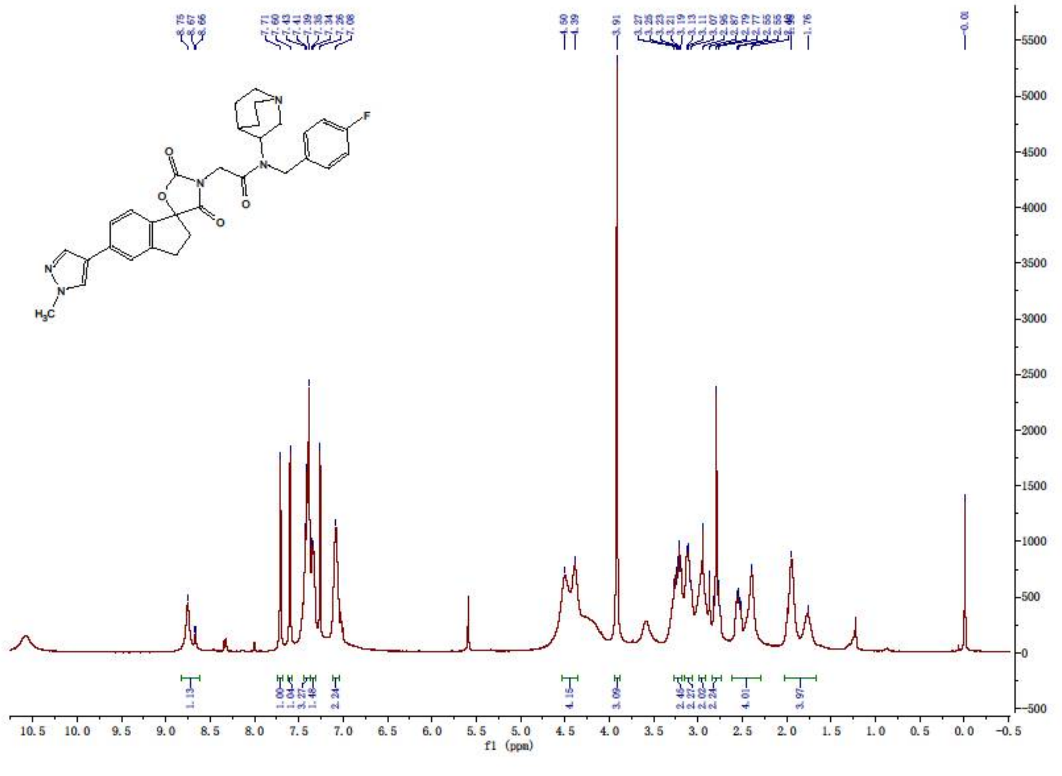

^{13}C NMR spectrum of compound **B3**

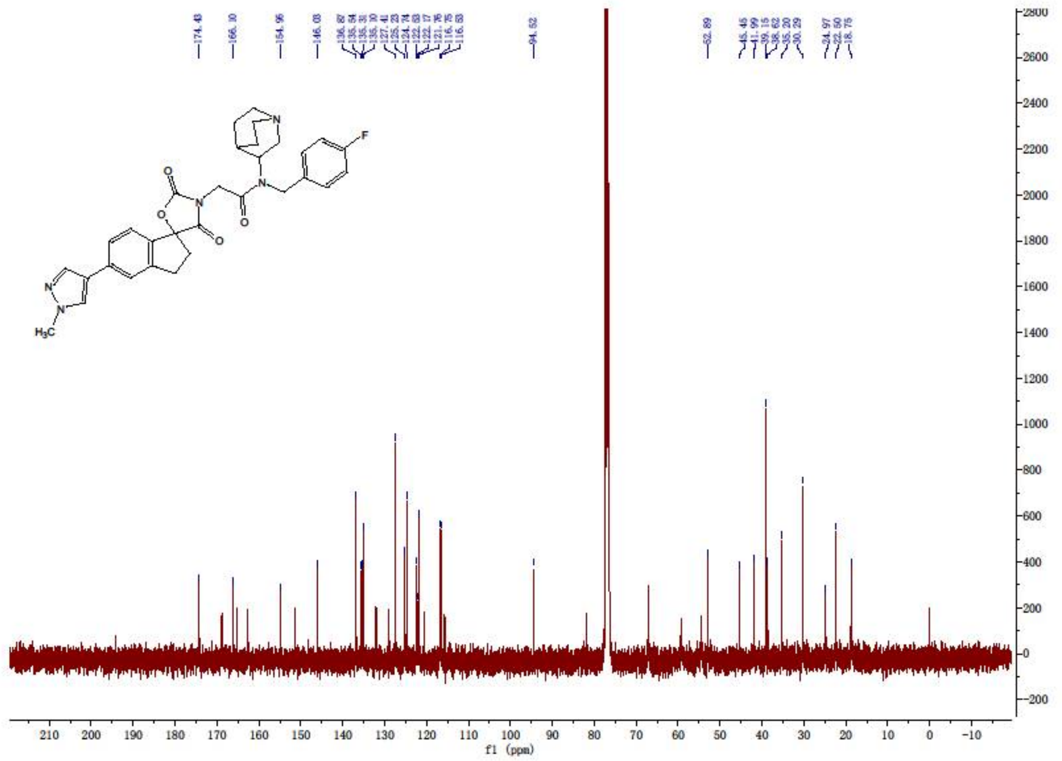

^{1}H NMR spectrum of compound **B4**

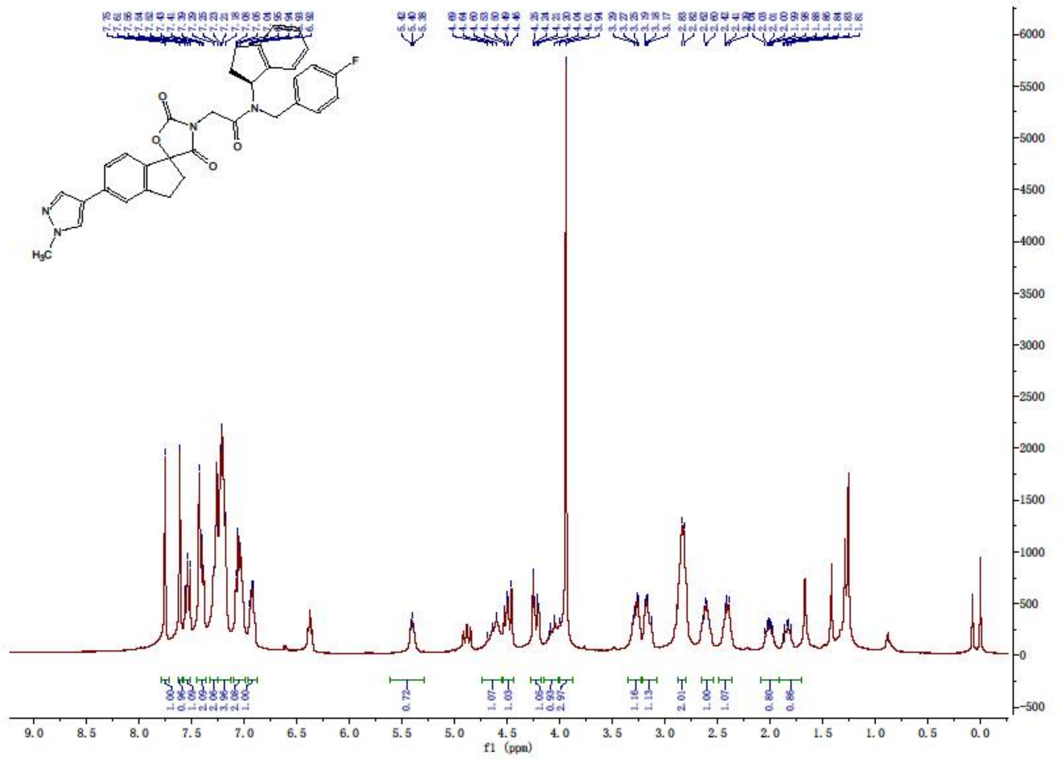

^{13}C NMR spectrum of compound **B4**

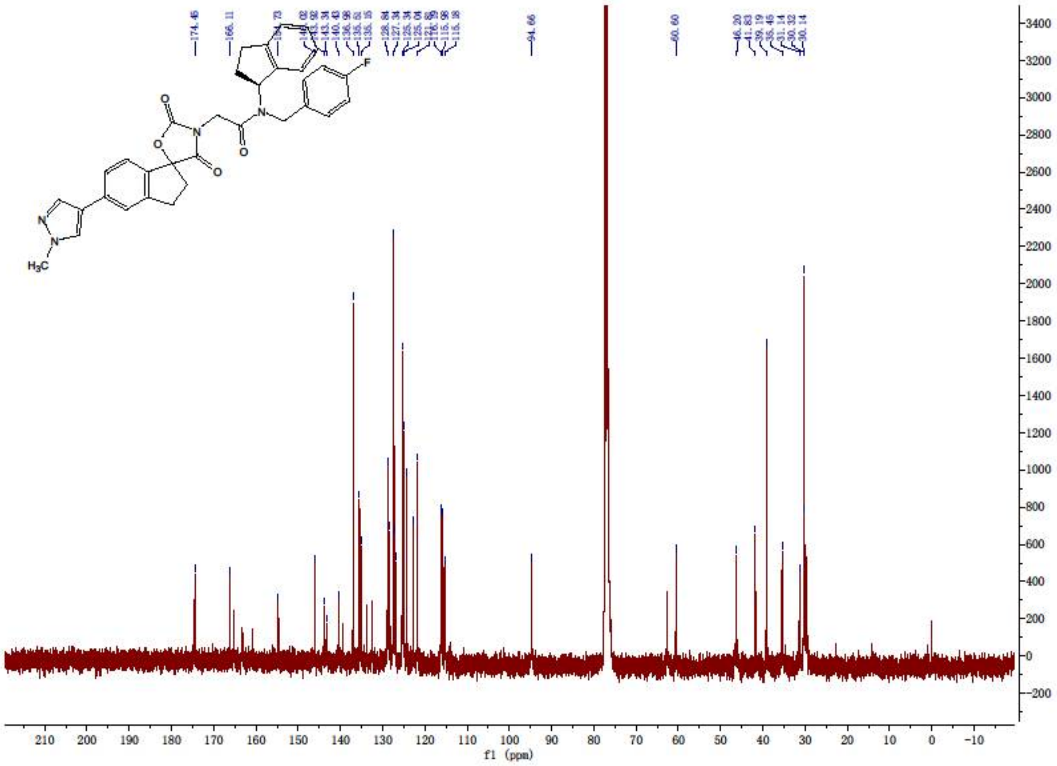

^{1}H NMR spectrum of compound **B5**

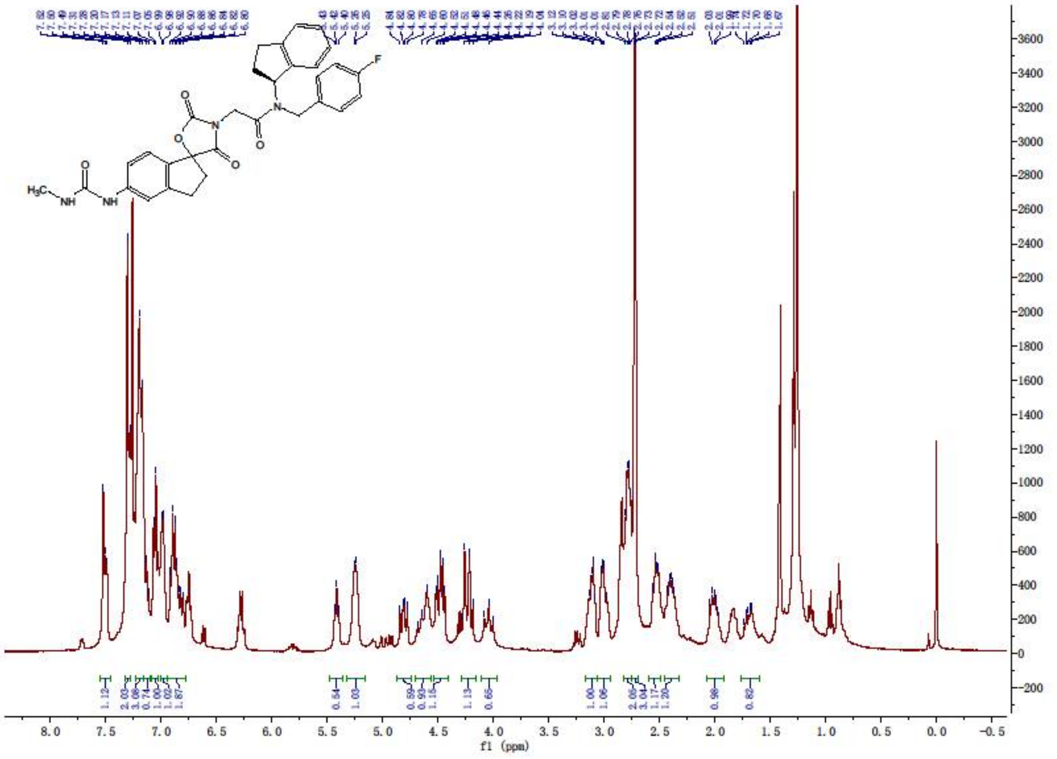

^{13}C NMR spectrum of compound **B5**

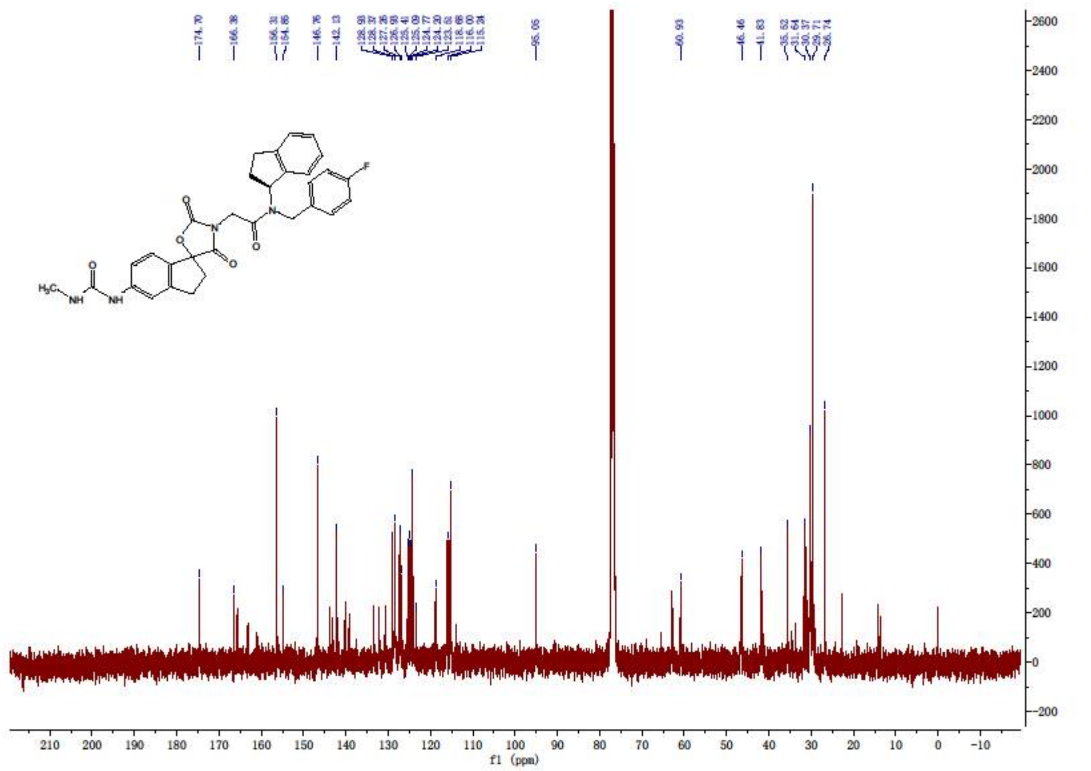

^{1}H NMR spectrum of compound **B6**

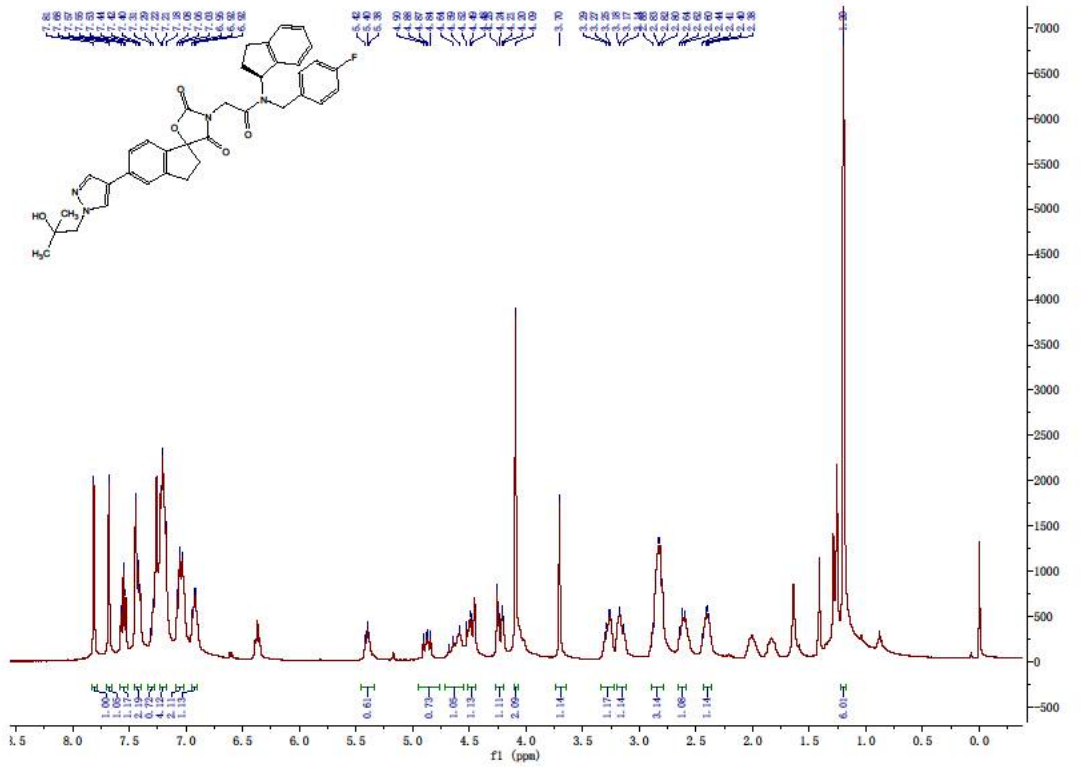

^{13}C NMR spectrum of compound **B6**

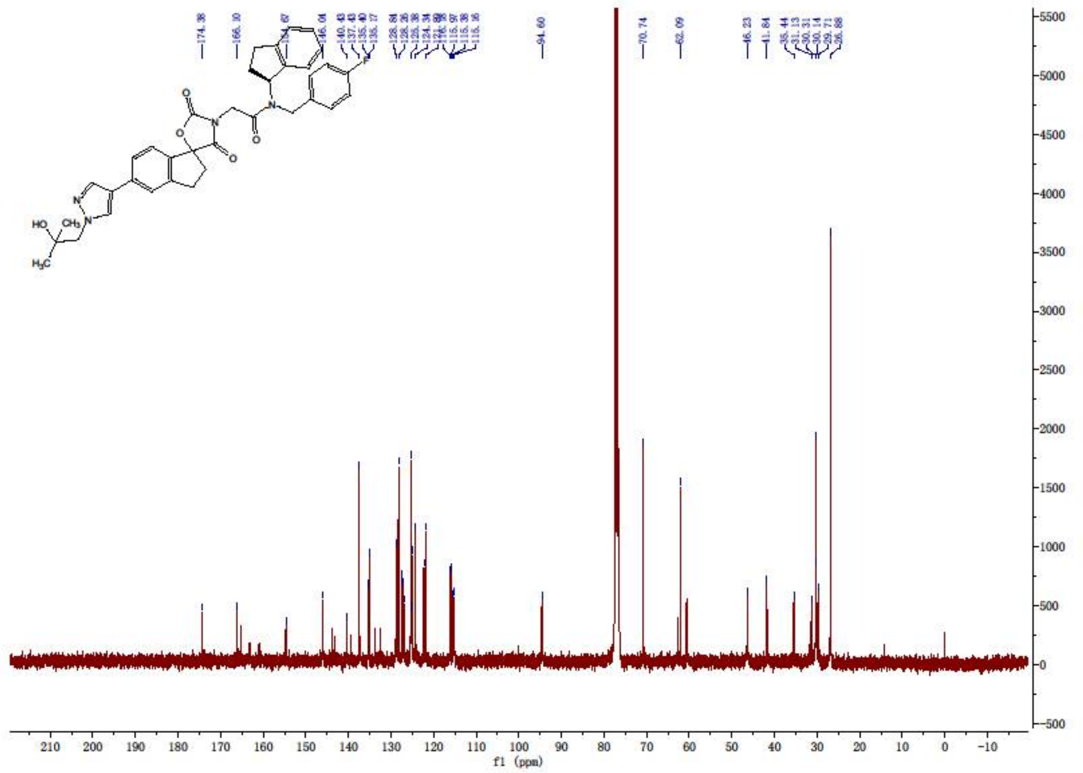

^{1}H NMR spectrum of compound **B7**

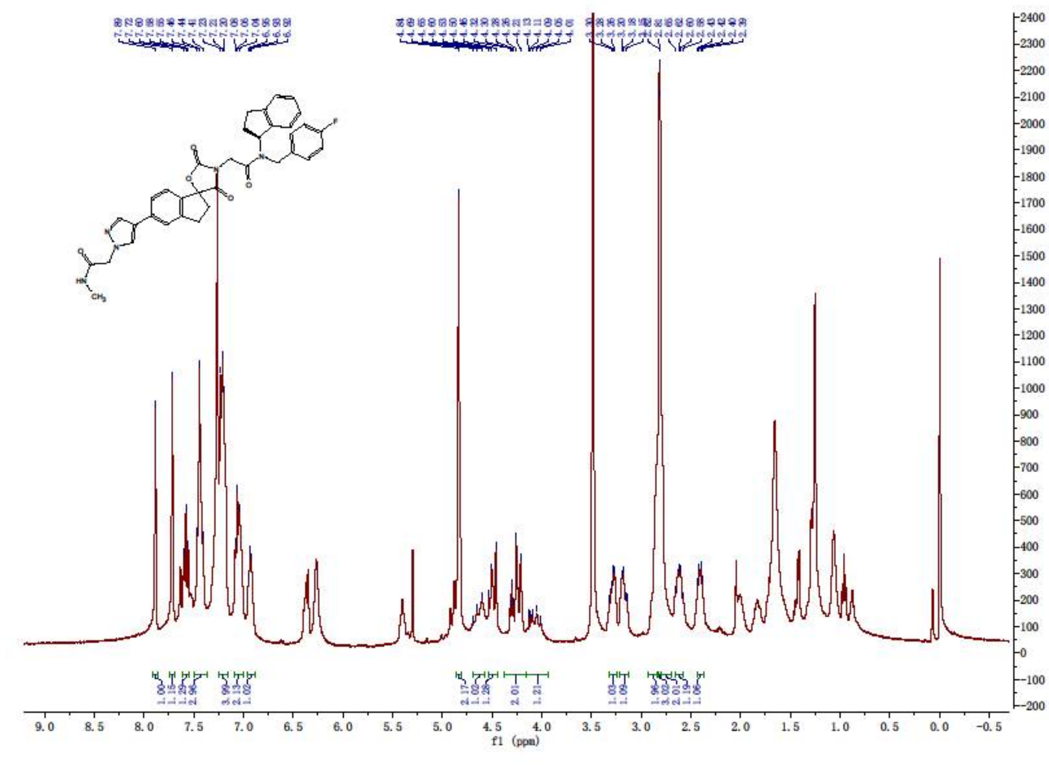

^{13}C NMR spectrum of compound **B7**

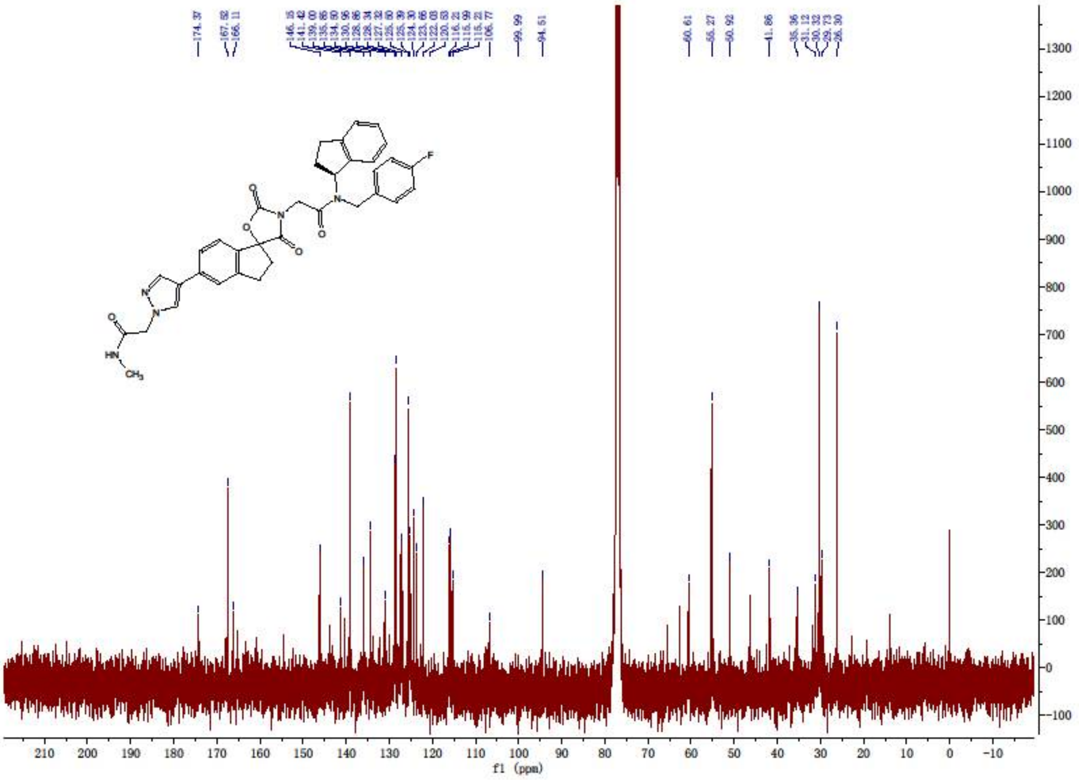

Supplement: Supplementary file 1 [file molecules-26-03162-s001.zip › molecules-1211127-supplementary.pdf]
